# Prefrontal cortex-dependent innate behaviors are altered by selective knockdown of *Gad1* in neuropeptide Y interneurons

**DOI:** 10.1371/journal.pone.0200809

**Published:** 2018-07-19

**Authors:** Katelynn M. Corder, Mariana A. Cortes, Aundrea F. Bartley, Samantha A. Lear, Farah D. Lubin, Lynn E. Dobrunz

**Affiliations:** 1 Department of Neurobiology, University of Alabama at Birmingham, Birmingham, Alabama, United States of America; 2 Department of Radiation Oncology, University of Alabama at Birmingham, Birmingham, Alabama, United States of America; McLean Hospital/ Harvard Medical School, UNITED STATES

## Abstract

GABAergic dysfunction has been implicated in a variety of neurological and psychiatric disorders, including anxiety disorders. Anxiety disorders are the most common type of psychiatric disorder during adolescence. There is a deficiency of GABAergic transmission in anxiety, and enhancement of GABA transmission through pharmacological means reduces anxiety behaviors. GAD67—the enzyme responsible for GABA production–has been linked to anxiety disorders. One class of GABAergic interneurons, Neuropeptide Y (NPY) expressing cells, is abundantly found in brain regions associated with anxiety and fear learning, including prefrontal cortex, hippocampus and amygdala. Additionally, NPY itself has been shown to have anxiolytic effects, and loss of NPY+ interneurons enhances anxiety behaviors. A previous study showed that knockdown of *Gad1* from NPY+ cells led to reduced anxiety behaviors in adult mice. However, the role of GABA release from NPY+ interneurons in adolescent anxiety is unclear. Here we used a transgenic mouse that reduces GAD67 in NPY+ cells (NPYGAD1-TG) through G*ad1* knockdown and tested for effects on behavior in adolescent mice. Adolescent NPYGAD1-TG mice showed enhanced anxiety-like behavior and sex-dependent changes in locomotor activity. We also found enhancement in two other innate behavioral tasks, nesting construction and social dominance. In contrast, fear learning was unchanged. Because we saw changes in behavioral tasks dependent upon prefrontal cortex and hippocampus, we investigated the extent of GAD67 knockdown in these regions. Immunohistochemistry revealed a 40% decrease in GAD67 in NPY+ cells in prefrontal cortex, indicating a significant but incomplete knockdown of GAD67. In contrast, there was no decrease in GAD67 in NPY+ cells in hippocampus. Consistent with this, there was no change in inhibitory synaptic transmission in hippocampus. Our results show the behavioral impact of cell-specific interneuron dysfunction and suggest that GABA release by NPY+ cells is important for regulating innate prefrontal cortex-dependent behavior in adolescents.

## Introduction

Anxiety disorders are the most prevalent group of psychiatric disorders found in adolescents [1]. Several studies have determined that manifestation and coping of anxiety disorders differ between children, adolescents, and adults [2–4]. Abnormalities in the GABAergic system are associated with a variety of neurological and psychiatric disorders including anxiety and mood disorders [5–9], schizophrenia [5,10,11], autism [7,12], Rett syndrome [13], and epilepsy [14]. In particular, deficits in GABAergic transmission are thought to underlie anxiety and depression in adults [8]. Many medications used to treat anxiety in adults—such as benzodiazepines—function by enhancing GABAergic synaptic transmission. However, pharmacologic treatment of anxiety in adolescents and children more commonly uses selective serotonin reuptake inhibitors, in part due to concerns about side effects from benzodiazepine use [8,15,16]. In adolescence, the GABAergic system is still developing [17], and the role of reduced GABA release in adolescent anxiety is not fully understood.

GABA is synthesized by the enzyme glutamate decarboxylase; the genes *Gad1* and *Gad2* encode its two isoforms, GAD67 and GAD65, respectively [18]. Polymorphisms in *Gad1* have been linked to anxiety disorders [19]. GAD67 is responsible for the majority of GABA production [20], and reduction of GAD67 has been shown to lead to reduced levels of GABA [20–22]. GABAergic cells—making up approximately 20% of all neurons [23]–are divided into several different subtypes of interneurons that are thought to play different roles in regulating behavior. Neuropeptide Y (NPY) interneurons are a subset of GABAergic interneurons that also are found abundantly in brain regions related to anxiety behavior such as hippocampus, prefrontal cortex, and amygdala [24,25]. In addition to releasing GABA, these interneurons also release NPY [26]. Acute application of NPY itself has been shown to exhibit robust anxiolytic effects, particularly in hippocampus [27,28], and it has been proposed to be a stress resilience factor [28–31]. Lesioning a subgroup of interneurons, of which a large portion were NPY+ cells, has been shown to increase anxiety-like behaviors in mice [32]. However, the contribution of GABA to the anxiolytic effects of NPY+ cells in adolescents is not yet known.

To test the effects of reducing GABA release from NPY+ cells, a transgenic mouse line (NPYGAD1-TG) is available that utilizes bacterial artificial chromosome-driven miRNA silencing of the gene *Gad1* under the NPY promoter [33]. This results in selective depletion of GAD67 in NPY+ cells [33]. Surprisingly, a previous study using this mouse model showed a decrease in anxiety-like behavior in adult mice [34]. The effect is likely specific to NPY+ interneurons, because altered anxiety-like behavior was not seen in mice with GAD67 reduced from cholecystokinin interneurons [34]. The reduced anxiety in adult NPYGAD1-TG mice could result from GAD67 depletion in hippocampus or prefrontal cortex, because knockdown of GAD67 only in amygdala does not alter baseline anxiety-like behavior, though it does ameliorate the anxiolytic efficacy of the benzodiazepine diazepam [35].

Here we used the NPYGAD-TG mice to test the importance of GABA release from NPY+ interneurons on anxiety and other behaviors in adolescent male and female mice (1–2 months old). We found alterations in innate behavior, specifically a mild increase in anxiety-like behavior (increased thigmotaxis in open field and increased avoidance of open arms of elevated plus maze), an increase in social dominance, and an enhancement in nest building. However, there was no alteration in fear learning tasks. There was a sex-dependent effect on locomotion / exploratory behavior in the open field, which was reduced in male NPYGAD1-TG mice but not females, potentially confounding the anxiety-like behavior shown in males. Immunohistochemistry revealed that the knockdown of GAD67 in NPY+ interneurons was incomplete at this age, and heterogeneous across regions. Although our results may underestimate the effects of GABA release from NPY+ cells on behavior, they show that GABA release from NPY+ interneurons is important for innate behaviors, particularly those associated with prefrontal cortex. In addition, our data suggest that GABA released from NPY+ cells plays a different role in adolescent anxiety-like behavior than what was previously shown in adults [34].

## Methods

### Animals

All experimental protocols were approved by the Institutional Animal Care and Use Committee at the University of Alabama at Birmingham (APN 20119) and were conducted in accordance with the *Guide for the Care and Use of Laboratory Animals* adopted by the National Institutes of Health. NPYGAD1-TG mice [33] were obtained from Dr. Karoly Mirnics. NPYGAD1-TG and NPYGAD1-WT mice were primarily bred as independent lines, and age-matched NPYGAD1-WT mice (non-littermates) were used as controls for this study. NPYGAD1-TG mice and NPYGAD1-WT controls were maintained on a C57Bl/6 background. Mice were group housed with 3–5 littermates/cage after they were weaned (p24-28). All group-housed animals were housed in single sex cages. Animals used for fear conditioning and nesting behavior were individually housed for the duration of the behavioral tasks. Mice were maintained in a room at 21± 2°C with food and water ad libitum and a 12 hour light/dark cycle (6:00 am to 6:00 pm). All experiments were conducted using both male and female mice, unless otherwise noted. Mice were used at 1–2 months of age (p28-p69), with the average age for each experiment falling between 5 and 6.5 weeks old. All behavioral tasks (with the exception of open field and nest building) were conducted between 7:00 am and 12:00 pm. Open field was conducted between 1:00 pm and 4:00 pm. Plasma and brain tissue were collected between 7:00 am and 10:00 am for electrophysiology recordings, immunohistochemistry, western blots, and ELISA.

### Behavior

Behavioral animals were handled for 4–5 minutes/day for a minimum of 3 days prior to testing. Nesting and fear conditioning mice were individually housed and allowed to adjust to the individual cage for 24–48 hours. All animals were habituated to the testing or holding room for 45–60 minutes before the start of the behavior experiments.

#### Elevated plus maze

Anxiety-like behavior was assessed using the elevated plus maze test. This is a cross plexiglass platform that is one meter high with two open arms and two closed arms. Low light (~ 9 lux) and generation of white noise were maintained for the duration of the test. During each experiment, mice were placed in the center of the maze facing an open arm and were allowed to explore for 5 minutes. Activity was tracked using video tracking software (Med Associates). Measured parameters included time spent in the open and closed arms, the total number of explorations into each arm, and the number of entries into the open and closed arms. The average age for tested animals was 36 ± 7 days.

#### Open field test

The open field test was used to measure locomotion and anxiety-like behavior. The open field apparatus is a square (27.9 cm X 27.9 cm x 27.9 cm) box with plexiglass sides consisting of 48 infrared beams and tracking software (Med Associates). All open field test experiments were conducted under fluorescent lights (~140 lux) with the presence of a white noise machine. Mice were placed in the same corner of the box and total movement (ambulatory distance) was measured for 5 minutes to assess differences in locomotion. Anxiety-like behavior was assessed automatically by the software, measuring distance traveled in both the central and peripheral zones over a 5 minute period and analyzing the percentage of distance traveled in the central zone. The average age for tested animals was 41.5 ± 8.4 days.

#### Nesting behavior

Nest building is a non-learned behavior that is dependent upon hippocampus [36,37] and prefrontal cortex [38]. The cage floor was covered with bedding to a depth of 0.5 cm. Two hours before the dark cycle began, mice were provided with pre-weighed, soft cotton nesting material (4 g; Ancare). The next morning, approximately two hours after the start of the light cycle, the cages were inspected for construction of nest using published criteria based on a five point system accounting for the percentage of the nesting material shredded and the overall construction of the nest [36,37]. Photographs were taken for documentation and scoring purposes. Two-three independent researchers—blind to the genotypes and sex of the mice—scored the nests from 1 to 5. These scores were averaged together for each mouse. The average age for tested animals was 43 ± 7.6 days.

#### Tube test

The tube test was used to measure social dominance. The protocol was adapted from a previously published protocol [39]. Mice were matched to two different age-matched partners of the same sex, but different genotype. No partners were from the same home cage. Each match was conducted by placing a mouse into each end of the tube. Sides were assigned at random. A loss was defined as four feet backing out of the tube. Though no draws occurred, a draw would have been declared after two minutes. Each mouse was tested two times with a different partner, with a minimum of ten minutes between tests. All tests were conducted between 9:00 am and 11:00 am in a separate, illuminated room from where the animals were housed. Mice were allowed to habituate to the new room for 60 minutes prior to the onset of testing. The tube was a 30.5 cm long vinyl tube with a 2.5 cm internal diameter, purchased from a local hardware store. The average age for tested animals was 52 ± 0.9 days.

#### Fear conditioning

For all fear conditioning experiments, subjects were placed in a 30.5 cm x 24.1 cm x 21 cm box with a clear plexiglass front, an electrifiable grid floor and a sound generator (Med Associates). Freezing behavior was automatically tracked via video tracking software (Video Freeze; Med Associates).

Contextual fear conditioning was assessed by placing subjects in the new context of the box for 5 minutes. During this time, they received 2 minutes to explore the box followed by 3 footshocks of 0.5 mA strength for a duration of 1 second, separated by a minute of no stimulation. After 24 hours, subjects were placed back in the same context with no footshock for a 5 minute period and freezing was measured. The average age for tested animals was 42 ± 9.8 days.

Cued fear conditioning was assessed by placing subjects in the new context of the box for 5 minutes. After a 2 minute exploration period, a 30 second tone was given. During the last 2 seconds of the tone, a 2 second 0.5 mA footshock was applied. This was repeated after 2 minutes of no stimulation. After 24 hours, the box was changed with the addition of colored panels and a plexiglass panel to cover the electrifiable grid in order to alter the context. Subjects were placed back in the box and subjected to the same auditory protocol as during acquisition without the footshock [40]. The average age for tested animals was 38.1 ± 7.4 days.

### Electrophysiological recordings

NPYGAD-TG or NPYGAD-WT mice (1–2 months old) were anesthetized using isoflurane and then decapitated with a guillotine. Brains were rapidly removed and 400 μm thick coronal slices of hippocampus were made using a vibrating microtome (Camden or Leica) using standard methods [41] in ice-cold (1–3°C) dissecting solution containing the following (in mM): 120 NaCl, 3.5 KCl, 0.7 CaCl_2_, 4.0 MgCl_2_, 1.25 NaH_2_PO_4_, 26 NaHCO3, and 10 glucose, bubbled with 95% O_2_/5% CO_2_, pH 7.35–7.45. Slices were held in a holding chamber containing the dissecting solution, bubbled with 95% O_2_/5% CO_2_ for >1 hour prior to recording.

Recording was conducted in a submersion recording chamber perfused with external recording solution containing (in mM): 120 NaCl, 3.5 KCl, 2.5 CaCl_2_, 1.3 MgCl_2_, 1.25 NaH_2_PO_4_, 26 NaHCO_3_ and 10 glucose bubbled with 95% O_2_ /5% CO_2_, pH 7.35–7.45. All experiments were conducted at 23–25°C. Synaptic responses were recorded in response to extracellular stimulation elicited using a bipolar tungsten microelectrode (FHC, Bowdoinham, ME) in stratum radiatum. Stimulus intensity ranged from 20 to 180 μA, and stimulus duration was 100 μs. The stimulating electrode was placed in s. radiatum to stimulate the Schaffer collateral axons.

#### Extracellular field recordings

To measure dendritic field excitatory postsynaptic potential (fEPSPs), a recording electrode was placed in stratum radiatum to record the response from Schaffer Collateral stimulation. Stimulation was generated from a Master-8-cp stimulator (A.M.P.I.) and applied with a WPI stimulus isolator (WPI). fEPSPs were recorded using glass micropipettes (2–5MΩ) filled with extracellular recording solution. The synaptic response was measured as the initial slope of the fEPSP. Before the input/output curve was determined, a 15 to 20 minute stable baseline was obtained by setting the stimulation intensity as 50% of the maximum synaptic response (defined as the largest fEPSP before population spikes are generated). Paired-pulse stimulation was applied during the stable baseline, using 30, 50, 100, 200, and 500 ms intervals. The stimulation paradigm was repeated at 0.1 Hz. Paired pulse ratios are calculated as the response 2 / response 1. The input/output curve was determined by measuring the slope of the fEPSP and plotting against the fiber volley.

#### Whole cell recordings

CA1 pyramidal cells were blindly patched on a Zeiss Examiner.A1 upright microscope. Neurons were patched in the voltage-clamp configuration and held at 0 mV using an Axopatch 200B (Molecular Devices). Patch electrodes (4– 6MΩ) were filled with internal solution composed of the following (in mM): 125 Cs-Gluconate, 0.6 EGTA, 1.0 MgCl_2_, 3 MgSO_4_, 25 HEPES, 10 Na-ATP, 0.3 GTP, 5 phospocreatine, pH was adjusted to 7.2 with CsOH. The internal solution also contained 10 mM Cs-BAPTA to block depolarization induced suppression of inhibition [42], and QX-314 (N-)2,6-dimethylphenylcarbamoyl(methyl) triethylammonium chloride (2 mM) to improve space clamp and reduce nonlinear effects caused by voltage-gated channels in dendrites while recording from the soma (Colling and Wheal, 1994). The access resistance and holding current (<200 pA) were monitored continuously. Recordings were rejected if either access resistance or holding current increased >25% during the experiment.

To isolate monosynaptic inhibitory postsynaptic currents (IPSCs), 10 μM NBQX (2,3-Dioxo-6-nitro-1,2,3,4-tetrahydr-obenzo(f)quinoxaline-7-sulfonamide) and 50 μM D-APV (D-2-amino-5-phosphonopentanoic acid) were added to the external recording solution. To obtain a stable baseline, the maximal IPSC was determined and stimulation was reduced to generate a response whose amplitude was 40 to 60% of the maximum IPSC. Paired-pulse stimulation at different intervals (in ms: 40, 60, 100, 200 and 1000) were applied and repeated 20–25 times for each interval at 0.07 Hz. For the monosynaptic IPSC input-output, the stimulus intensity of the threshold evoked response, referred to as 1x, was determined as the intensity which gave a response between 20 to 30 pA. Stimulation was increased in a multiplicative manner to develop the input-output curves.

### Western blot

For tissue used for western blotting, mice were anesthetized with isoflurane and euthanized by decapitation. The whole brain was removed and one hemisphere was placed in oxygenated (95% O_2_/5% CO_2_), ice-cold, dissection solution as described above. The hippocampus, amygdala, or medial prefrontal cortex were removed and flash frozen on dry ice and stored at -80°C.

Normalized proteins (15 μg/μl) were separated on 8% polyacrylamide gel, transferred onto an Immobilon-FL membrane using a turbo transfer semi-dry system (Biorad), membranes blocked in 1:1 PBST:Licor blocking buffer and probed with primary antibodies for GAD67 (Mouse Anti-GAD67, monoclonal antibody, clone 1G10.2; 1:1000 dilution; Millipore, cat # MAB5406, Lot # 2923238, Antibody Registry ID: AB_2278725) [43,44] and β-Actin (Rabbit Anti- β-Actin, monoclonal antibody; 1:1000 dilution; Cell Signaling, cat #4970), overnight at 4°C. Secondary goat anti-rabbit 700CW or donkey anti-mouse 800CW antibody (1:20,000; Licor Biosciences) was used for detection of proteins using the Licor Odyssey system and optical densities were determined using Image Studio Lite (Licor). GAD67 levels were normalized to Actin on the same membrane. To strip the membrane of antibodies, the membranes were incubated in 0.1 M NaOH, followed by three 10 minute washes in PBS with 0.1% Tween and blocked again for 1 h before re-probing with a different primary antibody.

### Immunohistochemistry

For tissue used for immunofluorescence, mice were anesthetized with isoflurane before being transcardially perfused with ice-cold PBS. After decapitation, the brain was rapidly removed and placed in 4% paraformaldehyde overnight at 4°C and washed 3 times for 5 minutes in PBS. The brains were then then transferred to 30% sucrose solution for 24–48 hours before being placed in a cryomold with OCT compound and stored at -80°C. Coronal cryosections (Leica) of 10 μm thick were obtained and used for immunofluorescence.

Sections were dried at room temperature and washed three times with PBS prior to sodium citrate antigen retrieval (10 mM Sodium Citrate, 0.05% Tween 20, pH 6.0). Sections were washed three times with PBS and incubated with block buffer (1x PBS with 1% BSA, 2% non-fat dry milk, 3.3% Tween 20) or in 10% Normal Donkey Serum (Vectashield) for two hours at room temperature. After blocking, sections were incubated overnight at 4°C in primary antibody. Primary antibodies used were 1:200 rabbit anti-Neuropeptide Y (Biosensis), 1:500 mouse anti-GAD67 (Millipore), 1:1000 chicken anti-GFP (Aves). The next day, sections were washed three times in PBS and incubated for 1 hour in the following secondary antibodies: 1:400 goat anti-chicken FITC (Aves), 1:400 donkey anti-mouse TRITC (Jackson ImmunoResearch), 1:400 donkey anti-rabbit TRITC (Jackson ImmunoResearch). After secondary incubation, sections were washed three more times with PBST before being cover-slipped with Vectashield hardset mounting medium with DAPI (Vectashield). Imaging was performed on a Nikon H600L microscope at 20X for hippocampal images and 10X for prefrontal cortex images.

For quantification of NPY+, GFP+, and GAD67+ cells in the CA1 of the hippocampus or the anterior cingulate cortex of prefrontal cortex, the percentage of immunopositive cells in the given area was determined in female mice (2–3 slices from each animal, n = 3 animals/group), Three hippocampal sections and three prefrontal cortex sections were selected per brain. Analyses were performed using ImageJ software (v2.0.0). Immunopositive regions were highlighted at a constant threshold across samples and regions of interests were formed around each cell. To count the percentage of GFP+ cells in a given region, the cell counter plug in was used to count the number of GFP+ cells that colocalized with NPY+ cells. To compare the intensity of florescence of GAD67 or NPY in NPY+ cells between NPYGAD1-WT and NPYGAD1-TG mice, images were set to a constant threshold and the Area, Mean, Integrated Density were measured and used in the following equation to find the Total Corrected Cell Fluorescence = Integrated Density–(Area of selected cell X Mean fluorescence of background readings) (modified from [45]).

### Enzyme-linked immunosorbent assay

Mice were anesthetized with isoflurane and euthanized by decapitation and trunk blood was collected and placed into tubes containing heparin, aprotinin, and DPP-IV. Samples were spun at 3,000 RPM for 15 min at 4°C in a microcentrifuge. Plasma was collected and frozen with dry ice. Samples were stored at -20°C. Neuropeptide Y and Corticosterone levels were measured using commercially available ELISA kits (EMD Millipore, EZHNPY-25K and Enzo, ADI-901-097, respectively).

### Statistical analysis

All statistics were performed using Origin software (Origin Lab Corporation, 2002), SPSS Statistics (IBM, 2015), or Prism (GraphPad, 2008). Data are presented as mean ± standard error of mean (SEM), except for the ages of behavioral experiments, which are mean ± standard deviation. The sample number (n) refers to cell or slice number for electrophysiological experiments and immunohistochemistry, and it refers to animal number for all other experiments. Statistical comparisons were made using a two-way ANOVA followed by a Tukey post hoc analysis for multiple comparisons to reduce the probability of Type I error, unless otherwise noted. When statistical analysis showed no sex-related differences, data from males and females were combined.

## Results

### Selective knockdown of GAD67 in NPY+ cells results in a mild increase in innate anxiety-like behavior

**Fig 1:**
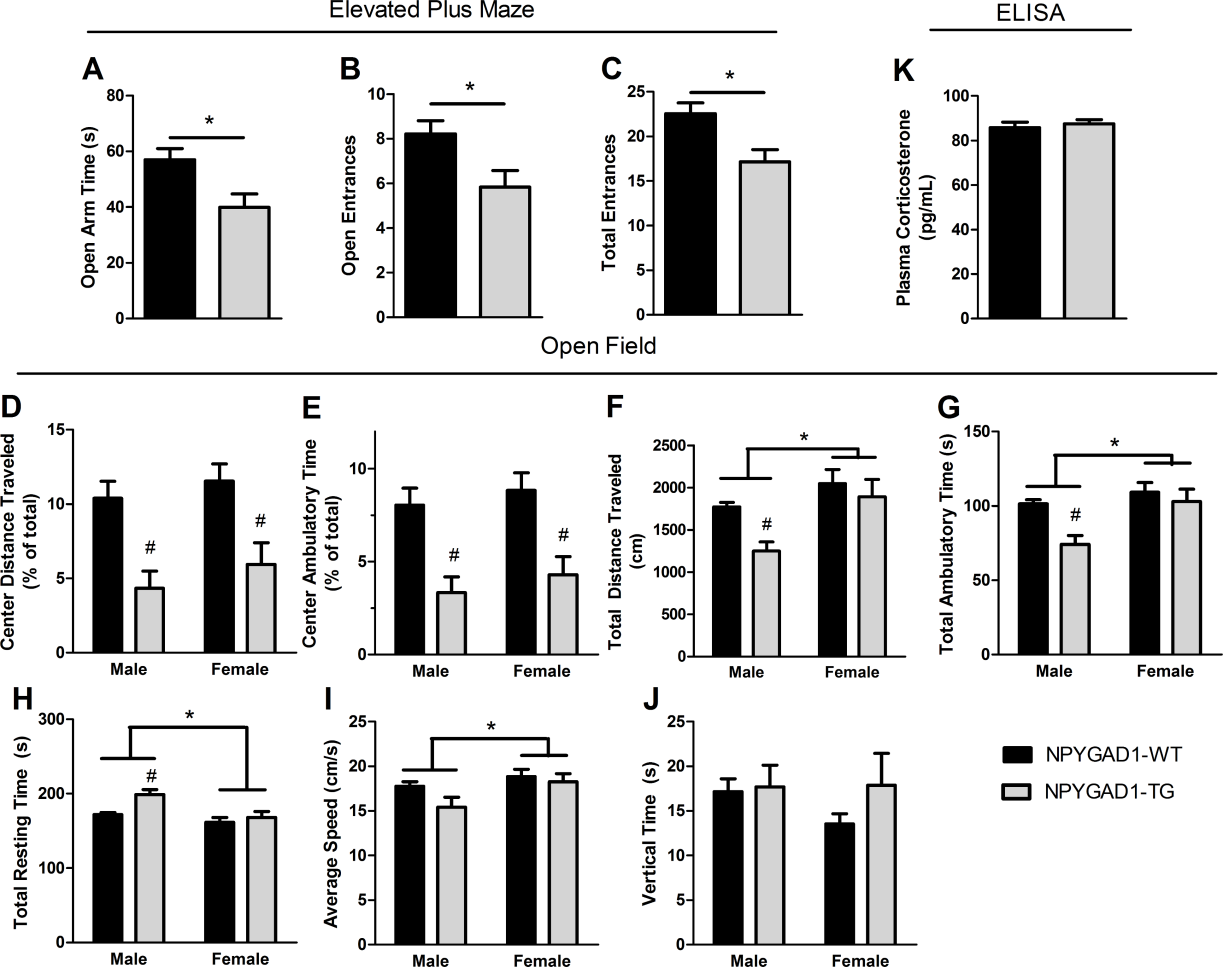
Innate anxiety-like behavior is increased in NPYGAD1-TG mice. (**A-C**) The elevated plus maze task showed a (**A**) decrease in time spent in the open arms of the maze, (**B**) fewer entrances into the open arms, and a (**C**) decrease in total arm entries. (**D-E**) A five-minute open field protocol measured locomotion and thigmotaxis. (**D**) Distance traveled in the central zone (as a percent of total) and (**E**) ambulatory time in the central zone (as a percent of total) were decreased in NPYGAD1-TG male and female mice compared to NPYGAD1-WT mice, with no sex-dependent difference. There was a genotype-dependent difference in (**F**) total distance traveled, (**G**) total ambulatory time, and (**H**) total resting time in males only. There was no genotypic change in (**I**) average speed in the open field, though there was a sex-dependent difference. There were also sex-dependent differences in (**F**) total distance traveled, (**G**) total ambulatory time, and (**H**) total resting time, with females showing higher activity levels. (**J**) Vertical time was unchanged by sex or genotype as was (**K**) basal plasma corticosterone levels. All statistical analysis was conducted using a two-way ANOVA followed by Tukey’s post hoc. Elevated plus maze and ELISA data are shown with sex groups pooled for simplicity as there were no significant differences found between sexes in any measure. # indicates significant difference from NPYGAD1-WT.

Previously, the NPYGAD1-TG mouse line was shown to have reduced anxiety-like behavior in adult mice [34], however here different results were observed in adolescent mice. In the elevated plus maze paradigm, both male and female transgenic mice showed a reduction in time spent in the open arms on the elevated plus maze (Fig 1A; NPYGAD1-WT n = 42; NPYGAD1-TG n = 22; F(1,63) = 6.566; p = 0.013) and decreased number of open arm entries (Fig 1B; NPYGAD1-WT n = 42; NPYGAD1-TG n = 22; F(1,63) = 6.025; p = 0.017). However, this was also accompanied by a decrease in total entries (Fig 1C; NPYGAD1-WT n = 42; NPYGAD1-TG n = 22; F(1,63) = 7.208; p = 0.009**),** which could indicate reduced locomotion or decreased exploratory behavior. Therefore, we next measured locomotion in the open field task and used thigmotaxis in the open field as a secondary anxiety task. The degree of thigmotaxis has been shown to be a measure of anxiety-like behavior in mice, independent of changes in locomotion [43,46]. We found a decrease in the percent of distance traveled in the central zone in both male and female NPYGAD1-TG mice (Fig 1D; NPYGAD1-WT n = 21; NPYGAD1-TG n = 19; F(1,39) = 22.69; p < 0.001), as well as a reduction in percent of ambulatory time in the center (Fig 1E; NPYGAD1-WT n = 21; NPYGAD1-TG n = 19; F(1,39) = 24.94; p < 0.001). There was no sex-dependent difference in the percent of distance traveled in the central zone or the percent of ambulatory time in the center (Fig 1E and 1F). These results show that there is increased thigmotaxis in both male and female NPYGAD1-TG mice compared to NPYGAD1-WT mice.

Interestingly, we found a genotype-dependent decrease in locomotor activity as shown by a decrease in total distance traveled (Fig 1F; NPYGAD1-WT n = 21; NPYGAD1-TG n = 19; F(1,39) = 6.04; p = 0.019), a decrease in total ambulatory time (Fig 1G: NPYGAD1-WT n = 21; NPYGAD1-TG n = 19; F(1,39) = 7.79; p = 0.001), and an increase in total resting time (Fig 1H; NPYGAD1-WT n = 21; NPYGAD1-TG n = 19; F(1,39) = 7.50; p = 0.010) in the open field. Tukey posthoc analysis determined that the differences in these locomotor measures were only present in males. These data indicate that there is a decrease in overall locomotor activity in male NPYGAD1-TG mice, but no genotype-dependent difference in overall locomotion in females. We further found a sex-dependent difference in total distance traveled (Fig 1F; Female n = 19; Male n = 21; F(1,39) = 10.99; p = 0.002), total ambulatory time (Fig 1G: Female n = 19; Male n = 21; F(1,39) = 9.25; p = 0.004), total resting time (Fig 1H; Female n = 19; Male n = 21; F(1,39) = 11.18; p = 0.002), and average speed (Fig 1I; Female n = 19; Male n = 21; F(1,20) = 5.27; p = 0.028) in the open field, with females exhibiting more overall activity than the males. Neither genotype nor sex had an effect on vertical time (Fig 1J; p>0.05). Taken together, our results show that loss of GAD67 from NPY+ cells causes reduced open arm time in the elevated plus maze, and increased thigmotaxis in the open field, suggesting a mild increase in anxiety-like behavior in adolescent mice. In addition, there is hypolocomotion in NPYGAD1-TG males compared to NPYGAD1-WT, which could indicate reduced exploratory activity in males. Interestingly, the increased anxiety-like behavior was not accompanied by an alteration in plasma corticosterone levels (Fig 1J; NPYGAD1-WT n = 10; NPYGAD1-TG n = 17; F(1,26) = 0.011; p = 0.920).

### Adolescent NPYGAD1-TG mice exhibit enhanced nest building behavior and social dominance

**Fig 2:**
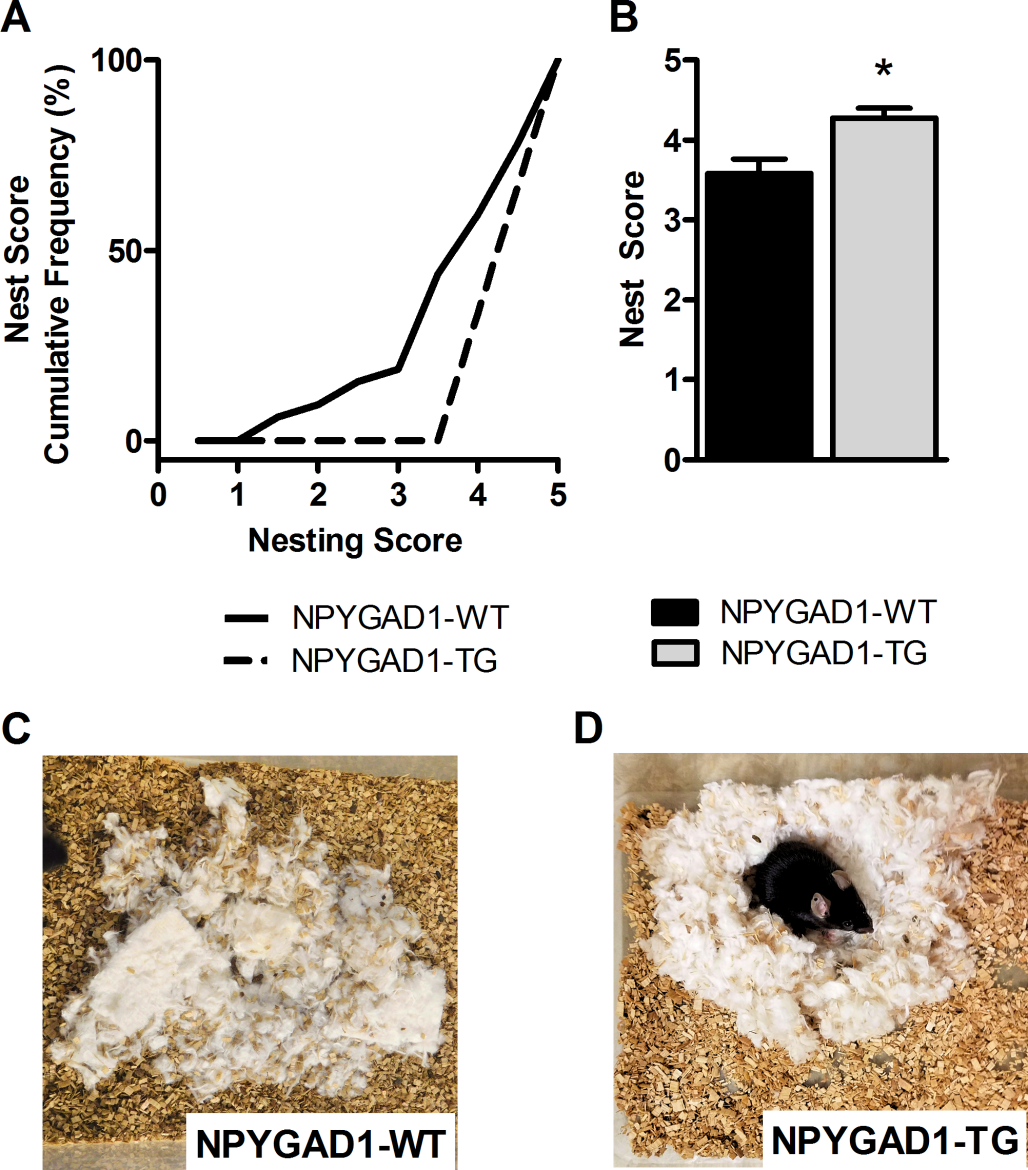
NPYGAD1-TG mice exhibit enhanced nest building behavior. (**A-B**) NPYGAD1-TG mice exhibited an increase in nesting scores, indicating that NPYGAD1-TG create more complete nests. **(A)** The cumulative frequency graph of nest scores shows a rightward shift in NPYGAD1-TG mice compared to NPYGAD1-WT. **(B)** There is an increase in the average nest score of NPYGAD1-TG mice. (**C-D**) Examples are shown of a NPYGAD1-WT nest (**C**), and NPYGAD1-TG nest **(D)**. Statistical analysis was done using a two-way ANOVA.

We next sought to evaluate other behaviors dependent upon brain regions associated with anxiety with the goal of determining which regions are contributing to this increased anxiety-like behavior. A total GAD67 knockdown in amygdala caused no changes in baseline anxiety-like behavior [35], so we focused on prefrontal cortex [47,48] and hippocampus [49,50]. We next measured nest-building, an innate behavior thought to be dependent upon hippocampus and prefrontal cortex. This task has been shown to be impaired by lesion of the whole hippocampus [51] or prefrontal cortex [38], and is considered a measure of cognitive well-being [52,53]. Nesting scores are decided by predetermined criteria [36,37] which evaluates the nest based on the percentage of the material shredded and the shape of the nest built. A score of 1 indicates that the nesting material was undisturbed. A score of 5 indicates complete shredding of the material and the construction of a full dome-like nest. Interestingly, we found an enhancement in nest building behavior in the NPYGAD1-TG mice, seen as both a rightward shift of the cumulative histogram of nesting scores (Fig 2A) and an increase in the average nesting scores (Fig 2B; NPYGAD1-WT n = 30; NPYGAD1-TG n = 16; F(1,45) = 4.958; p = 0.031). Example nests are shown from NPYGAD1-WT (Fig 2C) and NPYGAD1-TG (Fig 2D) mice, highlighting the difference in overall nest quality.

**Fig 3:**
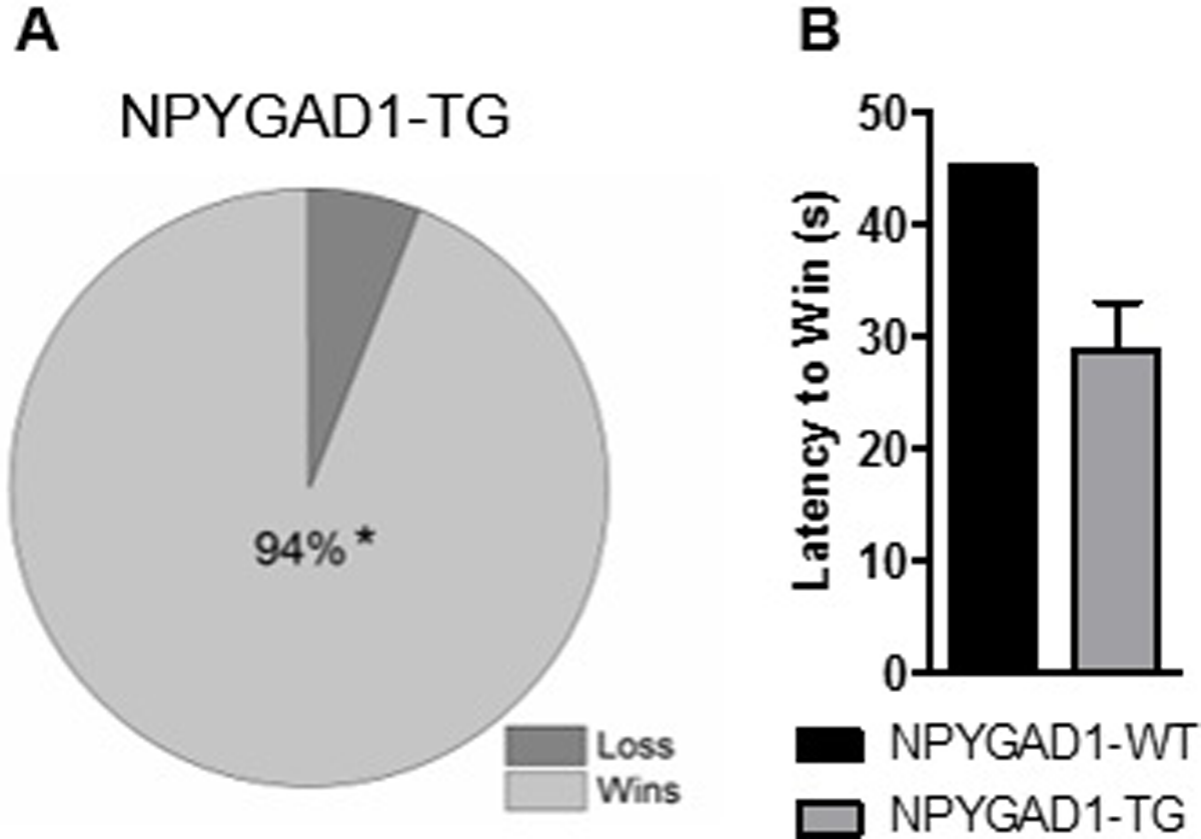
Social dominance is increased in NPYGAD1-TG mice. (**A**) NPYGAD1-TG mice exhibit increased social dominance behavior by winning 94% (15/16) of their matches against NPYGAD1-WT. Eight mice of each genotype were matched to two partners of the same age and sex of the opposite genotype for a total of 16 matches. Though, a time limit of two minutes was set for each match, most matches were over in less than 40 seconds as shown by (**B**) the average latency to win a match. Insets show schematics of experimental design paradigm for contextual fear conditioning **(A)** and cued fear conditioning **(B)**. Statistics were done using Chi squared analysis.

In addition to anxiety, prefrontal cortex has been associated with social behavior [54]. We next used the tube test to measure social dominance. This task is considered to be an innate behavioral task that is dependent upon the prefrontal cortex [54]. We found a strong phenotype of social dominance in the NPYGAD1-TG mice, with NPYGAD1-TG mice winning 94% of their matches against NPYGAD1-WT mice (15/16) (Fig 3A, 15/16 matches, 8 mice per genotype, χ^2^ = 12.25, p = 0.001), often in less than 40 seconds (Fig 3B). This suggests that GABA release from NPY+ cells in cortex is important for regulating social dominance. Combined with the anxiety behavior observed, this suggests that GABA release from NPY+ interneurons may play a role in innate behaviors at adolescent ages.

### Selective knockdown of GAD67 in NPY+ cells does not alter fear learning behavior

**Fig 4:**
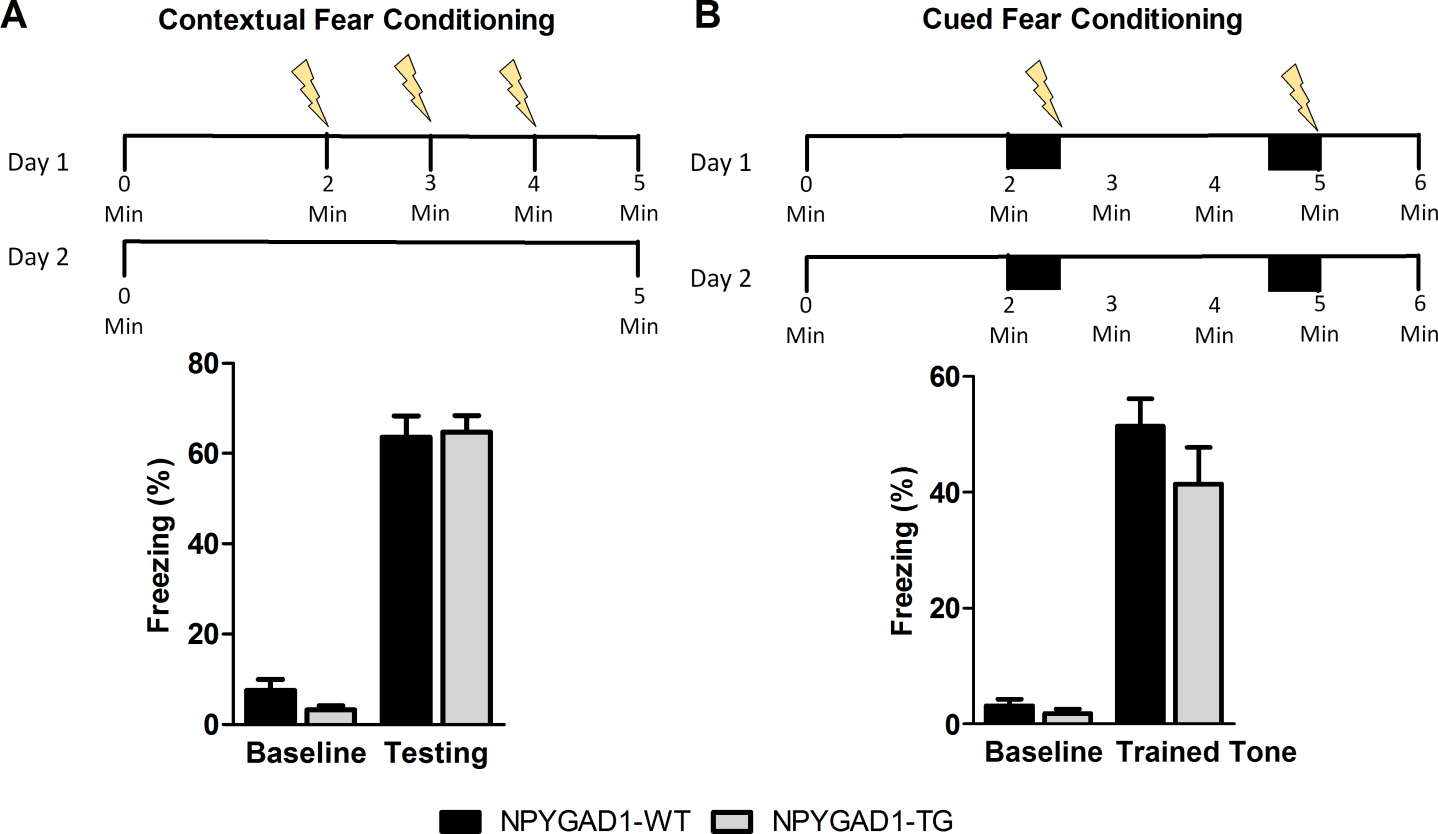
NPYGAD1-TG mice show no change in fear learning behavior. (**A**) No genotype-dependent fear conditioning alteration was seen in contextual fear conditioning, as shown by no difference in duration of freezing in response to the context on test day. (**B**) Likewise, there was no difference in cued fear conditioning seen in NPYGAD1-TG mice, as seen by no difference in duration of freezing in response to the tone on test day. Statistical analysis was conducted using a repeated measures two-way ANOVA.

We next sought to determine the effect of selectively knocking down GAD67 in NPY+ cells on fear learning behavior during adolescence. To assess this, we first compared contextual fear conditioning, a behavioral task that is hippocampal- and amygdala-dependent [55], between NPYGAD1-TG and age-matched wild-type controls. We found no change in fear memory consolidation after 24 hours (Fig 4A; NPYGAD1-WT n = 20; NPYGAD1-TG n = 16; F(1,32) = 1.308; p = 0.261) as indicated by a comparable increase in freezing times between groups when reintroduced to the context. We also investigated amygdala-dependent fear learning behavior to determine if this was altered alongside the anxiety-like behavior. We again found no difference between genotypes in cued fear conditioning (Fig 4B; NPYGAD1-WT n = 20; NPYGAD1-TG n = 12; F(3,84) = 1.308; p = 0.261). These results show that adolescent NPYGAD1-TG mice have no deficit in either hippocampal- or amygdala-dependent fear learning, and are consistent with previously published data in adult animals [34].

### NPYGAD1-TG mice have no reduction in total GAD67 protein levels in brain regions related to anxiety

**Fig 5:**
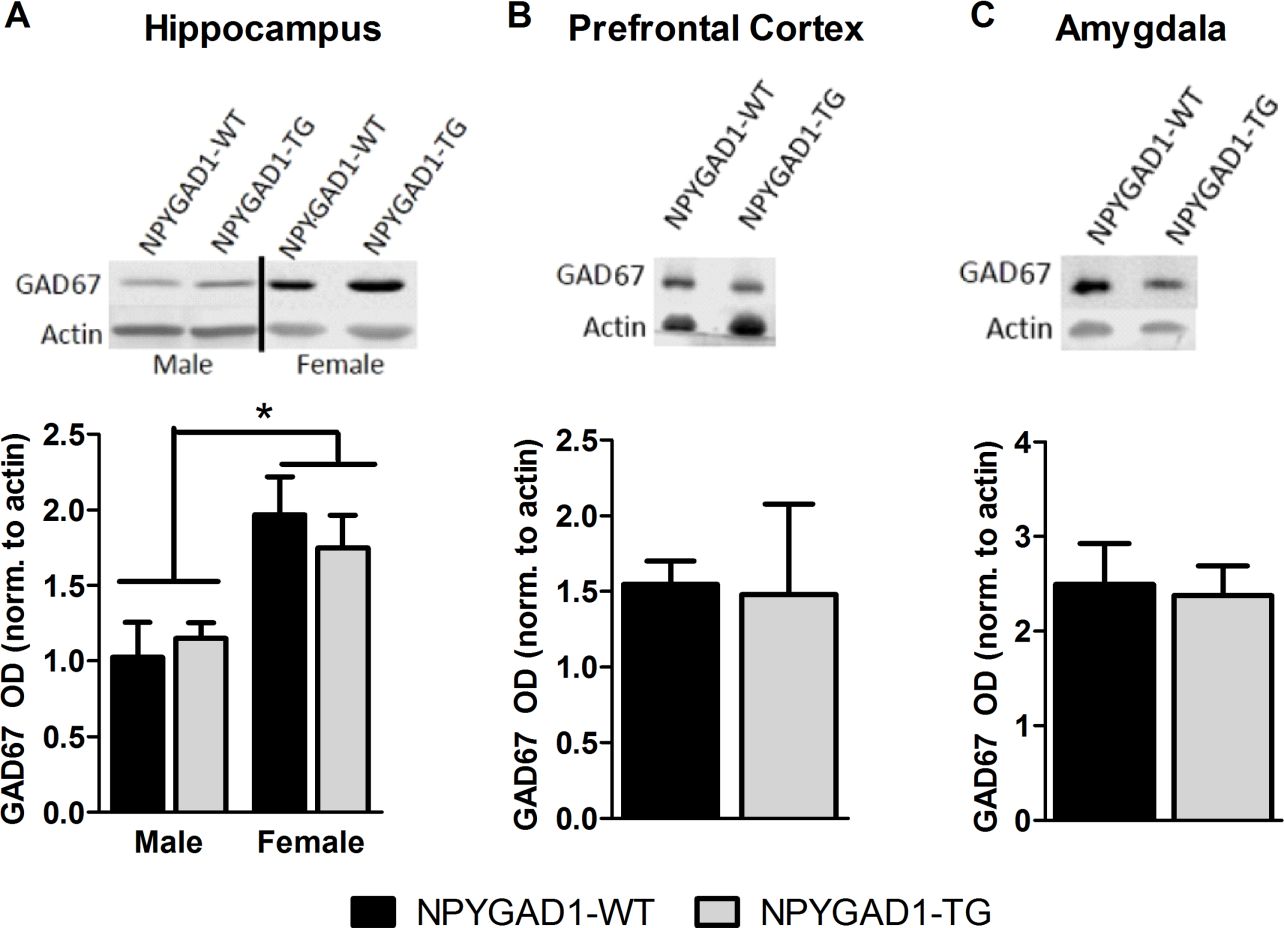
NPYGAD1-TG mice have no alteration in total GAD67 protein levels in brain regions related to anxiety. Western blot analysis showed no significant decrease in GAD67 protein levels in NPYGAD1-TG mice in (**A**) hippocampus, (**B**) prefrontal cortex, or (**C**) amygdala. However, there was an increase in GAD67 levels in females compared to males in hippocampus. There was no difference between males and females in prefrontal cortex so data were pooled. Only male samples were used in amygdala. Statistical analysis was conducted using a two-way ANOVA for hippocampus and prefrontal cortex, and one-way ANOVA for amygdala. Representative blots were cropped from the full images presented in S1 Data (hippocampus), S2 Data (prefrontal cortex), and S3 Data (amygdala).

We next used western blot analysis to measure GAD67 protein levels in whole hippocampus (Fig 5A), prefrontal cortex (Fig 5B), and amygdala (Fig 5C). There was a marked sex-related difference in GAD67 protein levels from whole hippocampal extracts, with higher GAD67 levels in female mice (males n = 10; females n = 9; F(1,18) = 14.078; p = 0.002). However, we found no genotypic difference in GAD67 protein levels from hippocampus (Fig 5A; NPYGAD1-WT n = 9; NPYGAD1-TG n = 10; F(1,18) = 0.053; p = 0.820), prefrontal cortex (Fig 5B; NPYGAD1-WT n = 11; NPYGAD1-TG n = 14; F(1,24) = 0.059; p = 0.811) or amygdala (Fig 5C, NPYGAD1-WT n = 6; NPYGAD1-TG n = 4; F(1,9) = 0.039 p = 0.364).

### NPYGAD1-TG mice show heterogeneous knockdown of GAD67 in NPY+ cells

**Fig 6:**
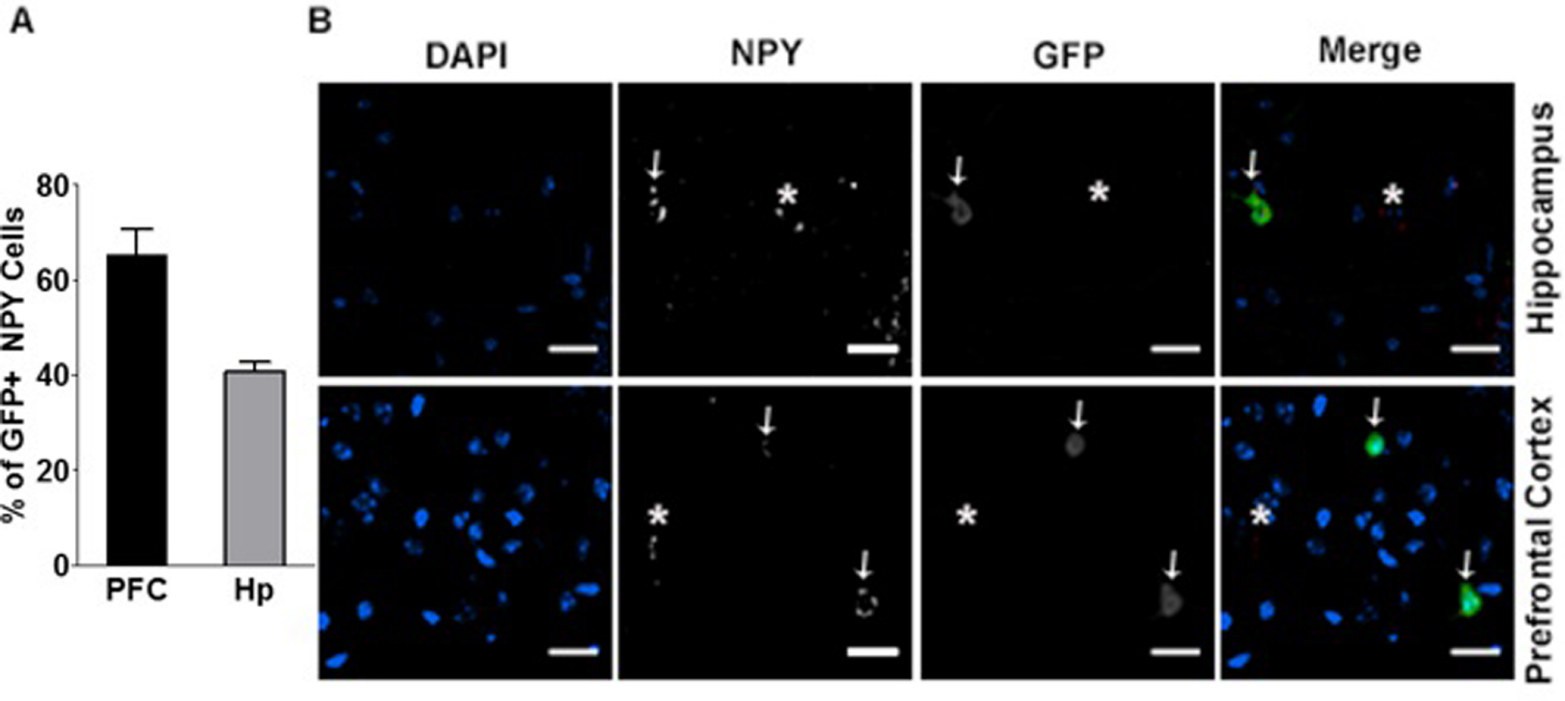
Expression pattern of transgene in NPYGAD1-TG mice. (**A-B**) The transgenic construct (as evidenced by the GFP tag) was shown to be present in some, but not all NPY+ cells in both the stratum radiatum of hippocampal CA1 and in the anterior cingulate cortex of prefrontal cortex. **(A)** Graph shows % of NPY+ cells that colocalize with GFP in prefrontal cortex (PFC) and hippocampus (Hp). **(B)** Example images are from NPYGAD1-TG prefrontal cortex and hippocampus. Merged images include GFP (green), NPY (red) and DAPI (blue). Scale bars: 25 μm. Representative images were cropped and enhanced for viewing from the full images, which are presented in S6 Data.

We next used immunohistochemistry to investigate the extent of GAD67 knockdown in the NPYGAD1-TG mice. First, we used immunohistochemistry to determine the extent of the transgene expression. Because the transgene contains a GFP tag, we immunostained slices for GFP and NPY and quantified the % of NPY+ cells that colocalize with GFP. We find GFP expression in 40% of NPY+ cells in hippocampus, specifically stratum radiatum of CA1, and in 65% of NPY+ cells in the prefrontal cortex, specifically anterior cingulate cortex (Fig 6A and 6B; NPYGAD1-TG, prefrontal cortex, n = 8 slices from 3 mice; NPYGAD1-TG, hippocampus, n = 8 slices from 3 mice).

**Fig 7:**
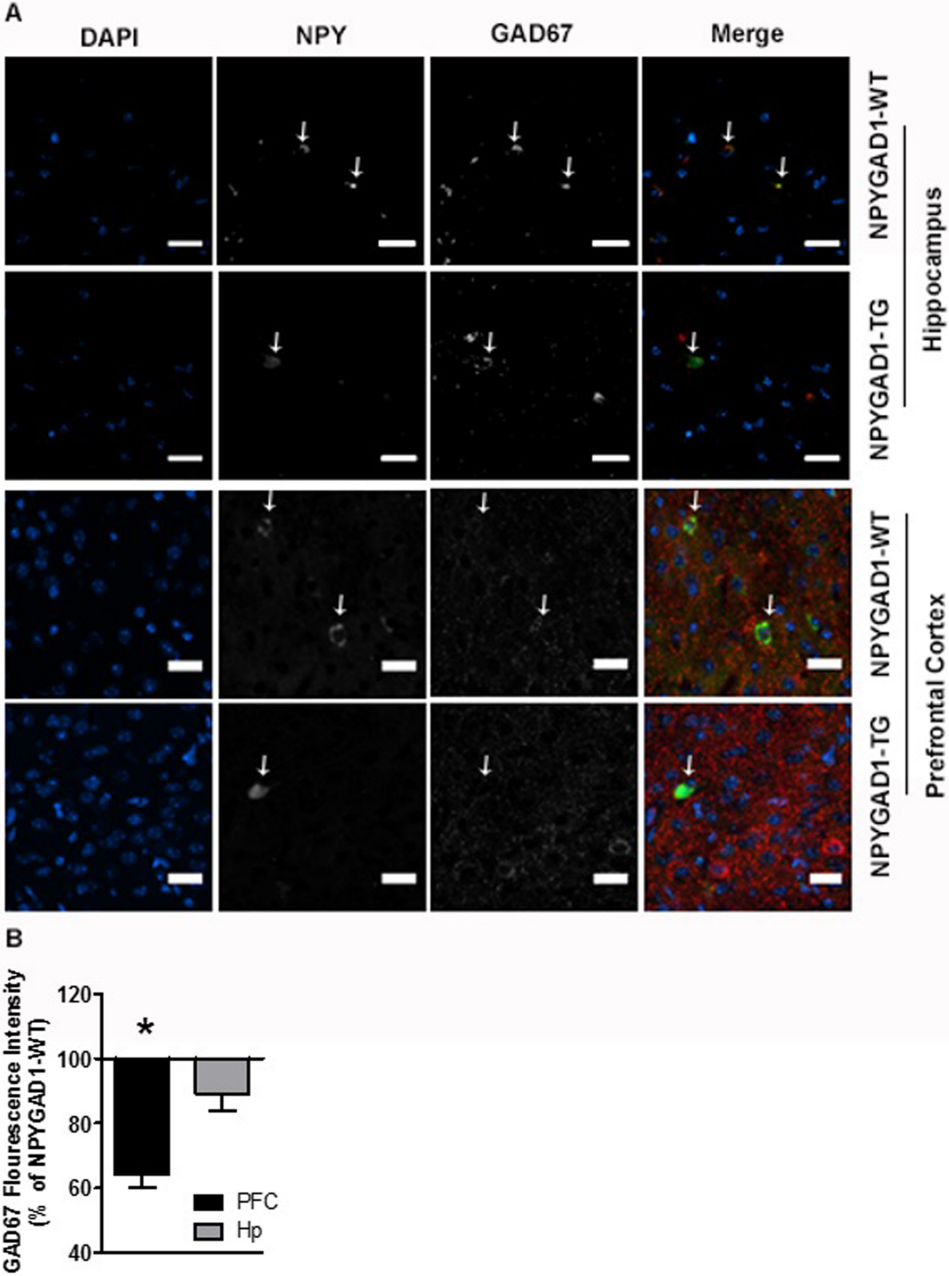
NPYGAD1-TG mice have heterogeneous knockdown of GAD67 in different brain regions. (**A**) Example images are from NPYGAD1-TG and NPYGAD1-WT prefrontal cortex (anterior cingulate cortex) and hippocampus (CA1 stratum radiatum). Merged images include NPY (green), GAD67 (red) and DAPI (blue). Intensity of GAD67 immunofluorescence was analyzed and compared between genotypes using a one-way ANOVA. Representative images were cropped and enhanced for viewing from the full images presented in S7 Data. **(B)** Fluorescence intensities of GAD67 in NPY+ cells are presented as percent of NPYGAD1-WT for hippocampus and prefrontal cortex.

We also stained for GAD67 and NPY, and tested the extent to which GAD67 was decreased in NPY+ cells by quantifying GAD67 fluorescence intensity in NPY+ cells from NPYGAD1-TG compared to NPYGAD1-WT. We found that there was a significant decrease in GAD67 levels within NPY+ cells in prefrontal cortex of NPYGAD1-TG sections as compared to NPYGAD1-WT sections (Fig 7B; prefrontal cortex, NPYGAD1-WT n = 7; NPYGAD1-TG n = 8 slices from 3 mice F(1,14) = 11.933; p = 0.004). Surprisingly, there was no GAD67 decrease in hippocampal NPY+ cells in NPYGAD1-TG sections (Fig 7B; hippocampus, NPYGAD1-WT n = 7; NPYGAD1-TG n = 8 slices from 3 mice F(1,14) = 0.836; p = 0.377), although there was a slight trend. Taken together, these data confirm that the transgene results in knockdown of GAD67 in at least a portion of NPY+ cells, but that the extent of the knockdown is heterogeneous between brain regions.

**Fig 8:**
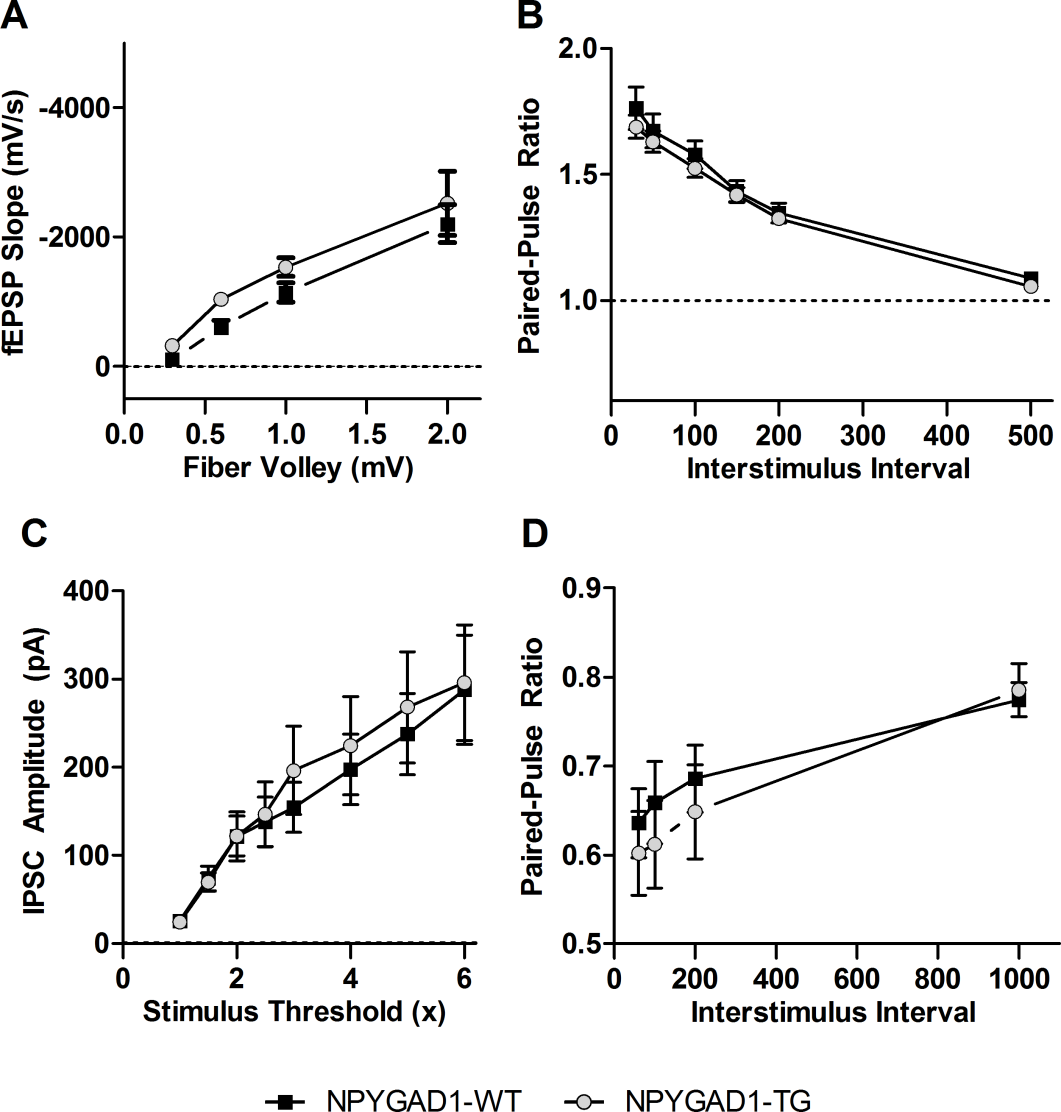
NPYGAD1-TG mice have no alteration in hippocampal CA1 synaptic transmission. (**A-B**) fEPSP recordings in the Schaffer Collateral pathway of hippocampal CA1 showed no difference in the input/output relationship **(A)** or paired-pulse ratio (**B**) in NPYGAD1-TG slices compared to NPYGAD1-WT slices. (**C**) Input/output curves for monosynaptic IPSCs recorded from dendritic targeting interneurons onto hippocampal CA1 pyramidal cells showed no difference in IPSC amplitude. (**D**) Paired-pulse ratio of monosynaptic dendritic IPSCs recorded hippocampal CA1 pyramidal cells showed no alteration in slices from NPYGAD1-TG mice. Statistical analysis was conducted using a two-way Repeated Measures ANOVA.

Because there was no decrease in GAD67 in CA1, we measured synaptic transmission in the Schaffer Collateral pathway of hippocampal CA1 to confirm a lack of an effect on GABAergic transmission. This pathway projects onto the pyramidal cells of CA1, which provide the primary output of hippocampus. Activating Schaffer Collateral axons stimulates both direct excitation and feed-forward inhibition onto CA1 pyramidal cells [56]. NPY+ interneurons account for approximately 30% of the interneuron population in hippocampal CA1 [57], suggesting that loss of GABA release from these cells is likely to decrease feed-forward inhibition and enhance excitation. To test this, we performed extracellular dendritic field recordings in the Schaffer Collateral pathway. We found no difference in the input-output curve (Fig 8A; NPYGAD1-WT n = 7; NPYGAD1-TG n = 6; F(1,36) = 3.077; p = 0.105) or paired-pulse ratio (Fig 8B, NPYGAD1-WT n = 18; NPYGAD1-TG n = 14; F(1.58,47.4) = 0.552; p = 0.539; Mauchly’s Chi-Square = 43.327, p<0.0001, Greenhouse-Geisser adjustment = 0.316). This is suggestive of no overall change in the synaptic transmission of this pathway.

To more directly test whether GABAergic synaptic transmission in CA1 was affected, we performed whole cell recordings of CA1 pyramidal cells and measured monosynaptic inhibitory postsynaptic currents (IPSCs) in response to stimulation of GABAergic axons, with excitatory receptors blocked. The stimulation electrode was placed close to the border of stratum radiatum and stratum lacunosum-moleculare to activate dendritic targeting axons from GABAergic interneurons [58], especially since NPY+ cells have been shown to preferentially target the dendrites [59]. There was no difference in the amplitude or paired-pulse ratio of IPSCs (Fig 8C, NPYGAD1-WT n = 7; NPYGAD1-TG n = 6; F(1.21,13.3) = 0.280 p = 0.649; Mauchly’s Chi-Square = 146.4, p<0.0001, Greenhouse-Geisser adjustment = 0.173 and Fig 8D, NPYGAD1-WT n = 7; NPYGAD1-TG n = 6; F(1.46,16.0) = 0.624, p = 0.5; Mauchly’s Chi-Square = 19.7 p = 0.002, Greenhouse-Geisser adjustment = 0.486). Together, these results show no significant decrease in inhibitory synaptic transmission in the Schaffer Collateral pathway, consistent with the immunohistochemistry data showing no significant GAD67 knockdown in CA1.

### There is no change in NPY expression in NPYGAD1-TG mice

**Fig 9:**
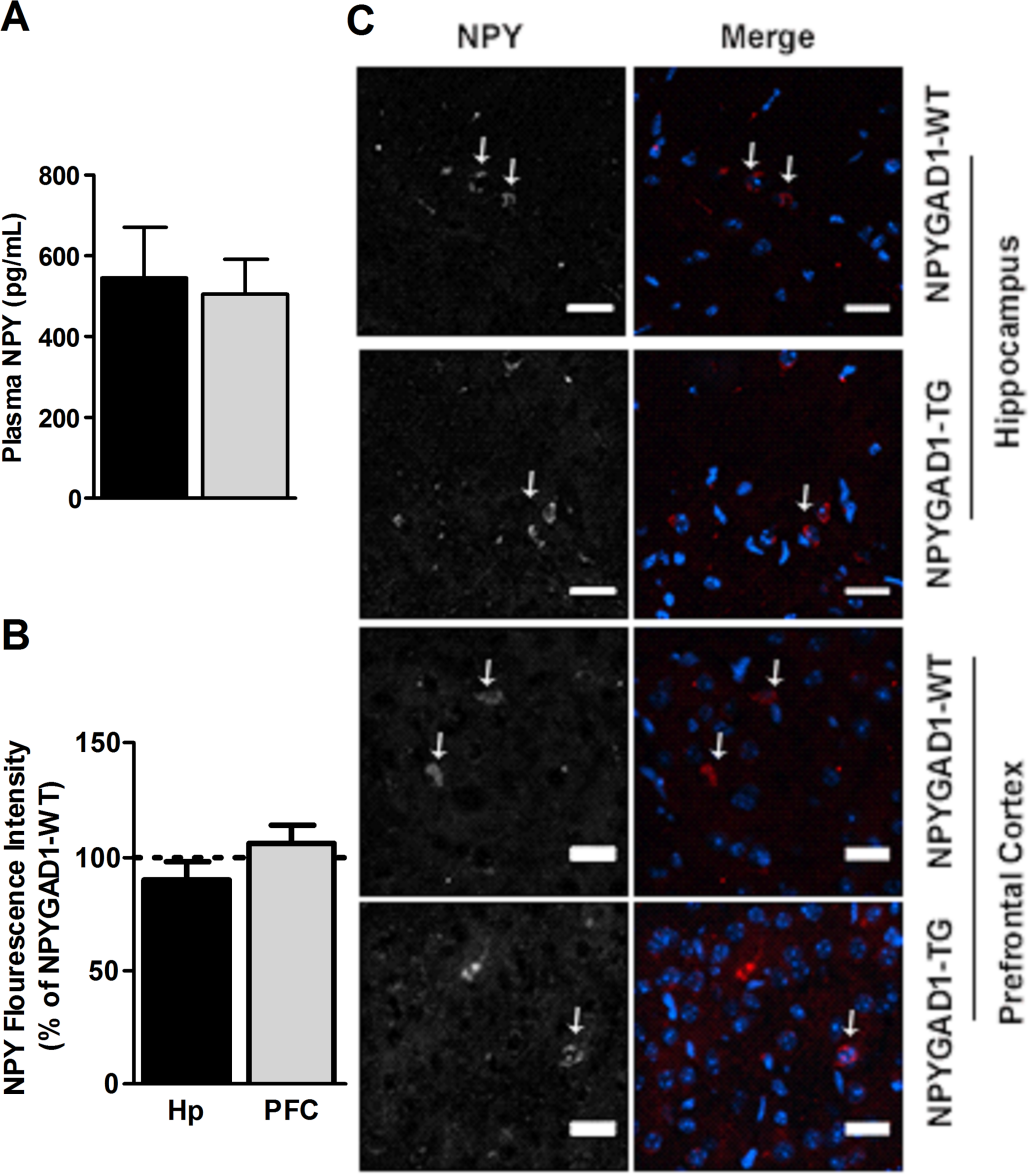
There is no alteration in NPY expression in NPYGAD1-TG mice. (**A**) There was no change in NPY protein levels in plasma measured with ELISA. Likewise, (**B**) immunohistochemical analysis of NPY expression showed no change in NPY in hippocampus (CA1) or prefrontal cortex (anterior cingulate cortex) from NPYGAD1-TG mice. Example images are shown in **(C)**. Merged images show NPY (red) and DAPI (blue). Scale bar: 25 μm. Representative images were cropped and enhanced for viewing from the full images, which are presented in S8 Data. Statistical analysis was conducted using a one-way ANOVA.

Because NPY itself has been shown to affect anxiety [28,49,60], we considered the possibility that a change in NPY release may accompany a decrease in GAD67 in NPY+ cells. To test whether the enhanced anxiety could potentially result from reduced NPY release [49], we used an ELISA to measure plasma NPY levels, and used immunohistochemistry to measure NPY levels in hippocampus and prefrontal cortex. There was no change in plasma NPY levels (Fig 9A; NPYGAD1-WT n = 7; NPYGAD1-TG n = 6; F(1,12) = 5.85E-04; p = 0.981). Similarly, there was no decrease in NPY levels in interneurons in prefrontal cortex (Fig 9B; NPYGAD1-WT n = 7 slices from 3 mice; NPYGAD1-TG n = 6 slices from 3 mice; F(1,10) = 2.501 p = 0.148) or hippocampus (Fig 9B; NPYGAD1-WT n = 5 slices from 3 mice; NPYGAD1-TG n = 8 slices from 3 mice; F(1,12) = 0.284 p = 0.604). These data indicate that the alterations in innate behaviors observed in adolescent NPYGAD1-TG mice were not secondary to changes in NPY levels.

## Discussion

In summary, we observed enhancement of anxiety-like behavior (increased thigmotaxis in the open field and increased avoidance of open arms in the elevated plus maze), nest building, and social dominance following the miRNA silencing of *Gad1* in NPY+ interneurons in adolescent mice. In contrast, there was no difference in either cued or contextual fear conditioning. The extent of GAD67 knockdown was heterogeneous, with decreased GAD67 observed in NPY+ cells of prefrontal cortex but not hippocampus. Because the knockdown of GAD67 is incomplete, results using these mice may underestimate the role of GABA release from NPY+ cells in behavioral tasks, particularly hippocampal-dependent behaviors. However, our results do support a role for inhibition from NPY+ cells in regulating innate behaviors dependent on the prefrontal cortex, specifically anxiety, nest building, and social dominance.

Anxiety behavior is regulated by multiple brain regions, including prefrontal cortex, amygdala and hippocampus [9,50,61]. However, previous studies have shown that knockdown of GAD67 in amygdala does not cause an increase in anxiety-like behavior in mice [35]. In addition, the enhanced anxiety-like behavior in adolescent NPYGAD1-TG mice is not likely to be caused by hippocampus, because there was little to no decrease in GAD67 in this region. Prefrontal cortex, and in particular the anterior cingulate cortex, has also been suggested to play a role in regulating anxiety [47,48,62], as well as social behavior [54]. In particular, lesioning of medial prefrontal cortex causes attenuation of anxiety-like behavior [62]. We see a significant reduction in GAD67 in NPY+ cells of prefrontal cortex, along with a strong enhancement of social dominance behavior. Although our results do not rule out a possible contribution from other brain regions, our data support the idea that prefrontal cortex plays an important role in the anxiety-like behavior of the adolescent NPYGAD1-TG mice.

The conclusion of an increase in anxiety-like behavior in the NPYGAD1-TG mice was based on the reduction in the percent of time spent in the open arm of the elevated plus maze (open arm avoidance), together with the decrease in the percent of distance traveled in the center of the open field maze (increased thigmotaxis). Both of these are well-validated tests for anxiety-like behavior in rodents [43,63,64]. There was also a decrease in the number of total entries on the elevated plus maze, which could potentially indicate a decrease in overall exploratory behavior that could confound our results. However, total arm entries has been noted to be controversial as a measure of motor activity, as it is indirect [63,65,66], and the use of distance traveled in the open field is a more direct measurement of locomotor activity [64]. We did observe a genotype-dependent change in the total distance traveled in the open field test in males, along with other measures of locomotor activity, indicating reduced locomotor activity in adolescent male NPYGAD1-TG mice compared to NPYGAD1-WT male mice. However, these effects were not present in females. This demonstrates that there is unlikely to be a locomotor impairment in the female adolescent NPYGAD1-TG mice and does not support a conclusion of reduced exploratory activity, suggesting a true increase in anxiety-like behavior in females. Similarly, no change in locomotor activity was observed in adult NPYGAD1-TG mice in a previous study [34].

The reduced locomotor activity in male NPYGAD1-TG mice is a potential confound that needs to be taken into consideration. There is controversy in the literature regarding the interpretation of changes in locomotion on the results of elevated plus maze and open field as tests for anxiety, particularly with respect to anxiotropic drugs [67]. Increased locomotion in response to stimulant drugs can confound the interpretation of the elevated plus maze, potentially producing false positives for anxiolytic-like effects [66]. Drugs causing sedation can severely reduce overall locomotion [68,69], also potentially confounding the interpretation. Because male NPYGAD1-TG mice have reduced locomotion, their increased thigmotaxis and open arm avoidance could be caused by reduced overall exploratory activity. However, in the absence of anxiotropic drugs, reduced locomotion may be an additional symptom of anxiety-like behavior [67,70], as reduced motor activity and increased immobility have been shown to be behavioral responses of frightened rodents [65,71]. It has been shown that thigmotaxis can be increased with the use of anxiogenic drugs and decreased with the use of anxiolytic drugs regardless of the effect on locomotor activity, since decreased locomotion was seen with both drug types [43]. Multiple studies have interpreted increased thigmotaxis as indicating increased anxiety even with the potential confound of locomotor changes [46,69,72–74]. Because NPYGAD1-TG males show a decrease in the percent of distance traveled in the center of the open field in addition to hypolocomotion, these data could indicate increased anxiety in male adolescent NPYGAD1-TG mice, as in females. Nevertheless, it is also possible that the results indicate a reduction in overall exploratory drive in males, rather than increased anxiety. Together, our results show that knockdown of GAD67 in NPY cells likely has a mild anxiogenic effect in female adolescent mice, and possibly in male adolescent mice.

In any case, it is striking that the anxiety-like behavior in these mice as adolescents does not mirror the reduced anxiety observed in adults [34]. This suggests a differential role of GABA release from NPY+ cells in regulating anxiety in adolescents compared to adults, highlighting the need for further study of the differential roles of GABA released from NPY+ cells in development. Previous studies have shown that there is a difference in anxiety-like behavior throughout rodent development [3,75]. In particular, anxiety is greater in younger animals and decreases as rodents mature into adulthood [75]. The adolescent age corresponds to a time period of continued maturation of the GABAergic system [17], and the effects of reduced GABA release on anxiety may be more pronounced in younger animals because anxiety is innately higher. Further, human studies have shown that adolescents and adults differ in coping mechanisms and responses to stressors [2,76]. It is possible that the observed difference in anxiety-like behavior between this study and previous studies in adults is due to differential compensatory mechanisms between age groups.

The apparent anxiogenic effect seen in adolescent NPYGAD1-TG mice was accompanied by a change in two other innate behaviors: nest building behavior and social dominance. Social dominance is a non-learned behavior dependent upon the prefrontal cortex [54]. We found an increase in dominant behavior of adolescent NPYGAD1-TG mice as measured by the tube test. Similarly, an increase in preference for social novelty was previously observed in adult NPYGAD1-TG mice [34]. A decrease in social dominance, rather than an increase, is more often seen in anxious mice [77,78]. However, aggression can also be comorbid with anxiety [79]. In addition, boosting excitability in prefrontal cortex has been shown to cause more dominant behavior and conversely, reducing excitability leads to a higher occurrence of subordinate behavior [78]. The augmentation of social dominance in NPYGAD1-TG mice is therefore consistent with a decrease in GABAergic transmission within prefrontal cortex, as evidenced by lower GAD67 fluorescence.

Nest building behavior is an innate, non-learned task that rodents perform regardless of sex, typically for thermoregulatory reasons [36,37]. Nest building can also be used as an indicator of cognitive well-being [51–53]. Previous studies have shown that there is a deficit in this task following whole hippocampal lesion [51], which may also be dependent on prefrontal cortex [38,62]. We saw an enhancement in nest building behavior in adolescent NPYGAD1-TG mice. This effect would not necessarily be observed in older age groups, since mice build better (more complete) nests as they reach adulthood with scores frequently averaging on the higher end of the scale [36,37]. While the correlation between poor nest building and impaired hippocampal function has been well established [36,52,58], the interpretation of enhanced nesting scores is less established. It is notable that the use of nest building as a measure of cognitive well-being comes primarily from studies with animals exhibiting impaired nesting [36,37,52,53]. Higher nesting scores compared to controls are thought to represent an increase in compulsive-type behavior [80], suggesting that GABA release from NPY+ cells may help reduce compulsive behavior and be implicated in a rodent correlate of obsessive-compulsive disorder—a subgroup of anxiety disorders as classified by the DSM-5. However, the enhanced nesting is unlikely to be due to altered hippocampal function as we see no change in hippocampal GAD67 and no change in synaptic transmission. Our results are consistent with a role for prefrontal cortex in nest-building behavior, although we cannot rule out a contribution of other brain regions. In addition, our results show that it can be useful to test for changes in nesting behavior in younger animals where both increases and decreases in nest scores can be detected.

In contrast to the innate behavioral effects observed, we found no changes in fear learning in the adolescent NPYGAD1-TG mice, in either the contextual fear conditioning or the cued fear conditioning paradigms. This was consistent with data obtained in adult mice of this mouse line [34]. Other studies involving a wide range of alterations in the GABAergic system have also not shown changes in fear learning [34,35,81]. In particular, knockdown of GAD67 in amygdala did not cause changes in cued fear conditioning [35]. Surprisingly, at this age, the NPYGAD1-TG mice have no decrease in hippocampal GAD67 or CA1 inhibitory synaptic transmission. As a result, studies using these mice may underestimate the effects of GABA release from hippocampal NPY+ cells.

Interestingly, this enhancement of anxiety-like behavior was not accompanied by a change in baseline corticosterone. Animals with enhanced anxiety often have increased basal corticosterone, indicating activation of the hypothalamic-pituitary-adrenal axis [82]. However, increased anxiety-like behavior with no change in basal corticosterone has also been observed in some mouse models [83,84]. While future studies could test for differences between genotypes in corticosterone in response to a stressor [84,85], our results indicate that the enhanced anxiety-like behavior is not secondary to baseline increases in corticosterone.

It is not known whether altering GABA release from NPY+ cells also leads to changes in NPY release, either due to altered firing of the NPY+ cells or as a potential compensatory mechanism. However, we found no change in NPY levels in tissue or plasma in adolescent NPYGAD1-TG mice. Our results suggest that in adolescent mice these cells are not compensating for a decrease in GABA production via altered NPY output. It remains to be seen if the reduction in GAD67 in NPY+ cells in NPYGAD1-TG mice as adults leads to enhanced NPY release, which could contribute to the unexpected decrease in anxiety behavior [28,49,86].

We saw no differences between male and female mice in any of the behavioral tests, in either genotype, with the exception of activity levels in the open field. Here males were less active than females overall, and also showed genotypic changes, with NPYGAD1-TG male mice less active than their wild-type counterparts. This suggests that overall anxiety-like behavior and fear learning are not different between males and females at this age. In contrast, sex-related differences in these behaviors have been seen in adult animals [80,87]. One caveat of this study is that we did not control for estrus cycle in the female mice, although adolescent mice are only just obtaining sexual maturity at the ages we studied. The only other significant sex-dependent difference we observed was that hippocampal GAD67 levels were higher in female mice than male mice, independent of genotype. Previous studies have shown a relationship between estrogen and GAD67 expression and the GABAergic system [88–90], which could contribute to this difference. It is possible that the enhanced GAD67 in females eventually contributes to the sex-specific behavioral differences that are observed in adult mice. Future expansion on this finding, including the study of females from this line in adulthood, could shed more light on the mechanisms underlying this phenomenon.

Because the NPYGAD1-TG and NPYGAD1-WT mice were bred as separate lines, experiments in our study used age-matched wild type mice on the same genetic background, rather than littermates, as controls. Although littermates are often used as controls when possible, this is not always the case [91–93]. It is possible, therefore, that differences in home cage environment or parental care could contribute to the observed behavioral differences in our study. It has previously been shown that adult NPYGAD1-TG mice have less anxiety-like behavior [34], so the increased anxiety-like behavior observed in adolescent NPYGAD1-TG mice is not caused by increased anxiety in their parents. Previous studies have shown that differences in specific forms of maternal behavior in rodents, such as licking, grooming, and arched-back nursing, correlate with differences in anxiety-like behavior and hippocampal expression of glucocorticoid receptors in their offspring [94,95]. It is not known whether NPYGAD1-TG mice have differences in specific maternal behaviors such as licking and grooming that could influence the development of neural circuits involved in fearfulness. Future studies could test for differences in parental behavior of NPYGAD1-TG mice, and whether this affects the behavior of the offspring.

This study sought to determine the importance of GABA release from NPY+ cells in anxiety in adolescent animals. We found evidence for increased anxiety-like behavior in NPYGAD1-TG animals–particularly in females—as well as an enhancement in nest building behavior and social dominance. This suggests a role of GABA release from NPY+ cells, particularly in prefrontal cortex, in regulating innate behaviors. As a result, therapeutic strategies that increase GABA transmission specifically from NPY+ cells could potentially be beneficial in treating anxiety and compulsive-type behavior in adolescents. In addition, this study highlights the importance of further studying adolescent behavior in animal models.

## Supporting information

S1 Data Raw western blot images for hippocampus to accompany Fig 5. (TIF)

S2 Data Raw western blot images for prefrontal cortex to accompany Fig 5. (TIF)

S3 Data Raw western blot images for amygadala to accompany Fig 5. (TIF)

S4 Data Raw data sheets for Figs 1–5 and 8. (XLSX)

S5 Data Raw data sheets for Figs 6–7 and 9. (XLSX)

S6 Data Raw images for Fig 6. (ZIP)

S7 Data Raw images for Fig 7. (ZIP)

S8 Data Raw images for Fig 9. (ZIP)
