# Supplementary figures and images for: Prefrontal cortex-dependent innate behaviors are altered by selective knockdown of Gad1 in neuropeptide Y interneurons
Source: PLoS One. 2018 Jul 19;13(7):e0200809. doi: 10.1371/journal.pone.0200809 (PMC6053188; doi:10.1371/journal.pone.0200809)

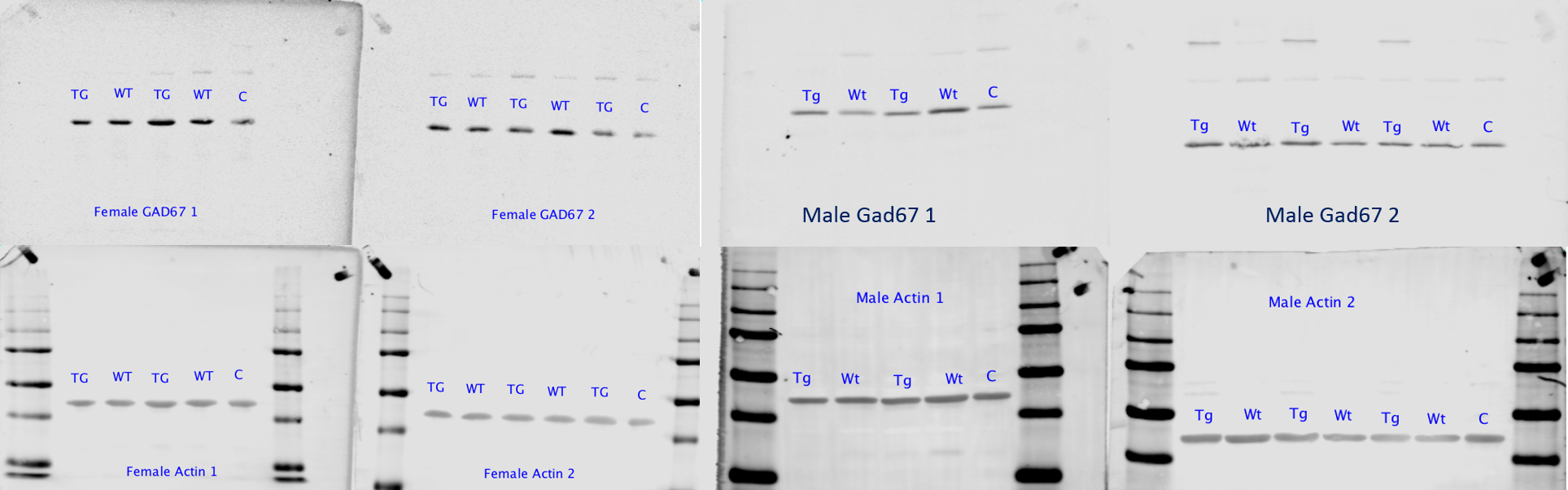

Supplement: S1 Data — (TIF) [file pone.0200809.s001.tif]

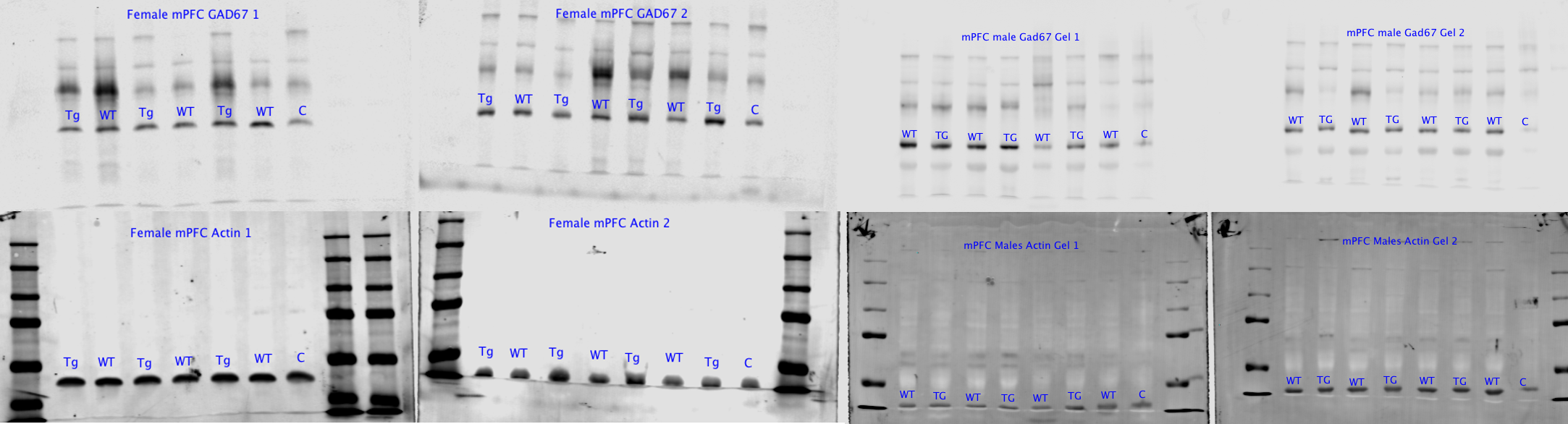

Supplement: S2 Data — (TIF) [file pone.0200809.s002.tif]

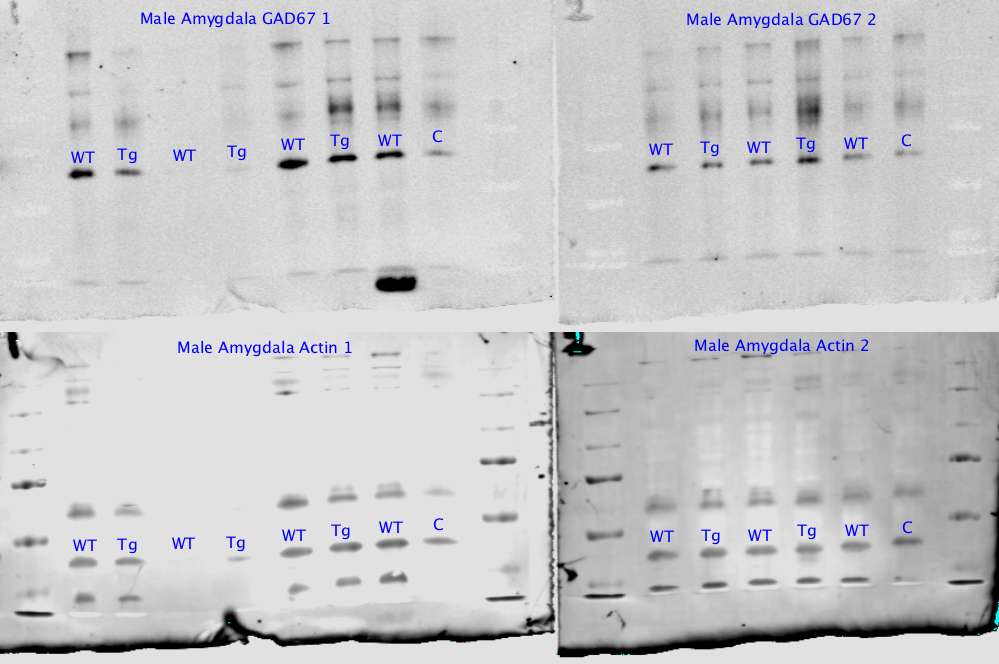

Supplement: S3 Data — (TIF) [file pone.0200809.s003.tif]

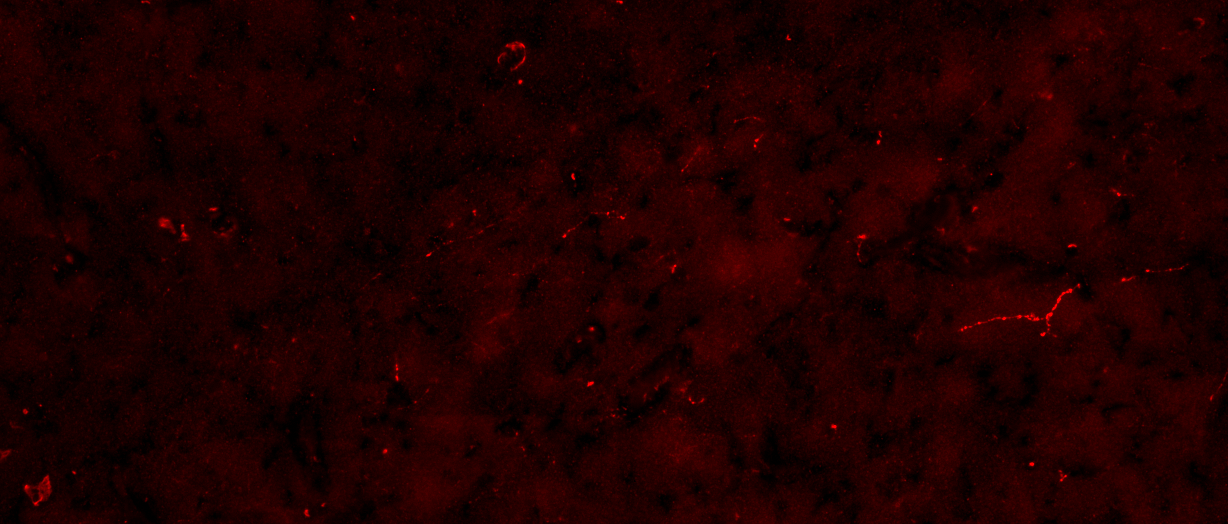

Supplement: S6 Data — (ZIP) [file pone.0200809.s006.zip › S6 Data/TG NPYGFP CA1/RED ROI NPY TG CA1/RED ROI TG17 NPYGFP CA1001.nd2 - C=2.png]

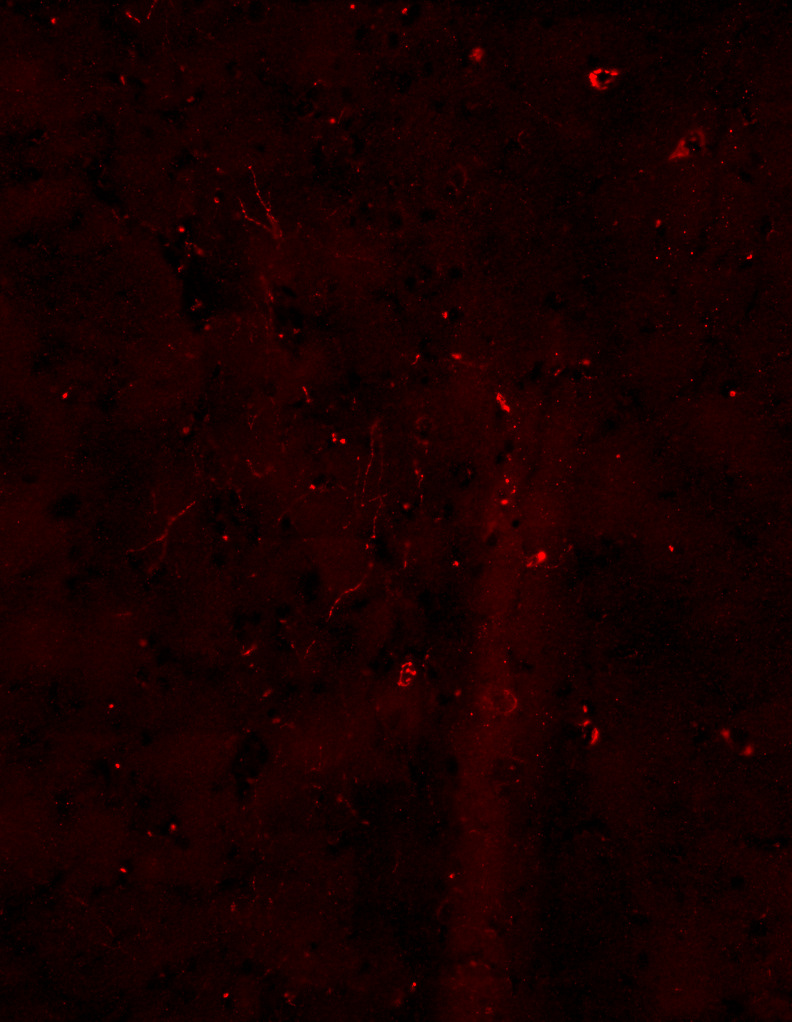

Supplement: S6 Data — (ZIP) [file pone.0200809.s006.zip › S6 Data/TG NPYGFP CA1/RED ROI NPY TG CA1/RED ROI TG19 NPYGFP CA1.nd2 - C=2.png]

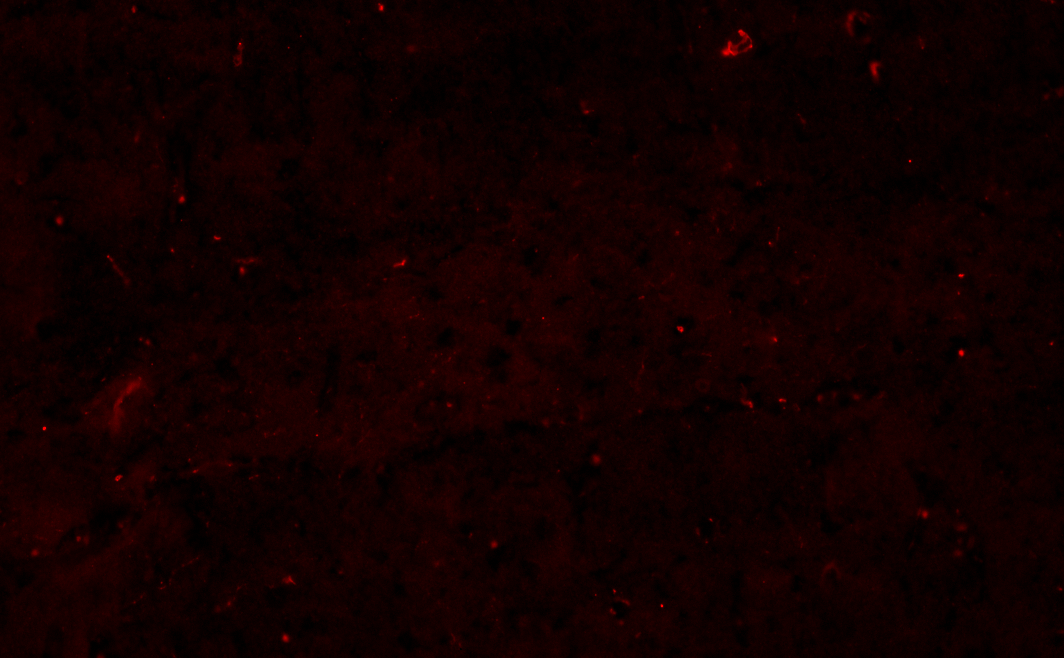

Supplement: S6 Data — (ZIP) [file pone.0200809.s006.zip › S6 Data/TG NPYGFP CA1/RED ROI NPY TG CA1/RED ROI TG24 NPYGFP CA1005.nd2 - C=2.png]

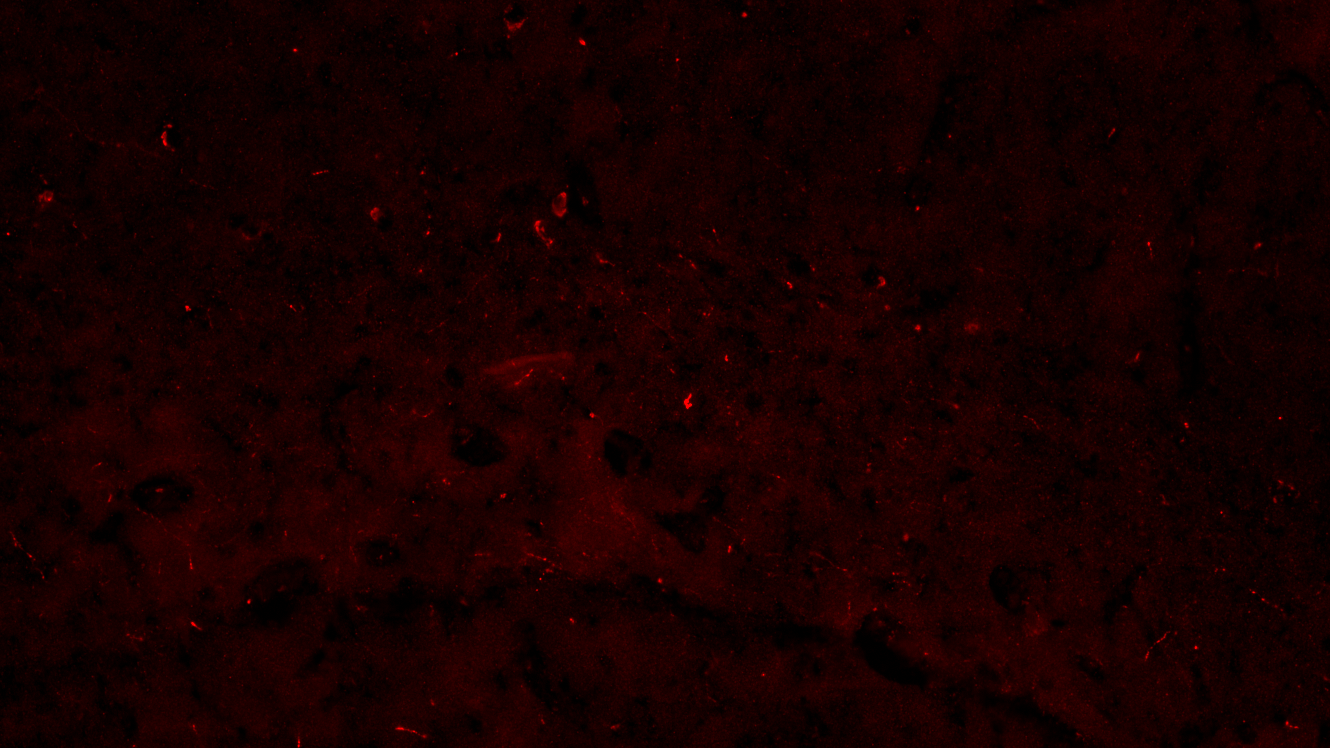

Supplement: S6 Data — (ZIP) [file pone.0200809.s006.zip › S6 Data/TG NPYGFP CA1/RED ROI NPY TG CA1/RED ROI TG310 NPYGFP CA1007.nd2 - C=2.png]

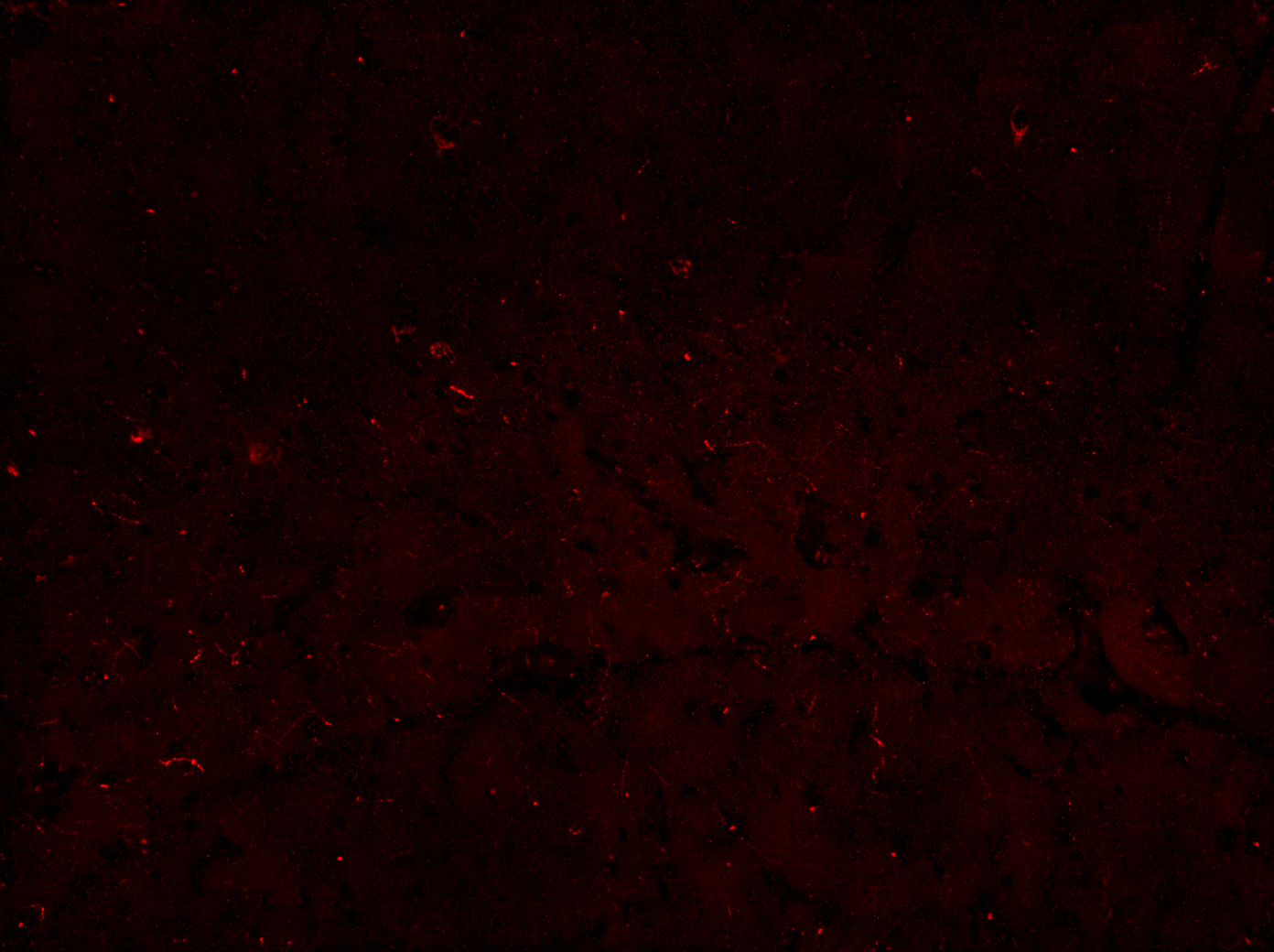

Supplement: S6 Data — (ZIP) [file pone.0200809.s006.zip › S6 Data/TG NPYGFP CA1/RED ROI NPY TG CA1/RED ROI TG311 NPYGFP CA1008.nd2 - C=2.png]

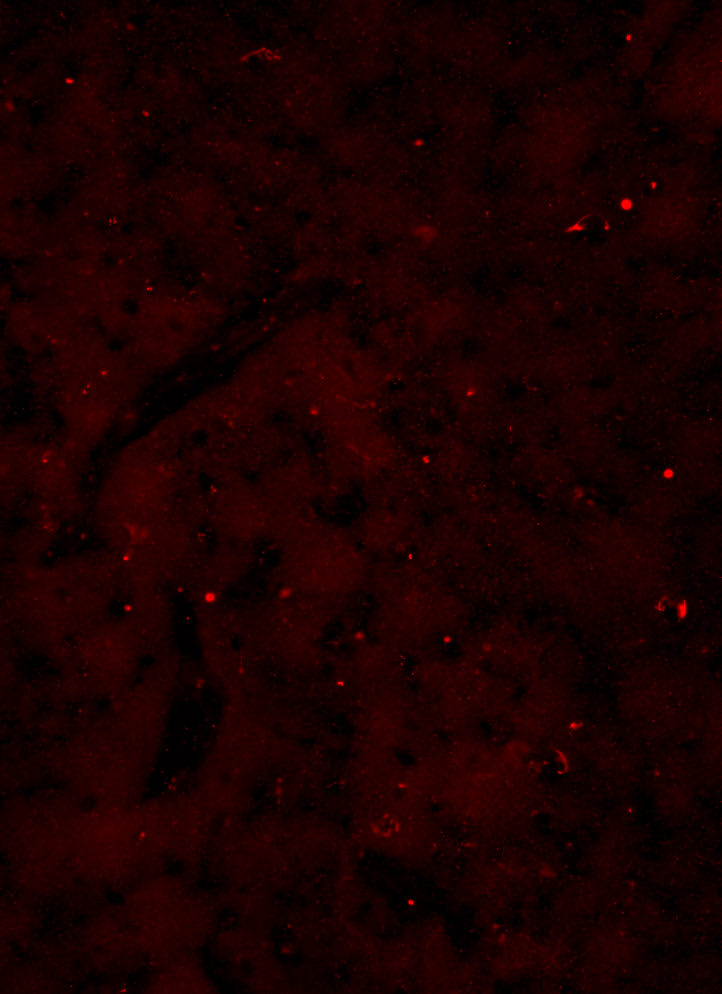

Supplement: S6 Data — (ZIP) [file pone.0200809.s006.zip › S6 Data/TG NPYGFP CA1/RED ROI NPY TG CA1/REDROI TG110 NPYGFP CA1002.nd2 - C=2.png]

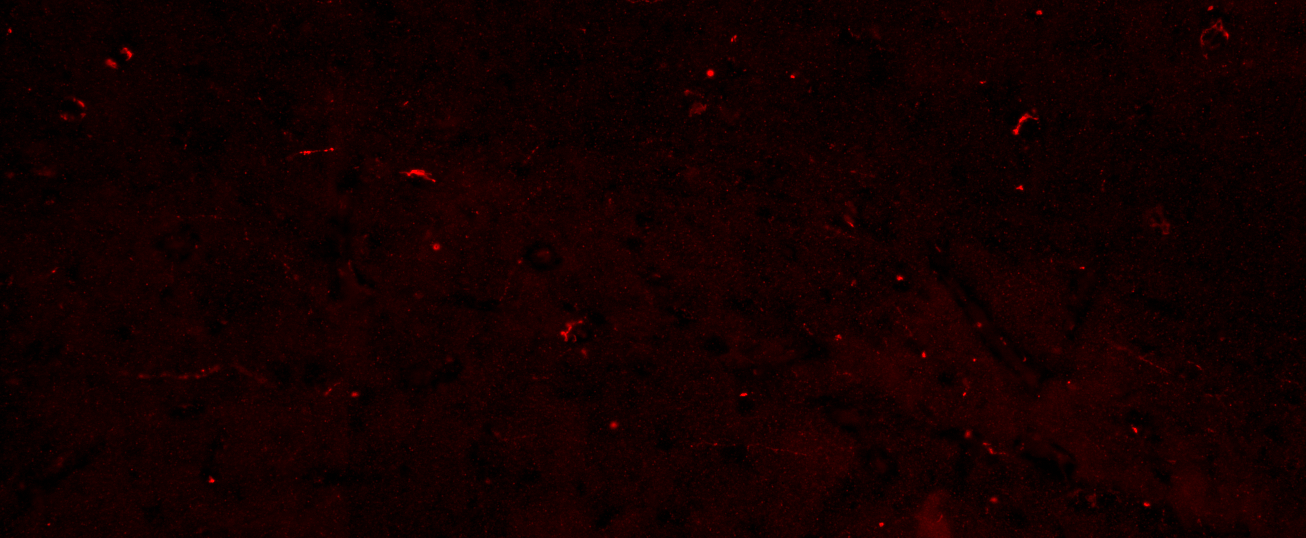

Supplement: S6 Data — (ZIP) [file pone.0200809.s006.zip › S6 Data/TG NPYGFP CA1/RED ROI NPY TG CA1/REDROI TG23 NPYGFP CA1003.nd2 - C=2.png]

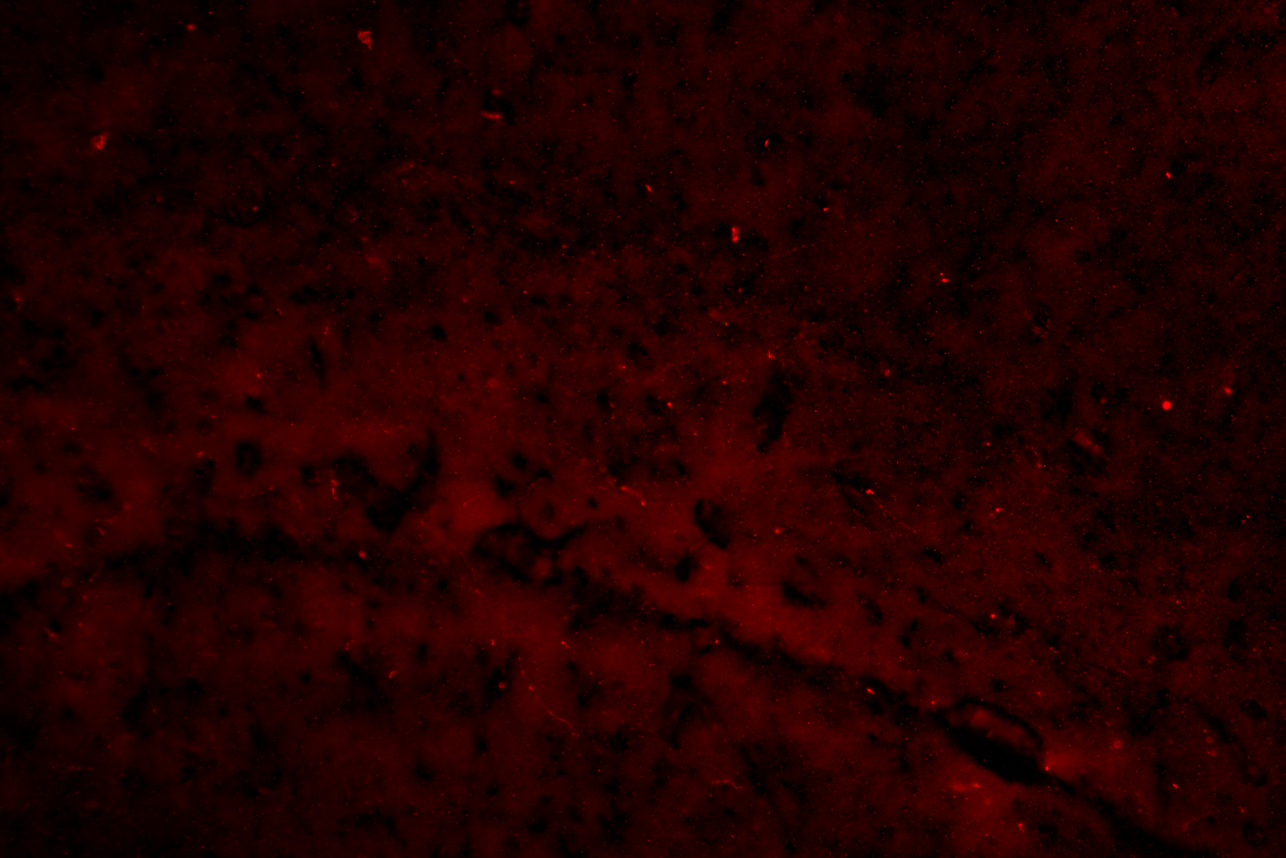

Supplement: S6 Data — (ZIP) [file pone.0200809.s006.zip › S6 Data/TG NPYGFP CA1/RED ROI NPY TG CA1/REDROI TG38 NPYGFP CA1006.nd2 - C=2.png]

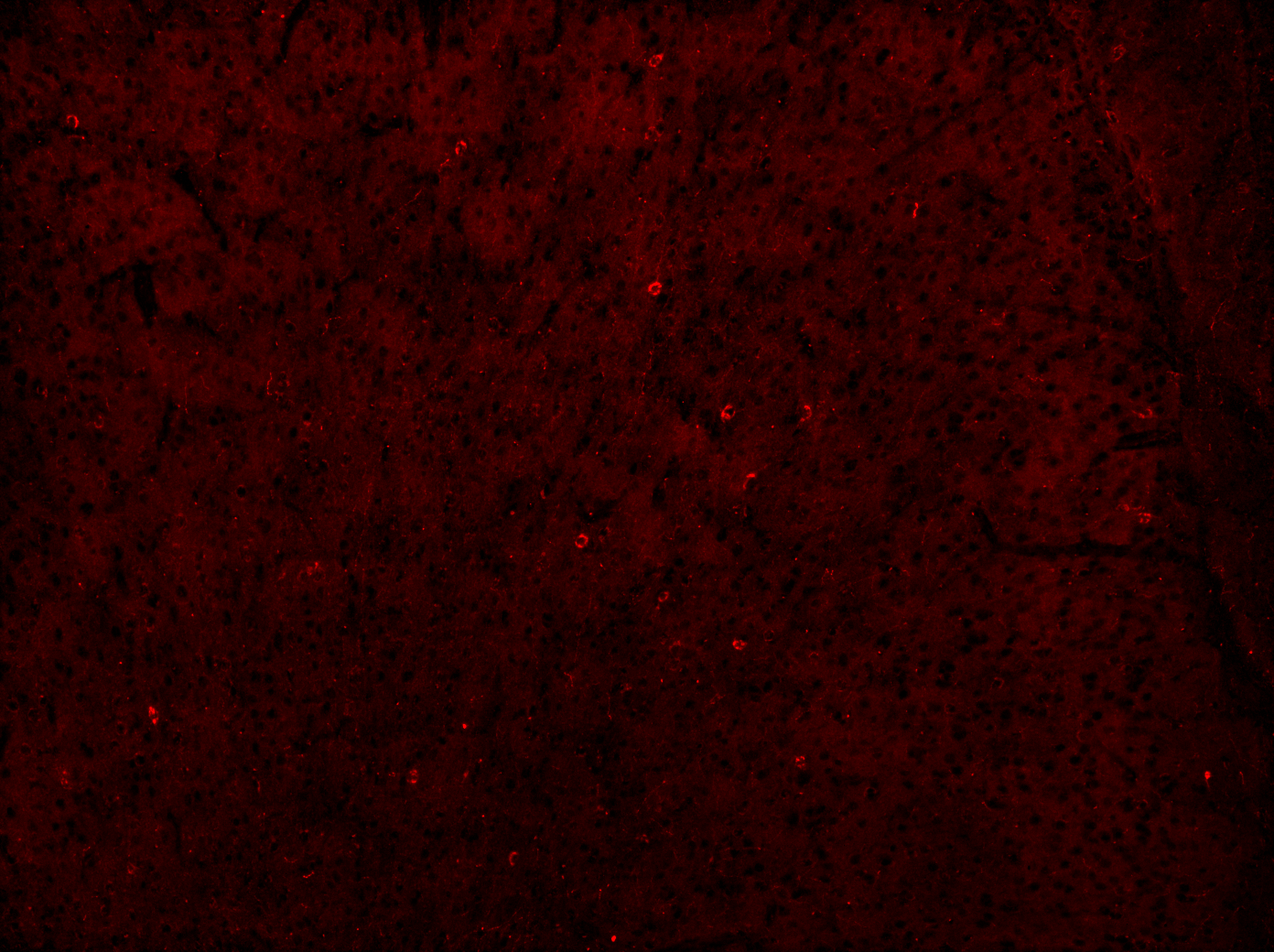

Supplement: S6 Data — (ZIP) [file pone.0200809.s006.zip › S6 Data/TG NPYGFP PFC/RED ROI NPY IMAGES/RED ROI TG12 NPYGFP PFC.nd2 - C=2.png]

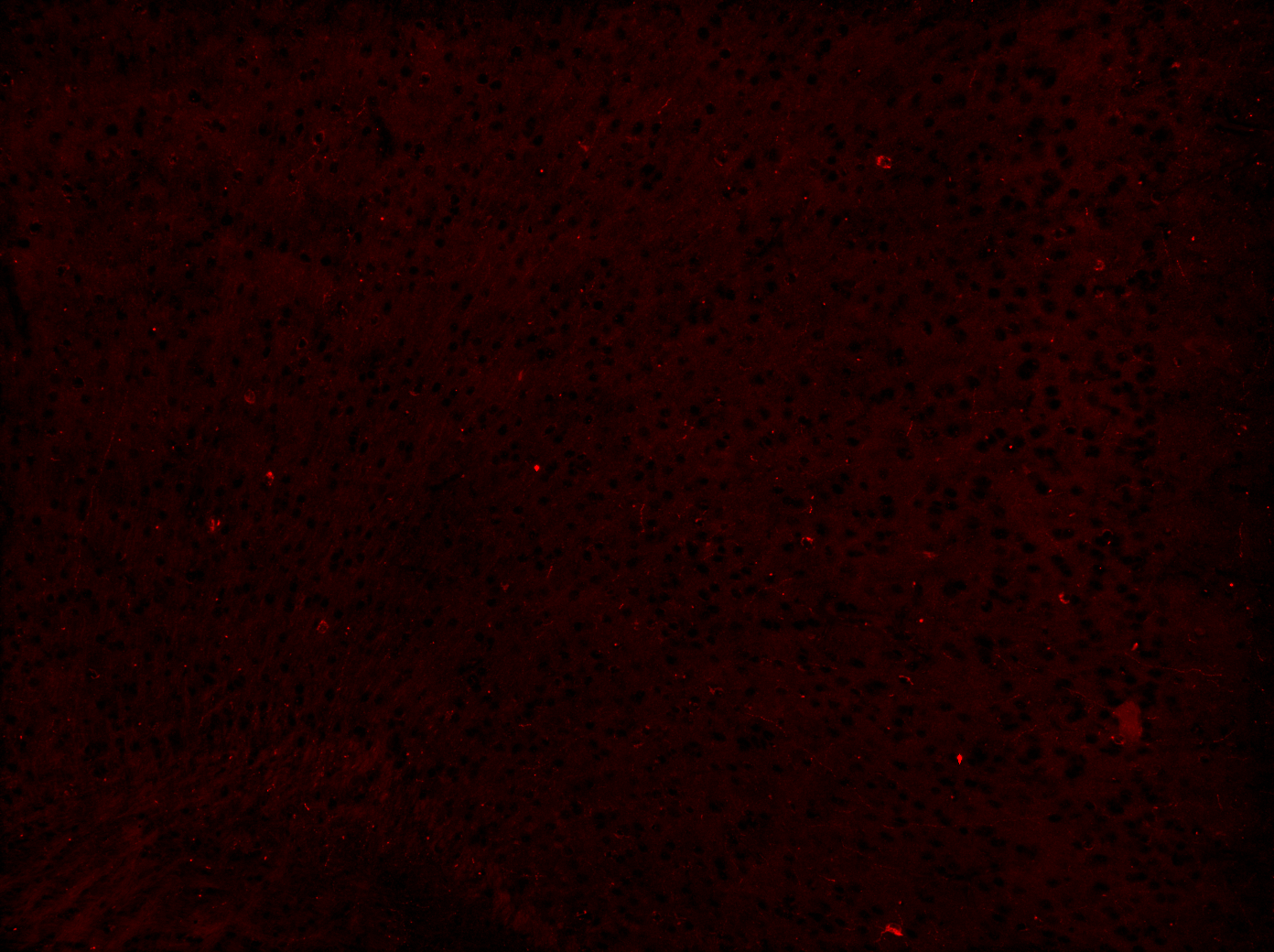

Supplement: S6 Data — (ZIP) [file pone.0200809.s006.zip › S6 Data/TG NPYGFP PFC/RED ROI NPY IMAGES/RED ROI TG21 NPYGFP PFC003.nd2 - C=2.png]

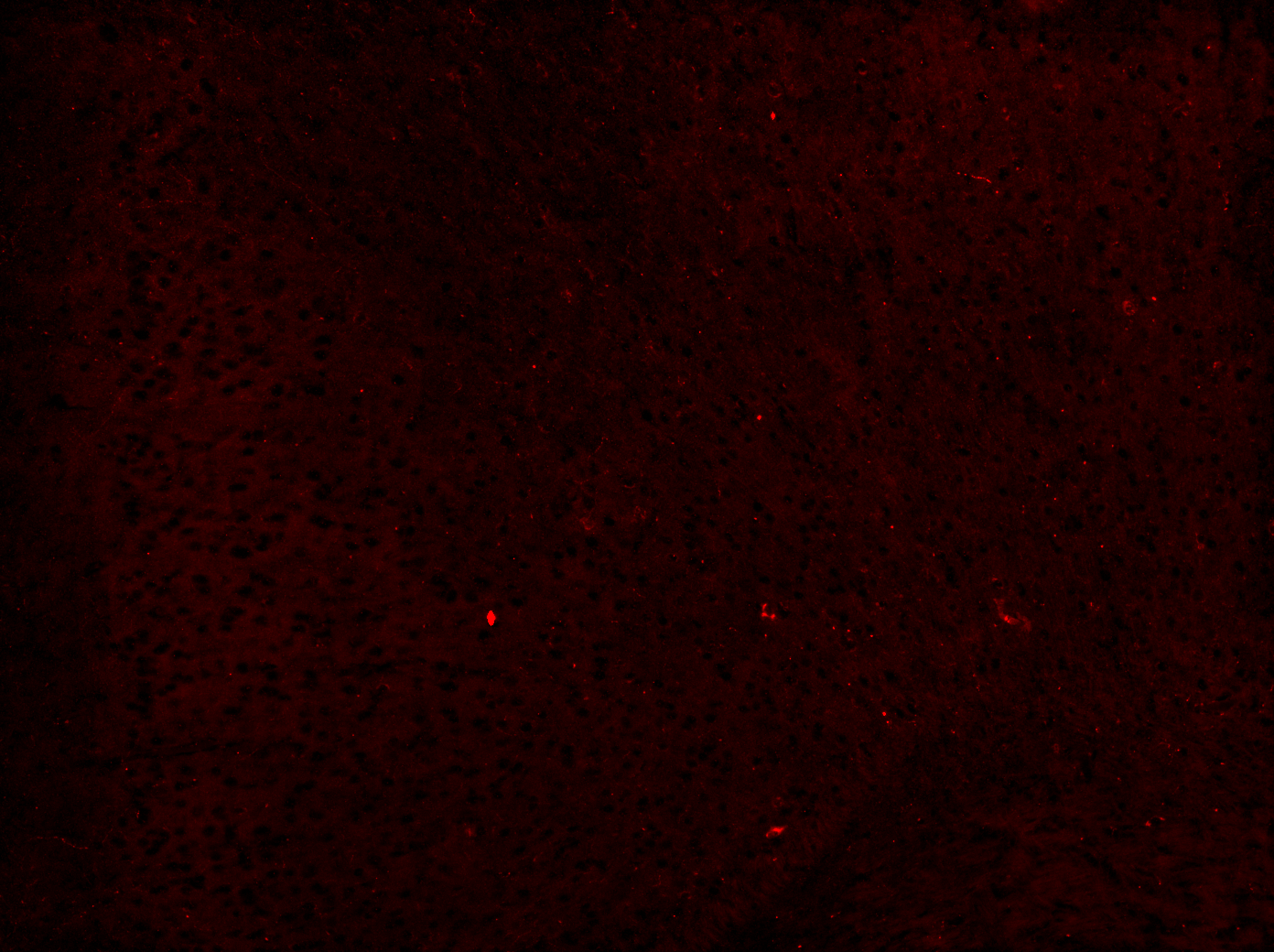

Supplement: S6 Data — (ZIP) [file pone.0200809.s006.zip › S6 Data/TG NPYGFP PFC/RED ROI NPY IMAGES/RED ROI TG22 NPYGFP PFC004.nd2 - C=2.png]

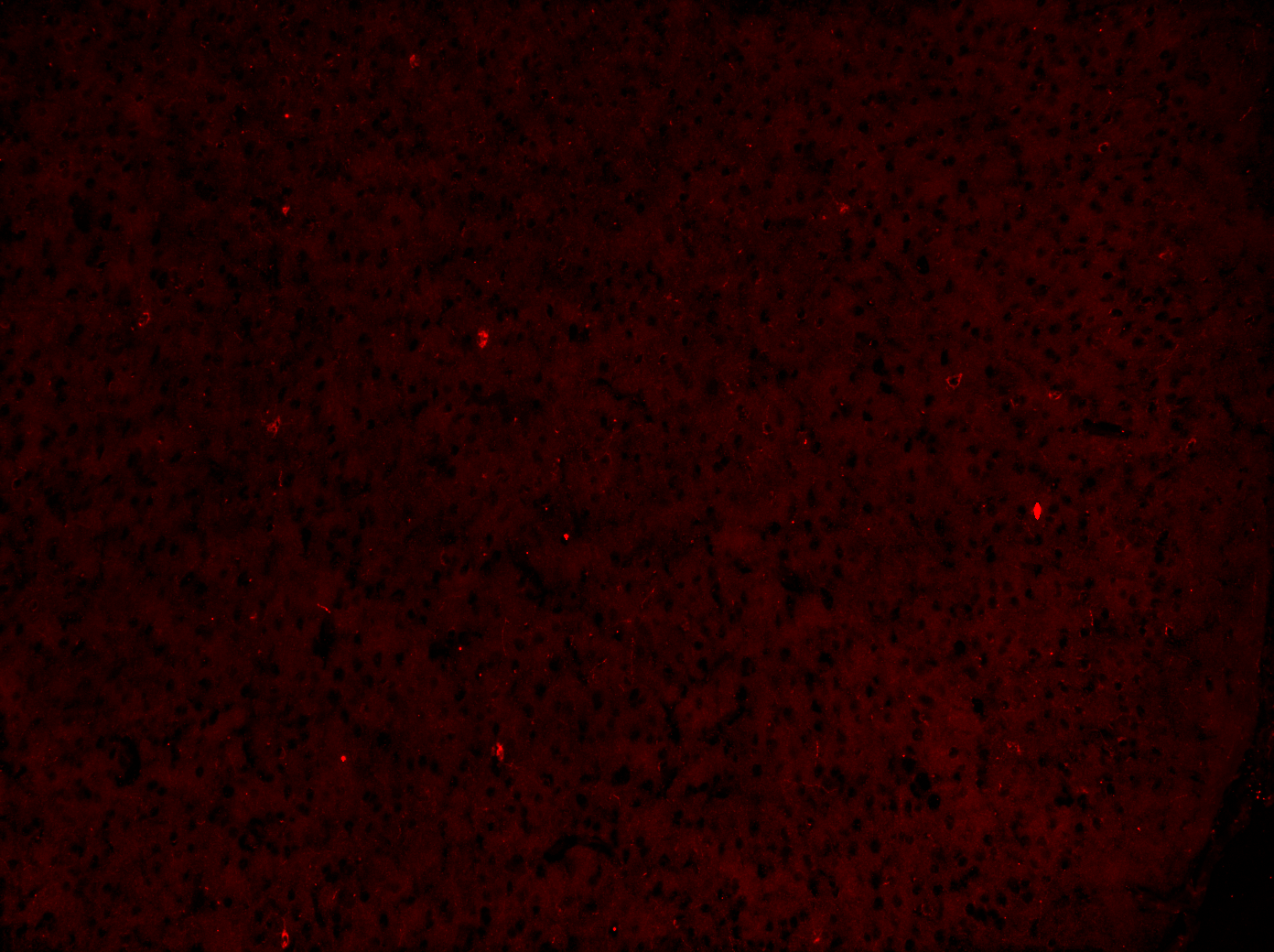

Supplement: S6 Data — (ZIP) [file pone.0200809.s006.zip › S6 Data/TG NPYGFP PFC/RED ROI NPY IMAGES/RED ROI TG33 NPYGFP PFC002.nd2 - C=2.png]

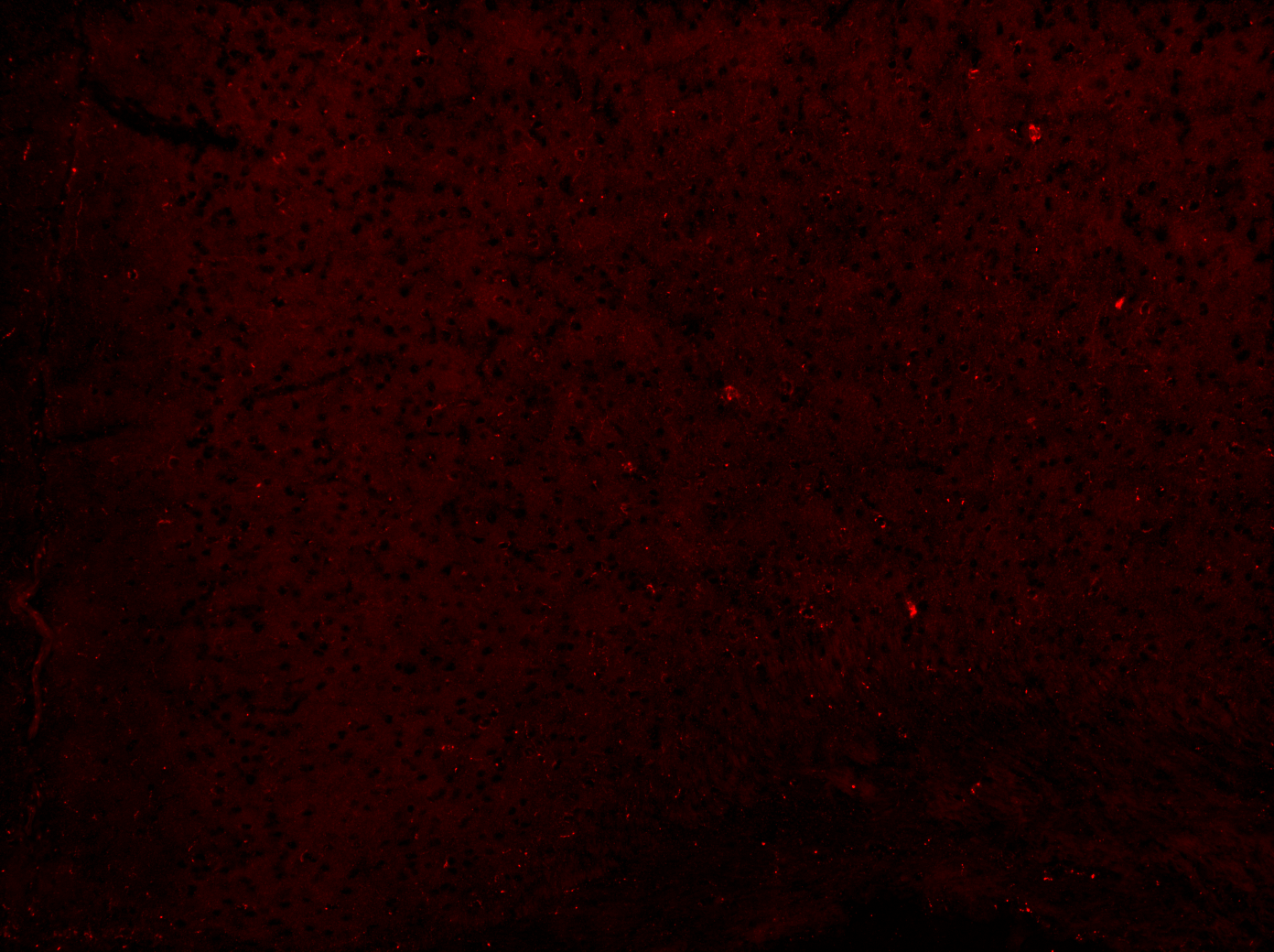

Supplement: S6 Data — (ZIP) [file pone.0200809.s006.zip › S6 Data/TG NPYGFP PFC/RED ROI NPY IMAGES/REDROI TG31 NPYGFP PFC.nd2 - C=2.png]

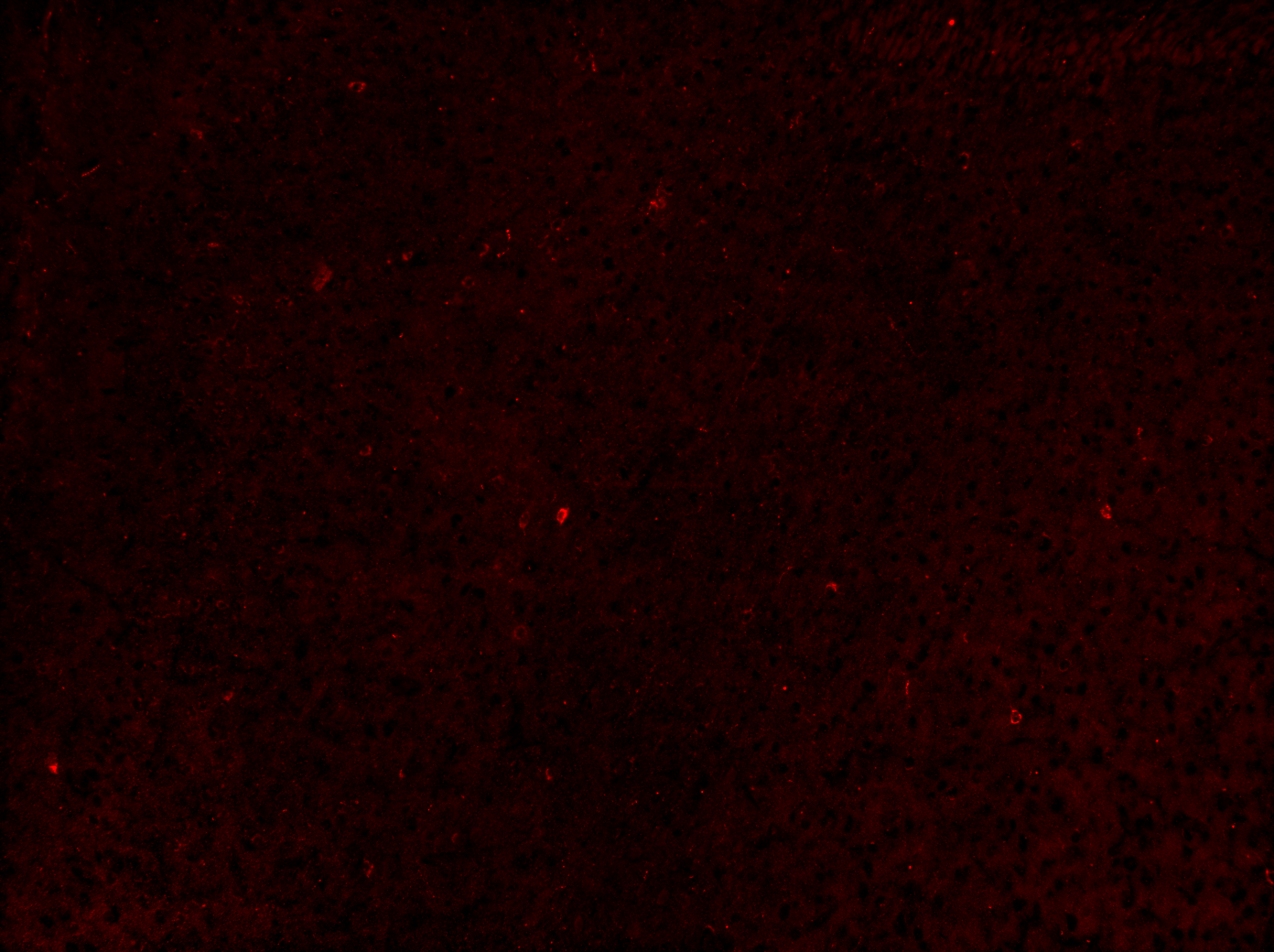

Supplement: S6 Data — (ZIP) [file pone.0200809.s006.zip › S6 Data/TG NPYGFP PFC/RED ROI NPY IMAGES/REDROI TG32 NPYGFP PFC001.nd2 - C=2.png]

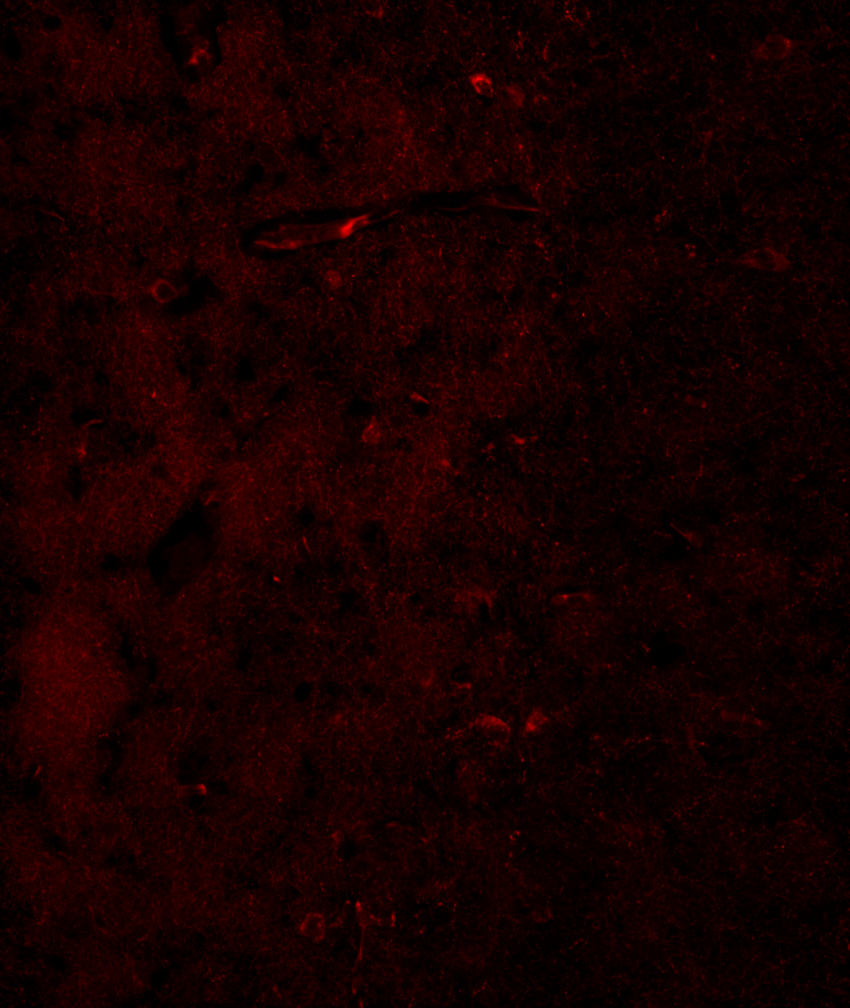

Supplement: S7 Data — (ZIP) [file pone.0200809.s007.zip › S7 Data/TG CA1 NPYGAD67/tg ca1 npygad67/ROI gad67 images/red roi gad67 images from gfpgad67coloc/mouse 1/REDROI tg110 ca1 gad67gfp.nd2 - C=2.png]

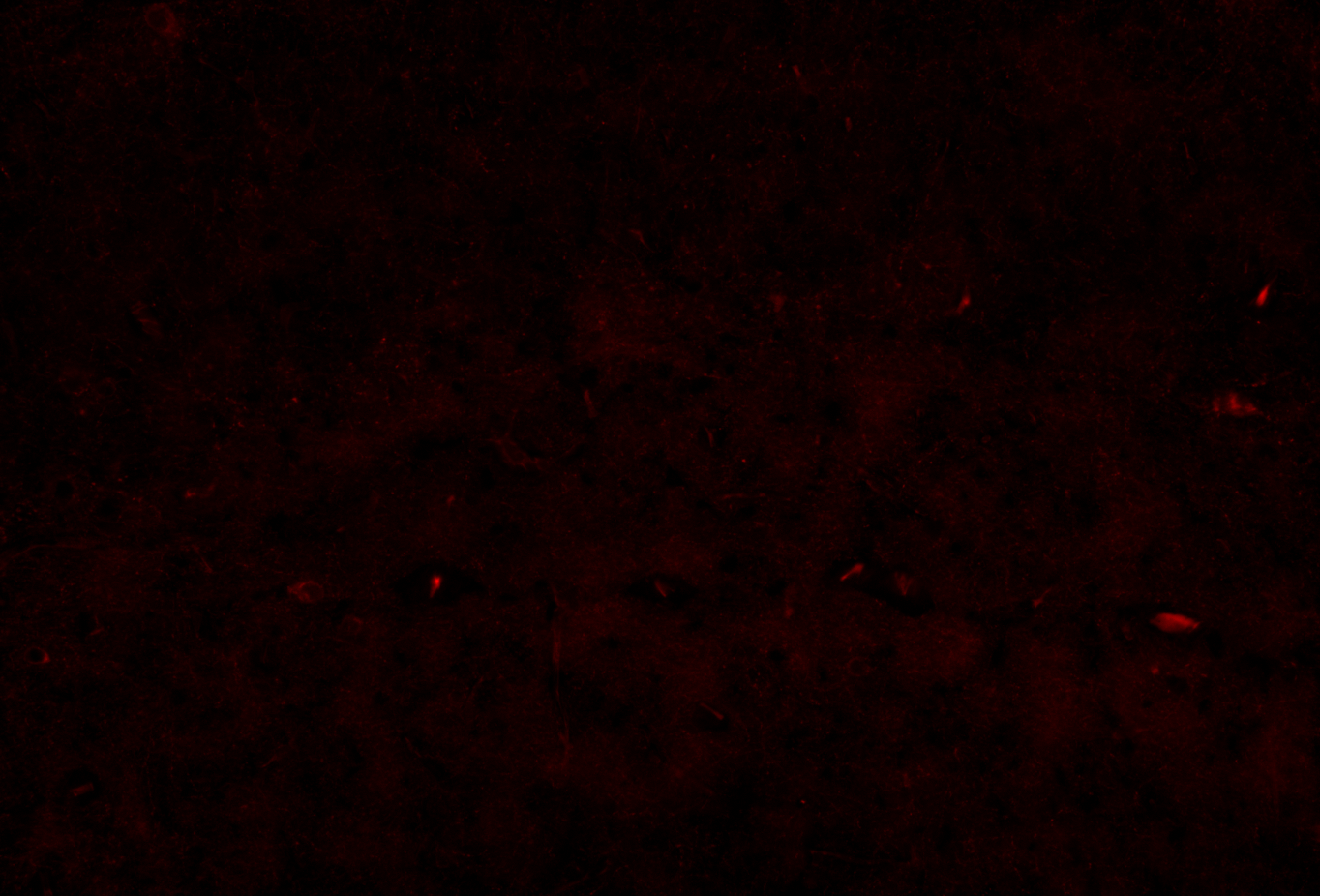

Supplement: S7 Data — (ZIP) [file pone.0200809.s007.zip › S7 Data/TG CA1 NPYGAD67/tg ca1 npygad67/ROI gad67 images/red roi gad67 images from gfpgad67coloc/mouse 1/REDROI tg17 ca1 gad67gfp.nd2 - C=2.png]

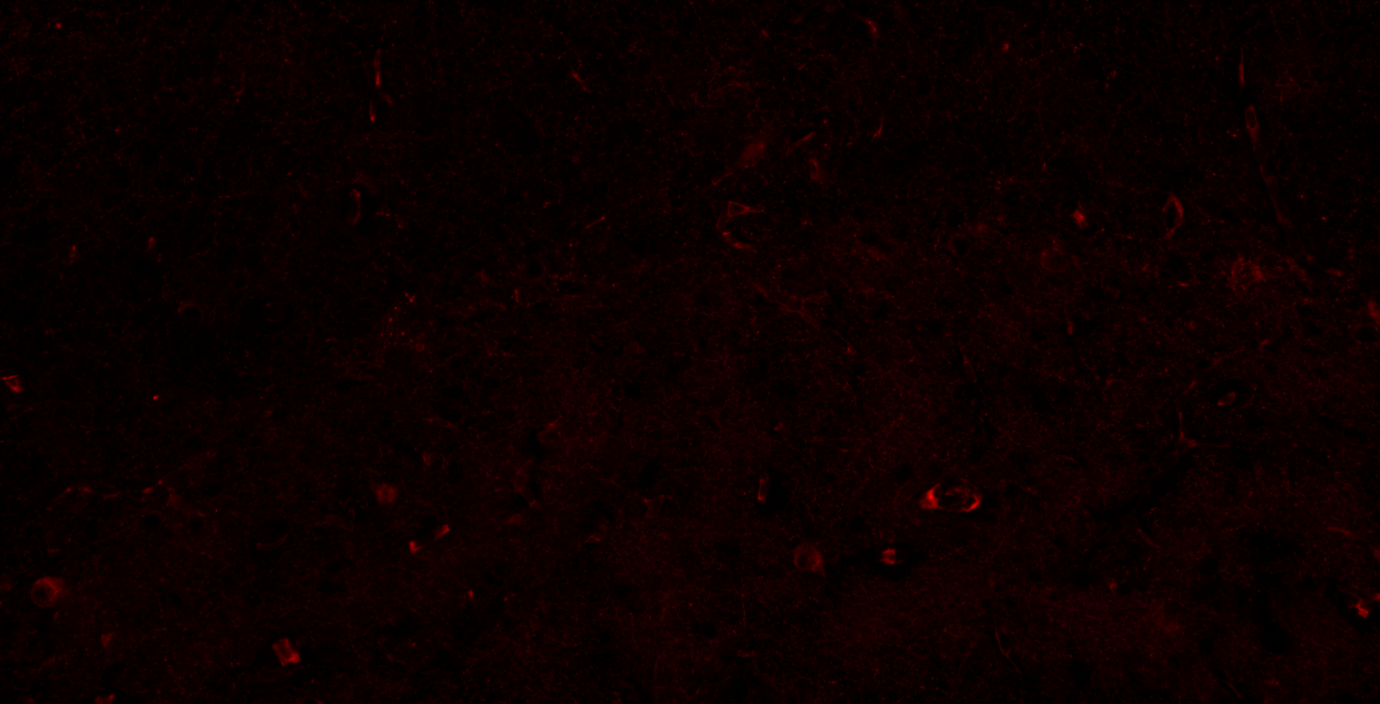

Supplement: S7 Data — (ZIP) [file pone.0200809.s007.zip › S7 Data/TG CA1 NPYGAD67/tg ca1 npygad67/ROI gad67 images/red roi gad67 images from gfpgad67coloc/mouse 1/REDROI tg19 ca1 gad67gfp001.nd2 - C=2.png]

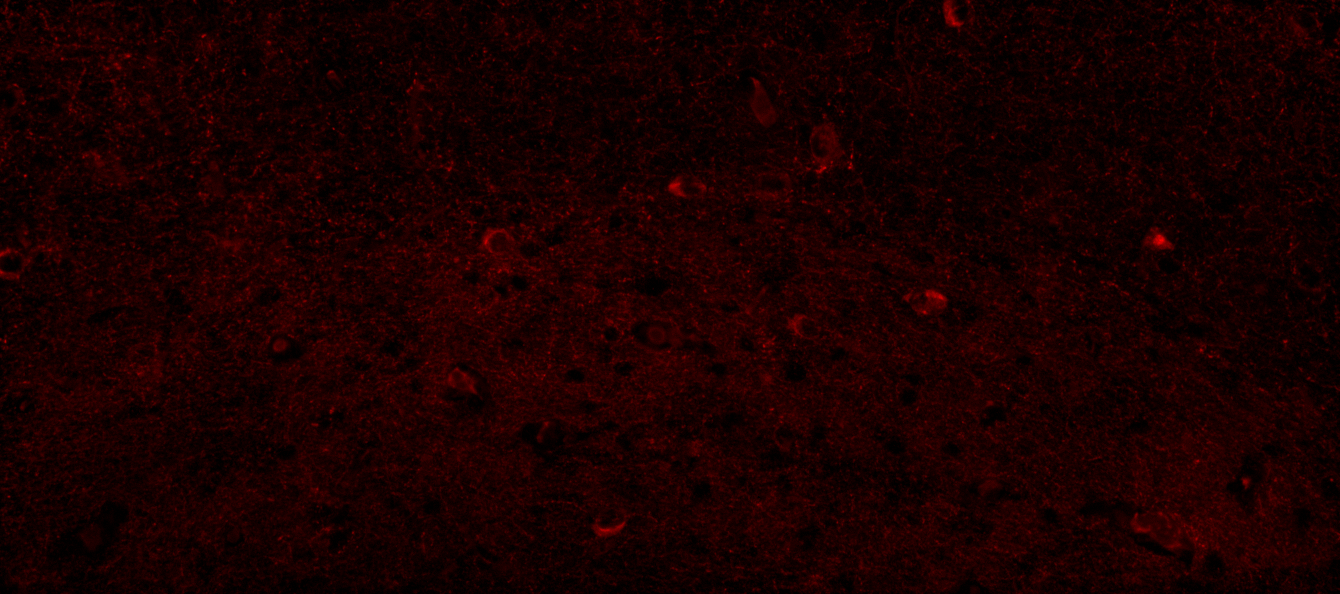

Supplement: S7 Data — (ZIP) [file pone.0200809.s007.zip › S7 Data/TG CA1 NPYGAD67/tg ca1 npygad67/ROI gad67 images/red roi gad67 images from gfpgad67coloc/mouse 2/redroi tg24 ca1 gad67gfp.nd2 - C=2.png]

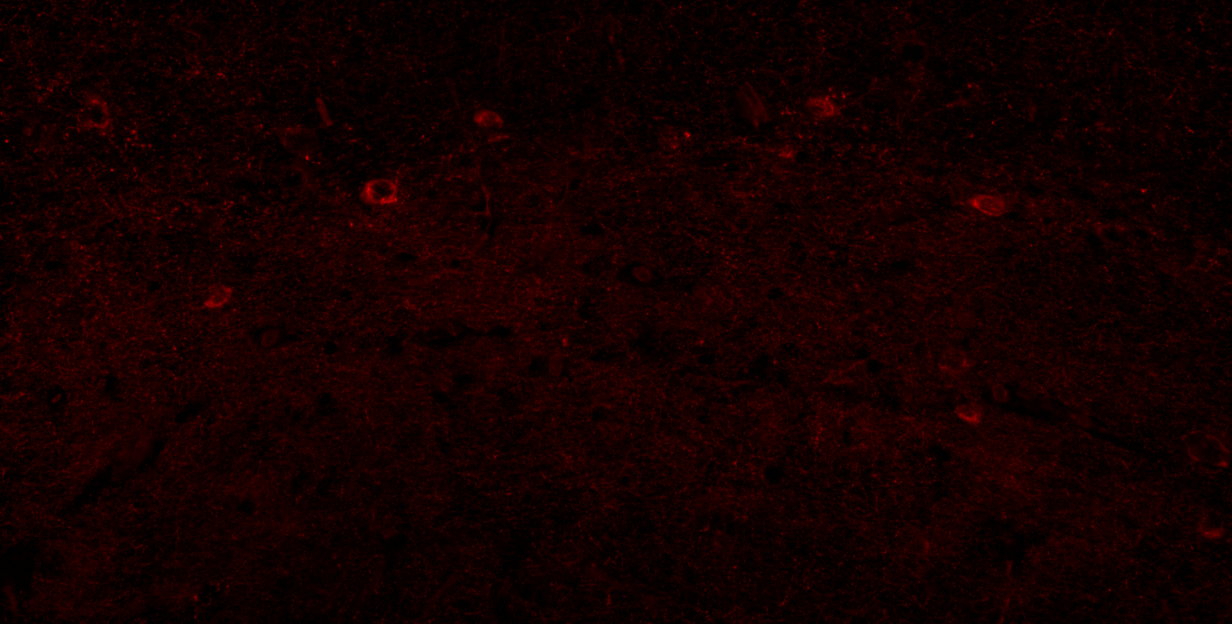

Supplement: S7 Data — (ZIP) [file pone.0200809.s007.zip › S7 Data/TG CA1 NPYGAD67/tg ca1 npygad67/ROI gad67 images/red roi gad67 images from gfpgad67coloc/mouse 2/redroi tg25 ca1 gad67gfp001.nd2 - C=2.png]

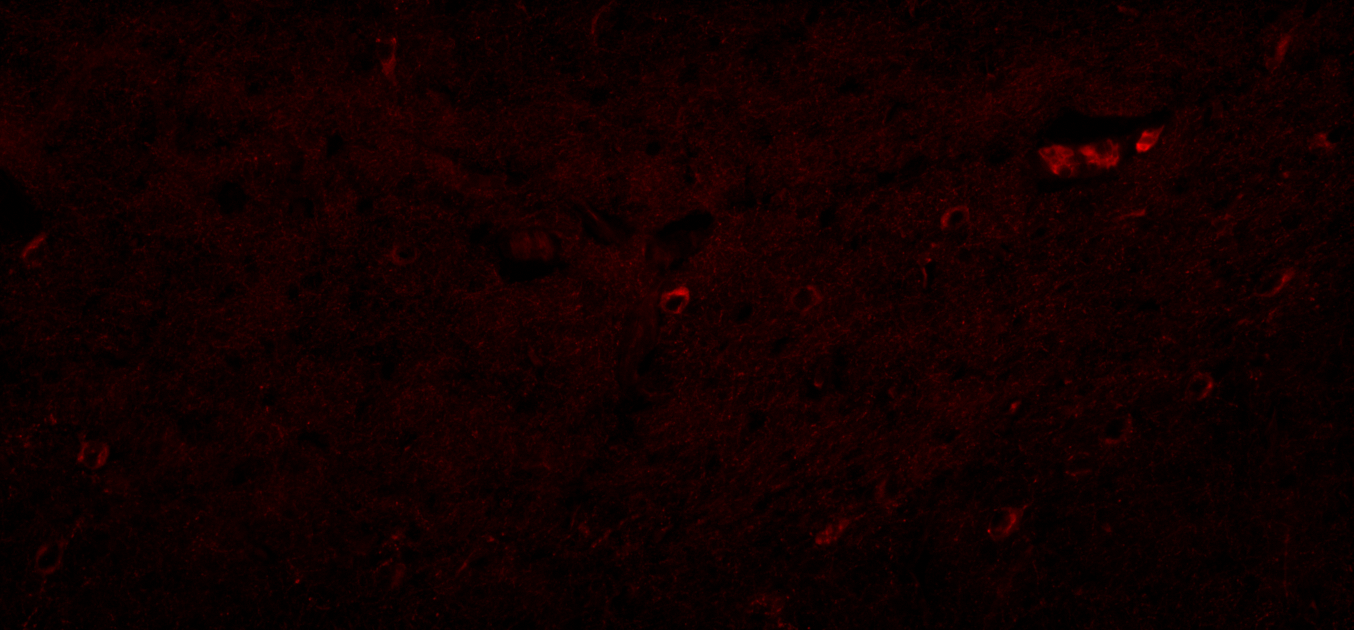

Supplement: S7 Data — (ZIP) [file pone.0200809.s007.zip › S7 Data/TG CA1 NPYGAD67/tg ca1 npygad67/ROI gad67 images/red roi gad67 images from gfpgad67coloc/mouse 3/redroi tg310 ca1 gad67gfp001.nd2 - C=2.png]

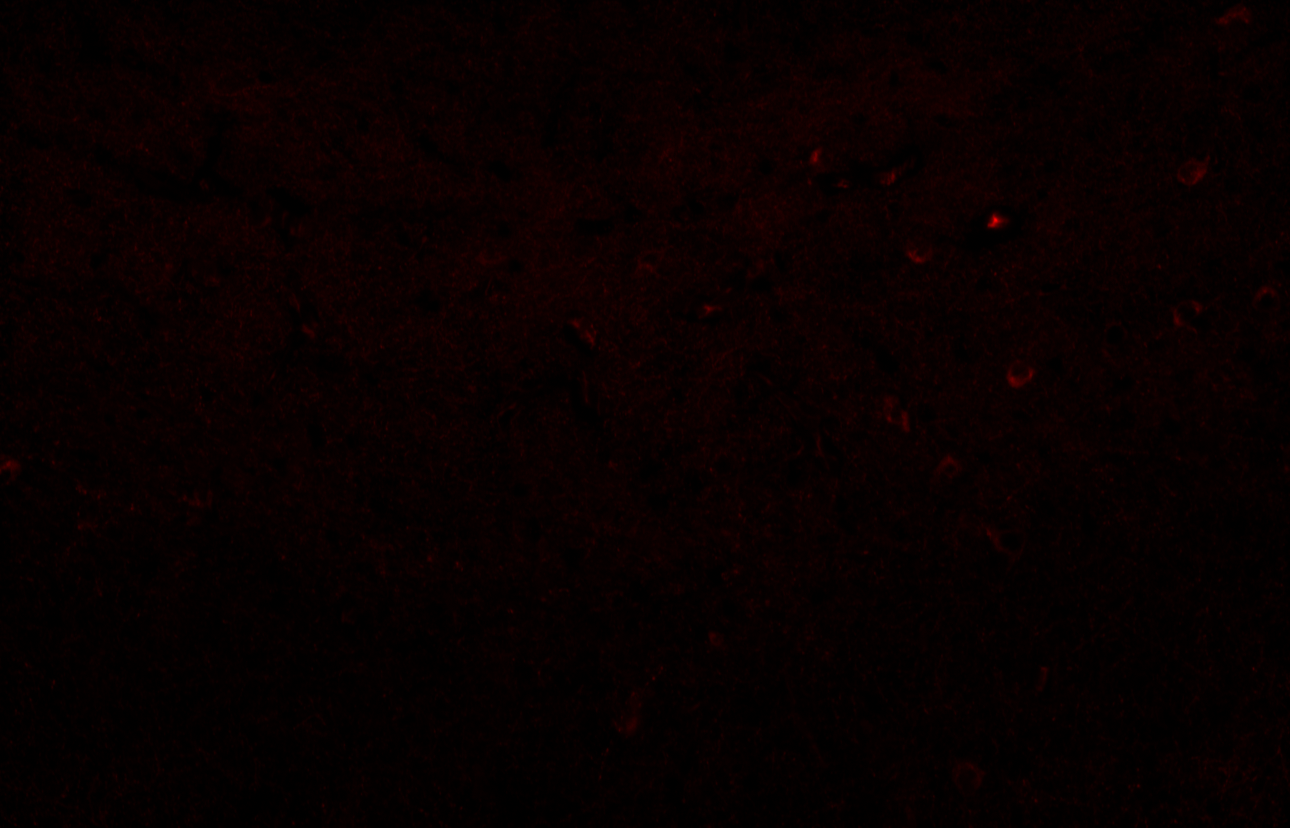

Supplement: S7 Data — (ZIP) [file pone.0200809.s007.zip › S7 Data/TG CA1 NPYGAD67/tg ca1 npygad67/ROI gad67 images/red roi gad67 images from gfpgad67coloc/mouse 3/redroi tg311 ca1 gad67gfp.nd2 - C=2.png]

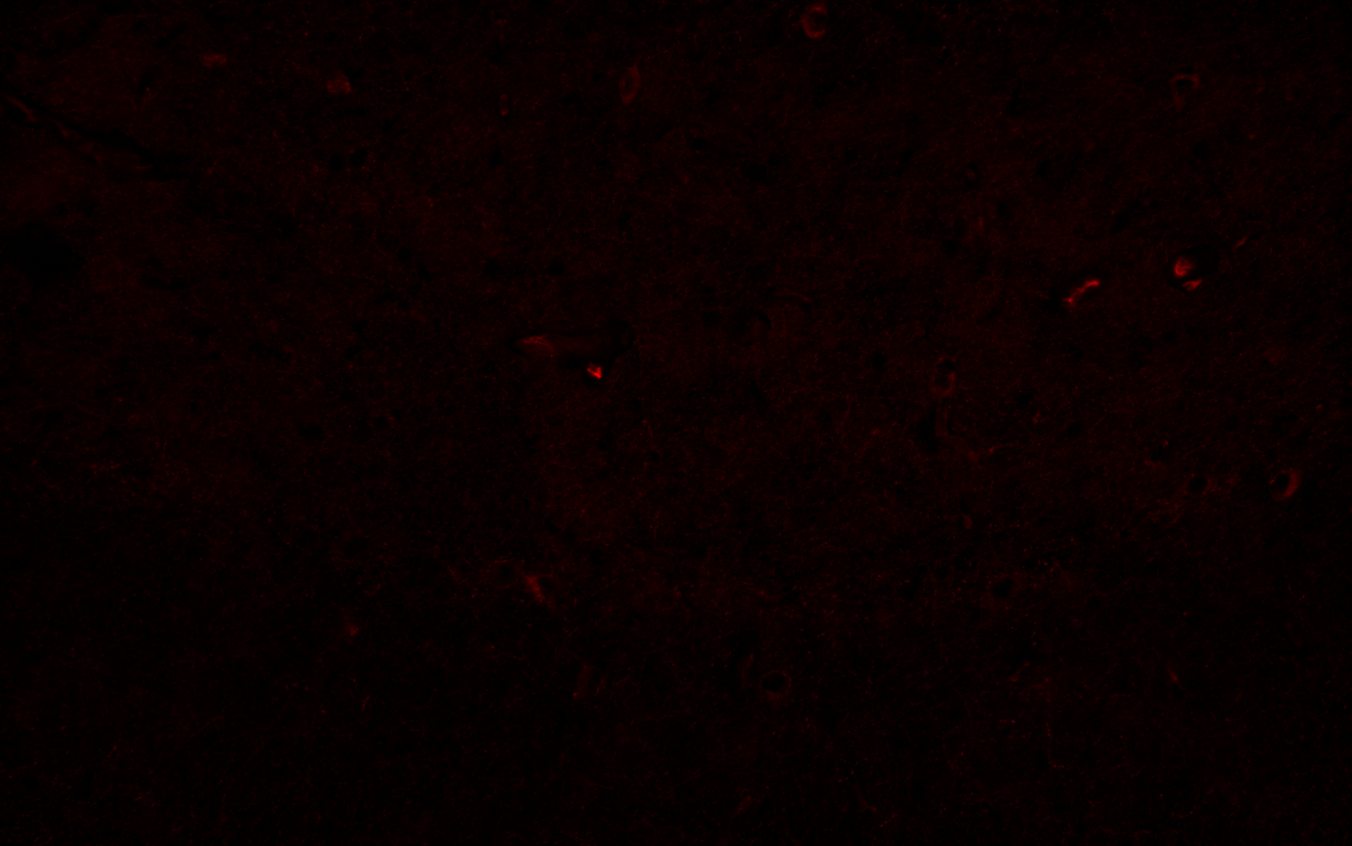

Supplement: S7 Data — (ZIP) [file pone.0200809.s007.zip › S7 Data/TG CA1 NPYGAD67/tg ca1 npygad67/ROI gad67 images/red roi gad67 images from gfpgad67coloc/mouse 3/redroi tg38 ca1 gad67gfp.nd2 - C=2.png]

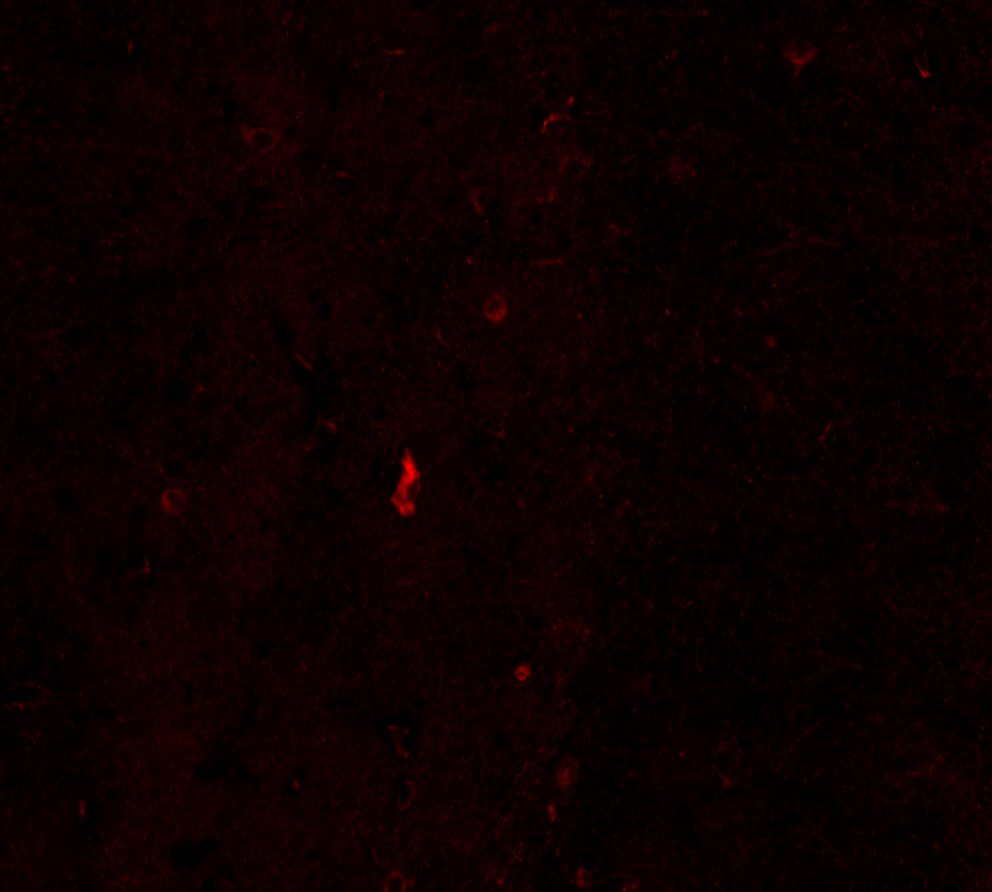

Supplement: S7 Data — (ZIP) [file pone.0200809.s007.zip › S7 Data/TG CA1 NPYGAD67/tg ca1 npygad67/ROI gad67 images/red roi gad67 images from npygad67coloc/mouse 1/redroi tg19 ca1 gad67npy.nd2 - C=2.png]

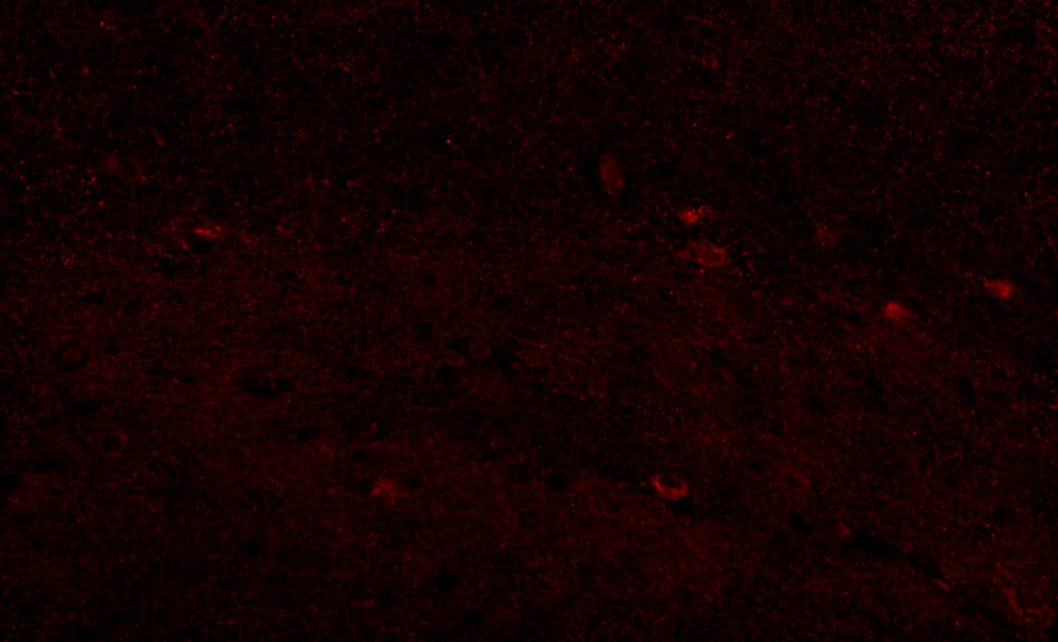

Supplement: S7 Data — (ZIP) [file pone.0200809.s007.zip › S7 Data/TG CA1 NPYGAD67/tg ca1 npygad67/ROI gad67 images/red roi gad67 images from npygad67coloc/mouse 2/redroi tg24 ca1 gad67npy001.nd2 - C=2.png]

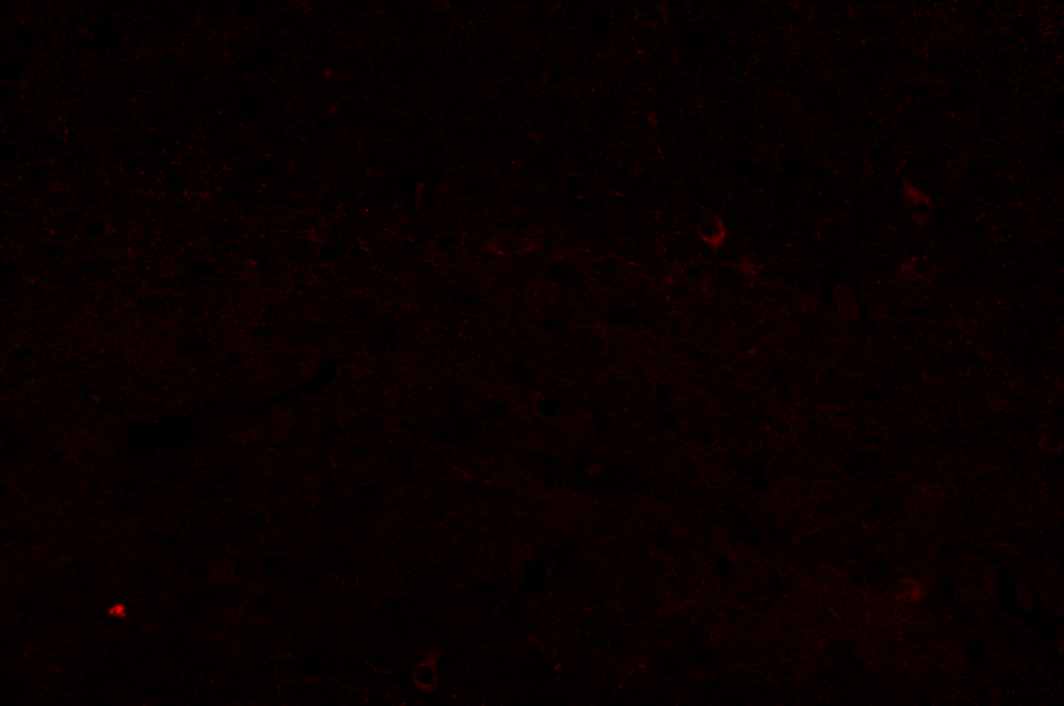

Supplement: S7 Data — (ZIP) [file pone.0200809.s007.zip › S7 Data/TG CA1 NPYGAD67/tg ca1 npygad67/ROI gad67 images/red roi gad67 images from npygad67coloc/mouse 2/redroi tg25 ca1 gad67npy.nd2 - C=2.png]

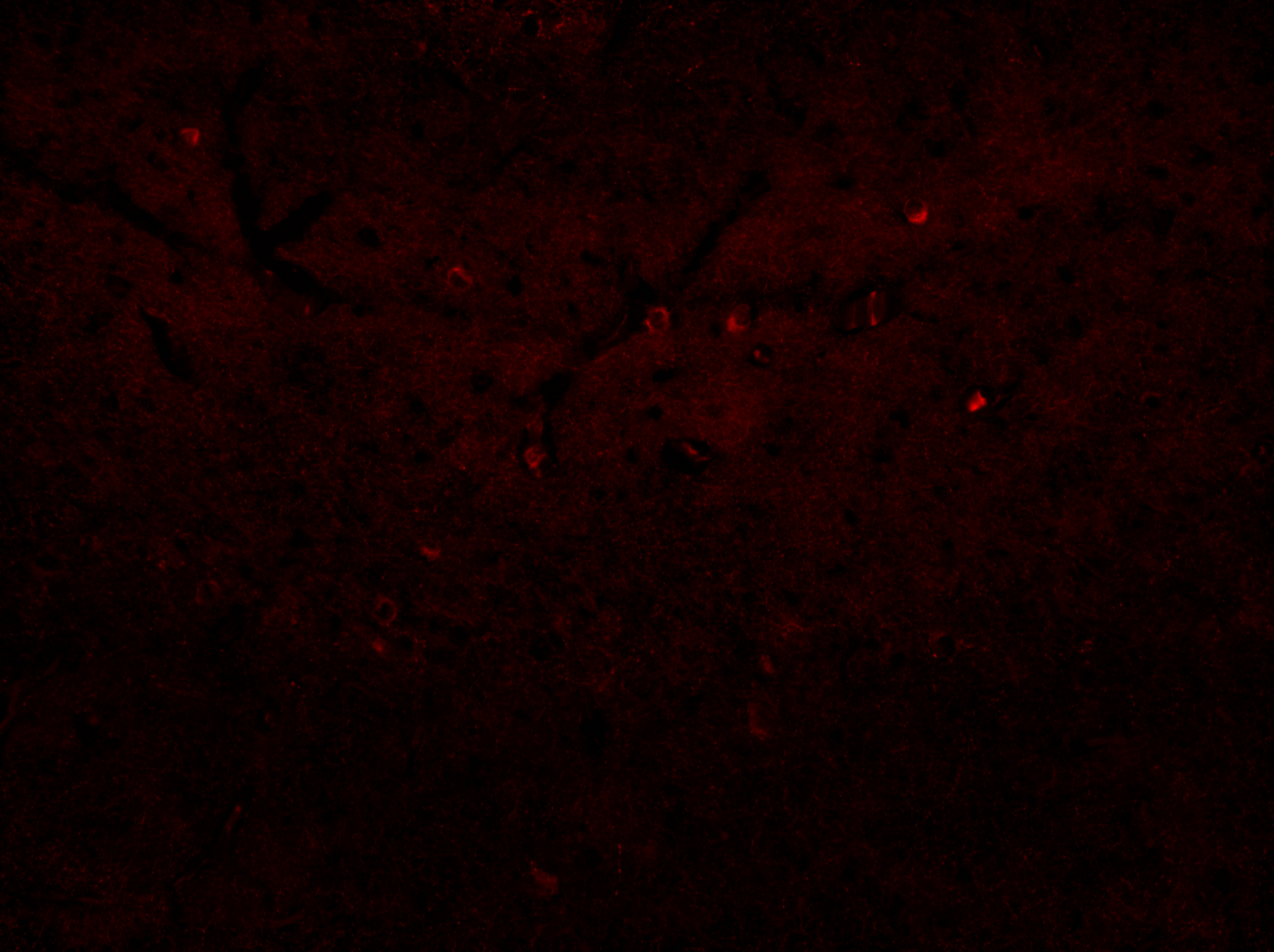

Supplement: S7 Data — (ZIP) [file pone.0200809.s007.zip › S7 Data/TG CA1 NPYGAD67/tg ca1 npygad67/ROI gad67 images/red roi gad67 images from npygad67coloc/mouse 3/REDROI tg310 ca1 gad67npy.nd2 - C=2.png]

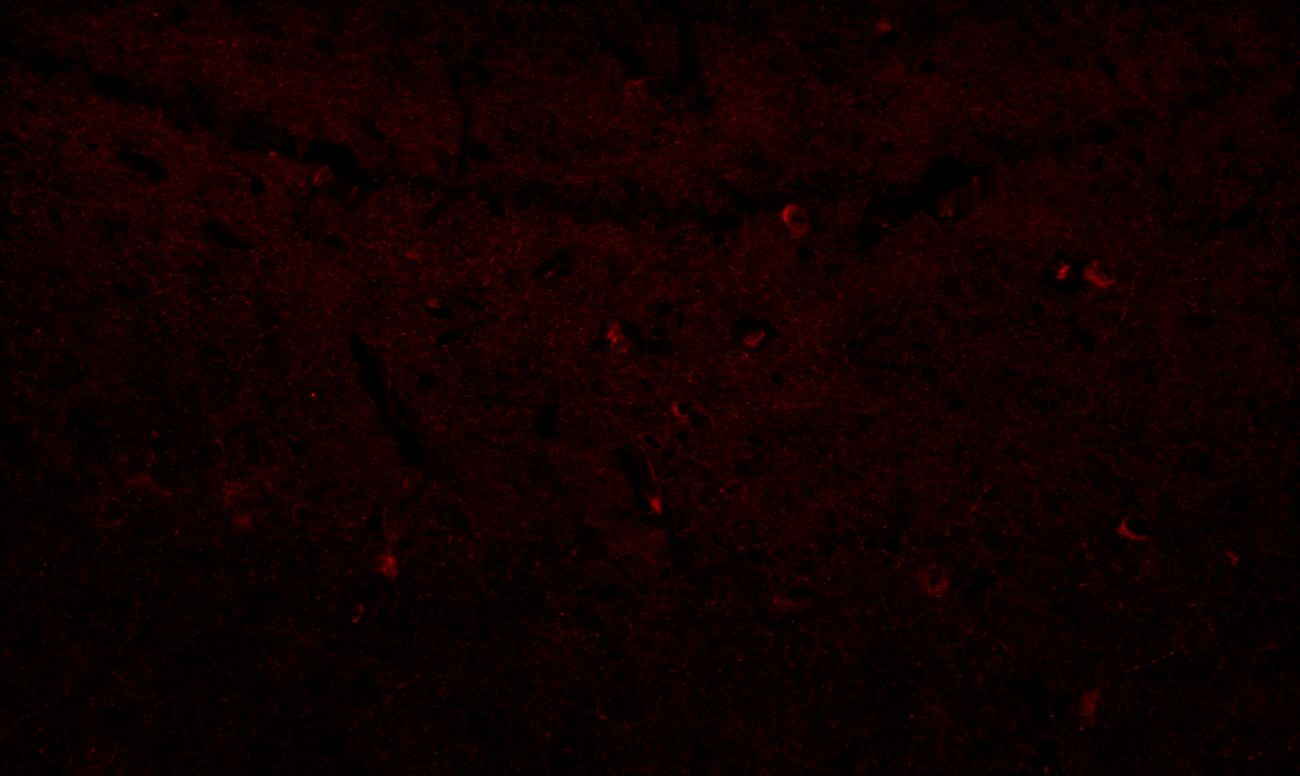

Supplement: S7 Data — (ZIP) [file pone.0200809.s007.zip › S7 Data/TG CA1 NPYGAD67/tg ca1 npygad67/ROI gad67 images/red roi gad67 images from npygad67coloc/mouse 3/REDROI tg311 ca1 gad67npy001.nd2 - C=2.png]

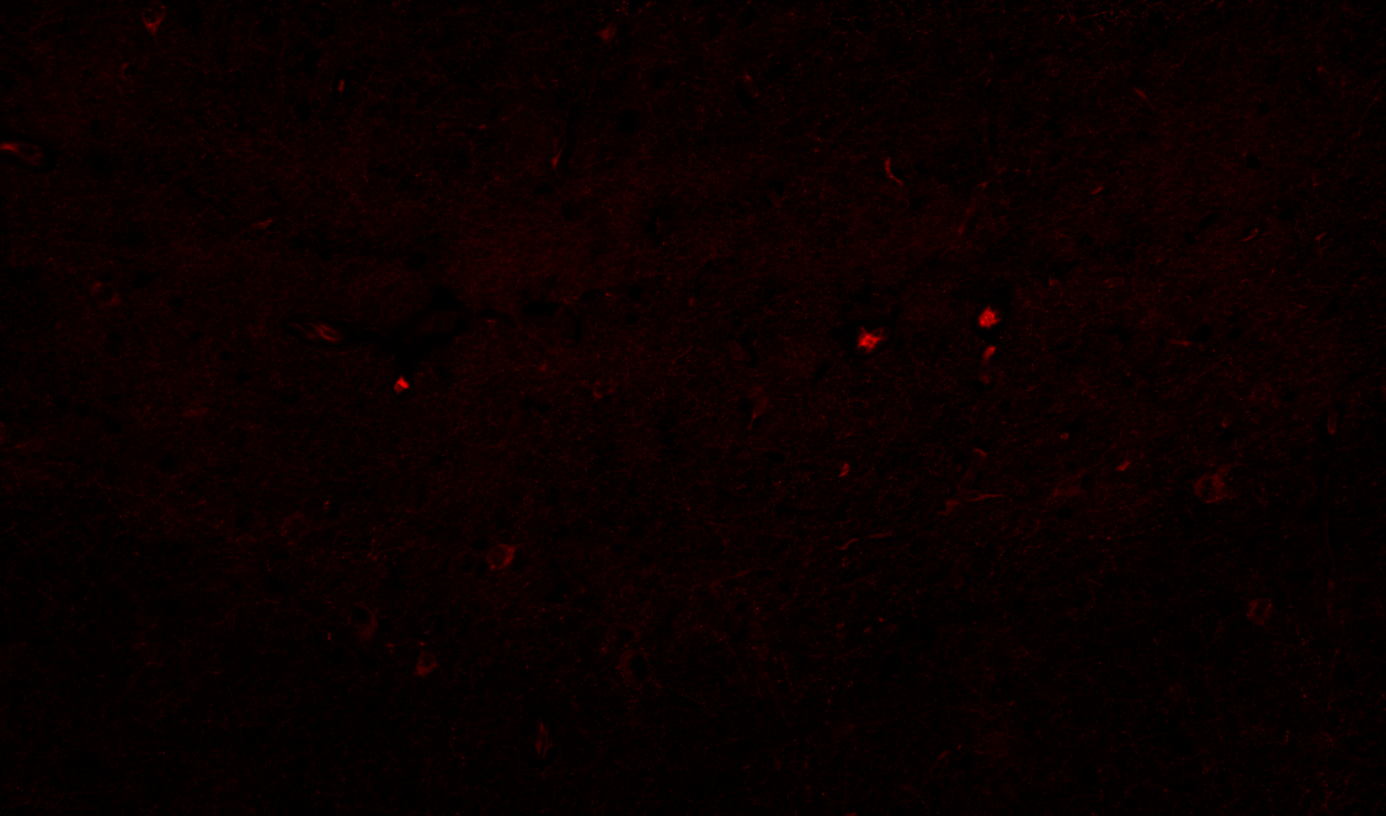

Supplement: S7 Data — (ZIP) [file pone.0200809.s007.zip › S7 Data/TG CA1 NPYGAD67/tg ca1 npygad67/ROI gad67 images/red roi gad67 images from npygad67coloc/mouse 3/redroi tg38 ca1 gad67npy001.nd2 - C=2.png]

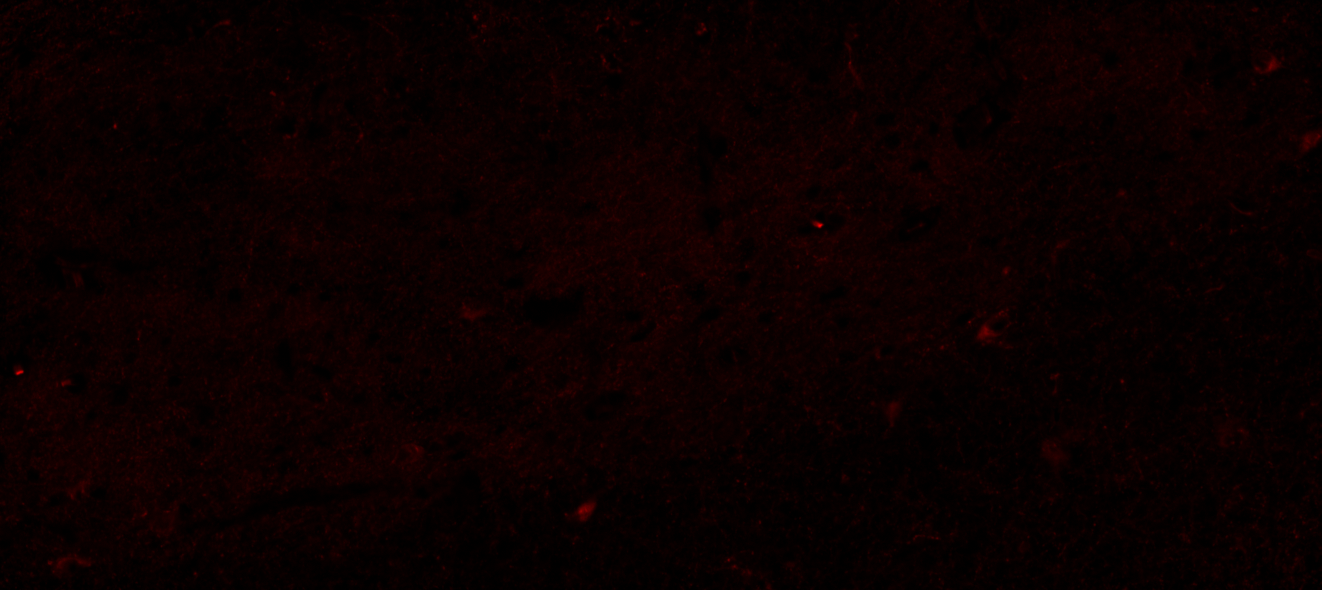

Supplement: S7 Data — (ZIP) [file pone.0200809.s007.zip › S7 Data/WT CA1 NPY GAD/mouse 1/red roi gad67 images mouse 1/redroi wt110 ca1 npygad67004.nd2 - C=2.png]

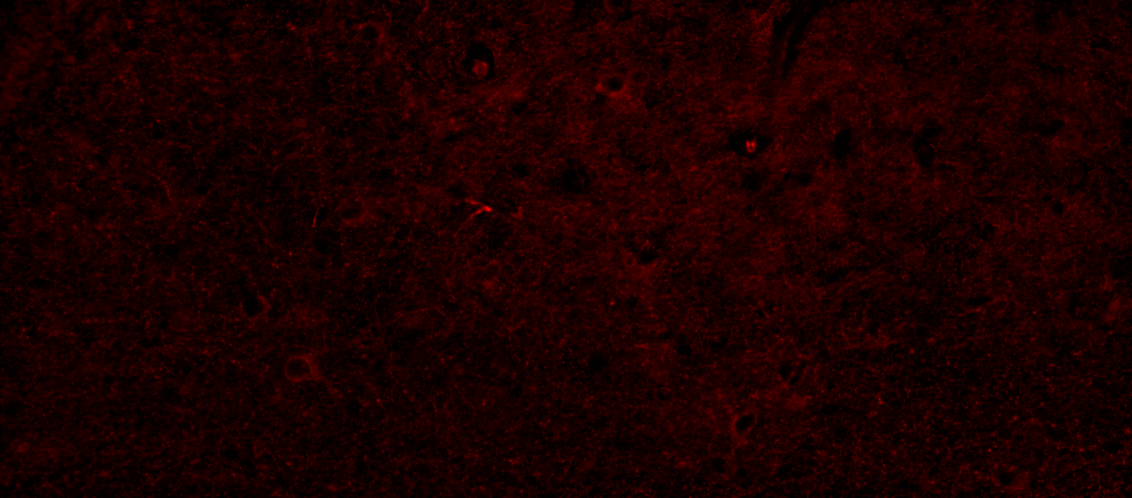

Supplement: S7 Data — (ZIP) [file pone.0200809.s007.zip › S7 Data/WT CA1 NPY GAD/mouse 1/red roi gad67 images mouse 1/redroi wt17 ca1 npygad67006.nd2 - C=2.png]

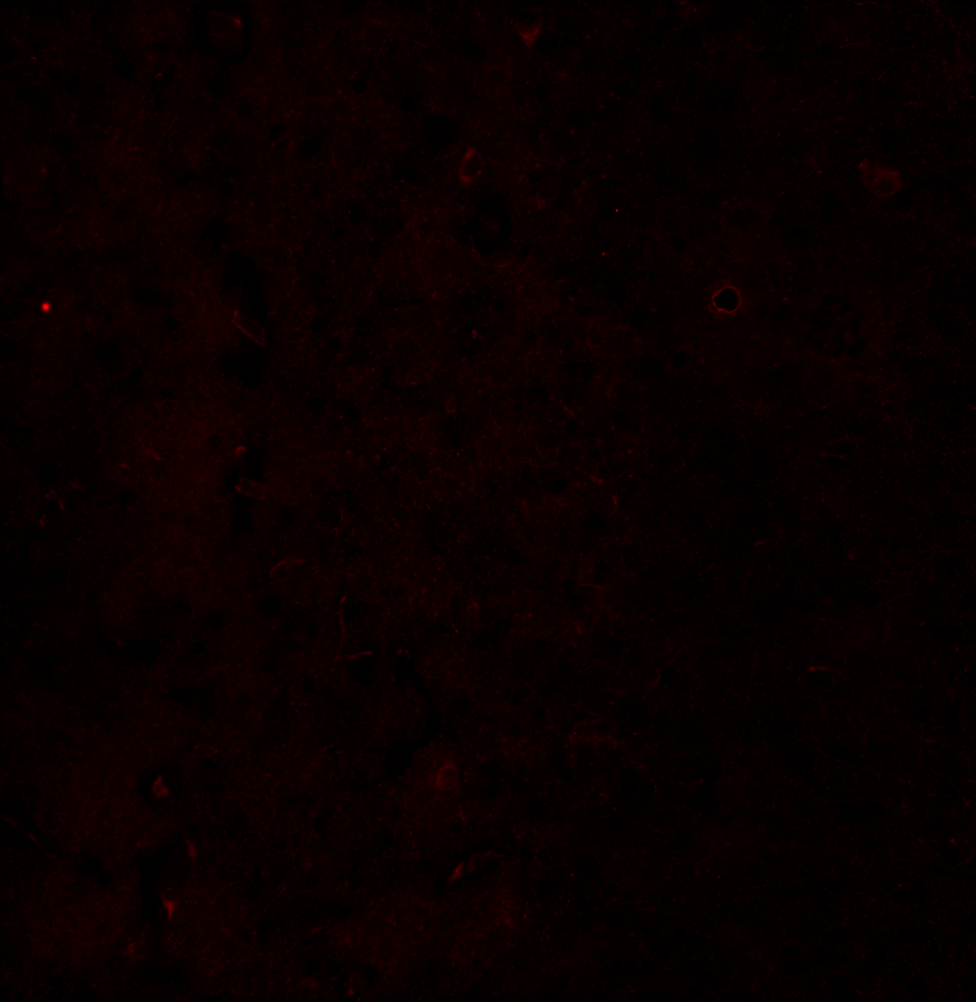

Supplement: S7 Data — (ZIP) [file pone.0200809.s007.zip › S7 Data/WT CA1 NPY GAD/mouse 1/red roi gad67 images mouse 1/redroitg110 ca1 gad67npy001.nd2 - C=2.png]

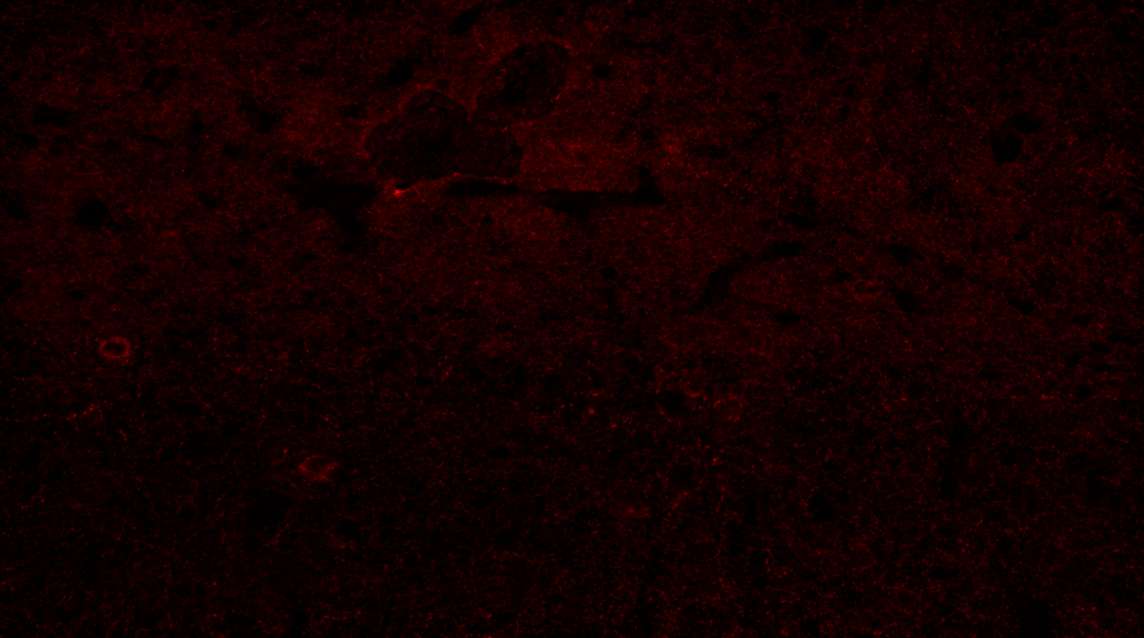

Supplement: S7 Data — (ZIP) [file pone.0200809.s007.zip › S7 Data/WT CA1 NPY GAD/mouse 2/red roi gad67 images/redroi wt218 ca1 npygad67002.nd2 - C=2.png]

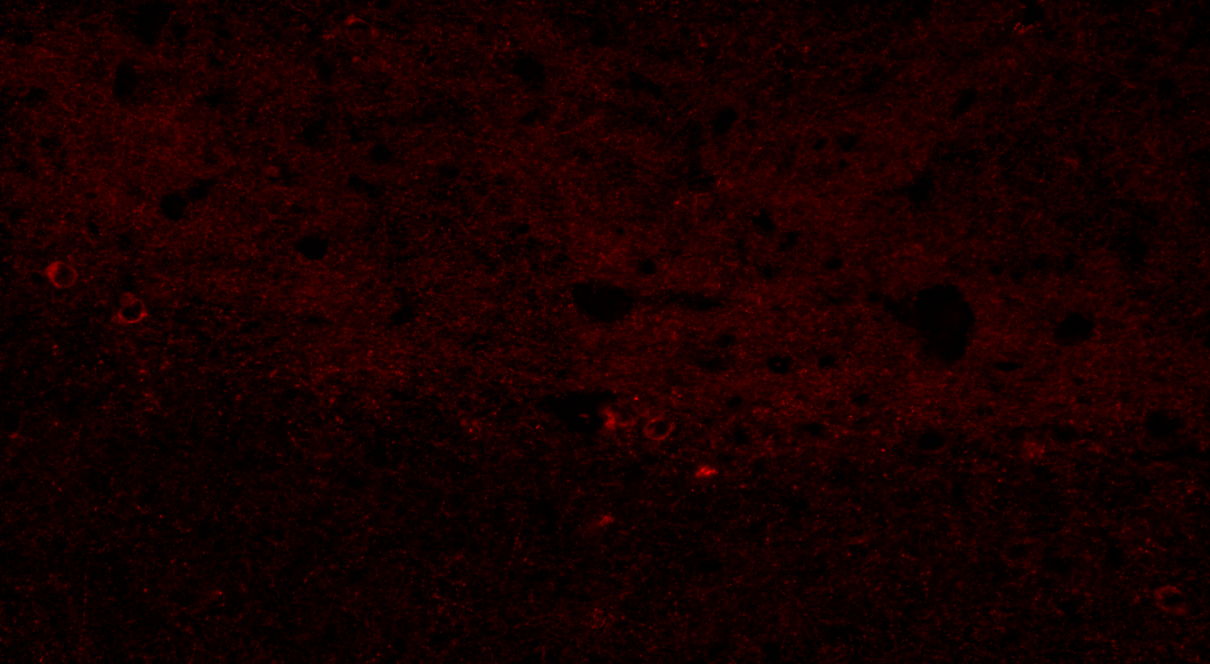

Supplement: S7 Data — (ZIP) [file pone.0200809.s007.zip › S7 Data/WT CA1 NPY GAD/mouse 2/red roi gad67 images/redroi wt26 ca1 npygad67003.nd2 - C=2.png]

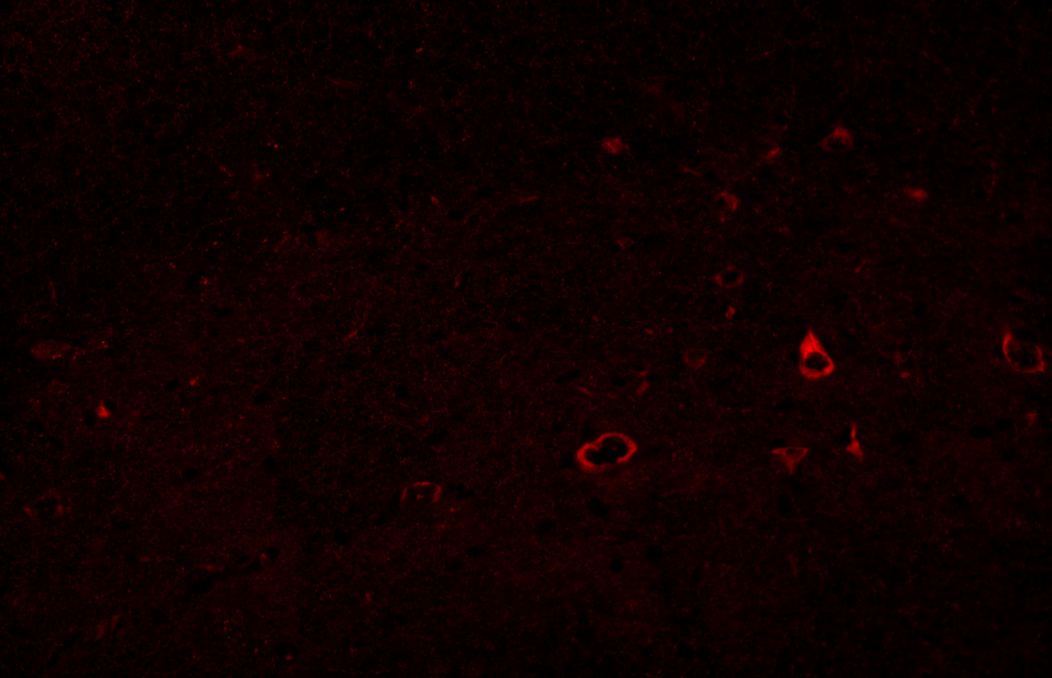

Supplement: S7 Data — (ZIP) [file pone.0200809.s007.zip › S7 Data/WT CA1 NPY GAD/mouse 3/red roi gad67 images/redroi wt310 ca1 npygad67001.nd2 - C=2.png]

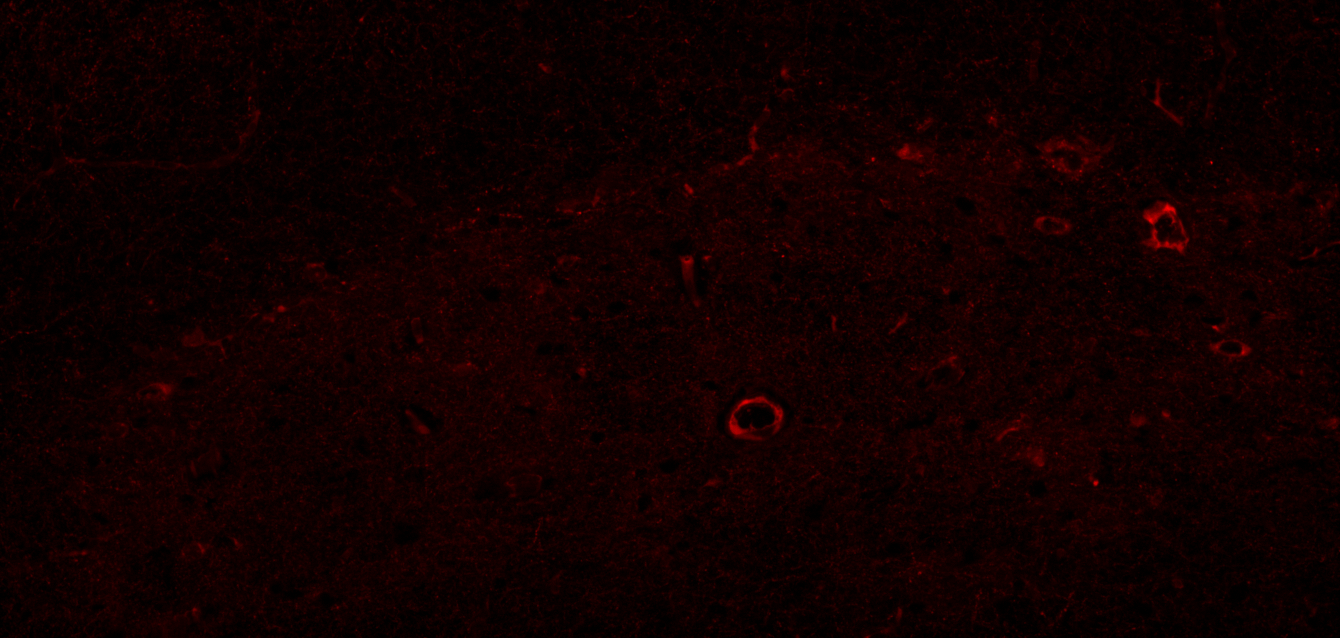

Supplement: S7 Data — (ZIP) [file pone.0200809.s007.zip › S7 Data/WT CA1 NPY GAD/mouse 3/red roi gad67 images/redroi wt38 ca1 npygad67.nd2 - C=2.png]

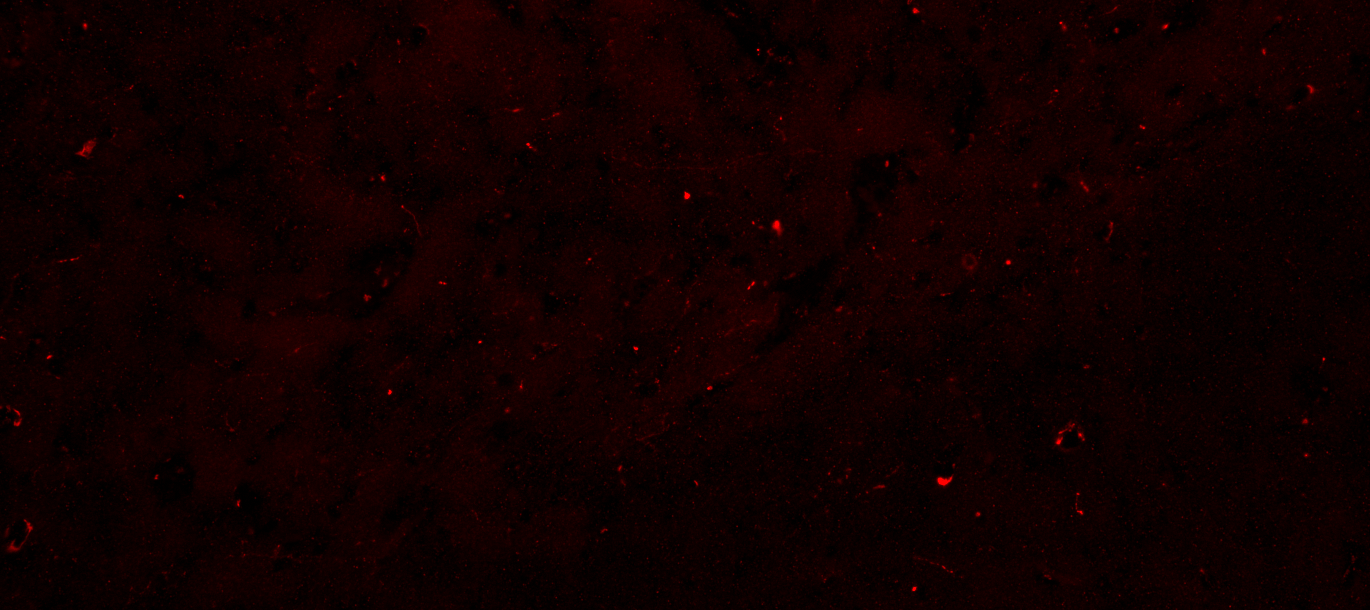

Supplement: S8 Data — (ZIP) [file pone.0200809.s008.zip › S8 Data/WT NPYGFP CA1/RED ROI OF NPY IN CA1/MOUSE 1/REDROI wt110 NPYGFP CA1009.nd2 - C=2.png]

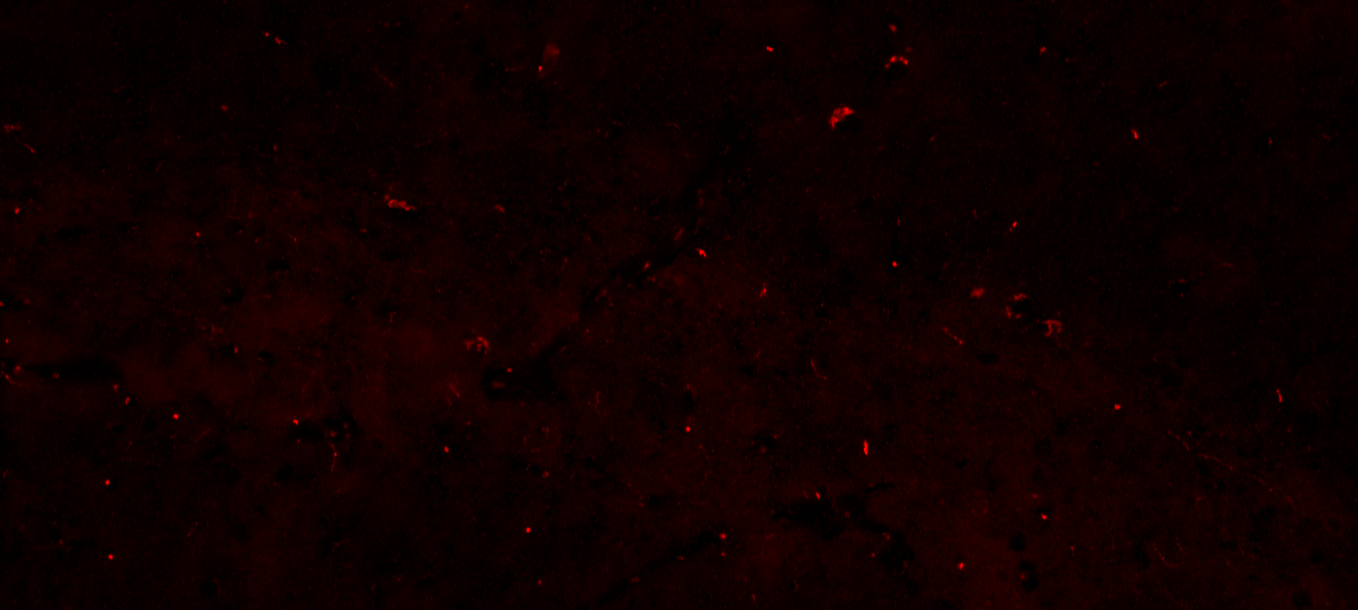

Supplement: S8 Data — (ZIP) [file pone.0200809.s008.zip › S8 Data/WT NPYGFP CA1/RED ROI OF NPY IN CA1/MOUSE 2/REDROI wt28 NPYGFP CA1012.nd2 - C=2.png]

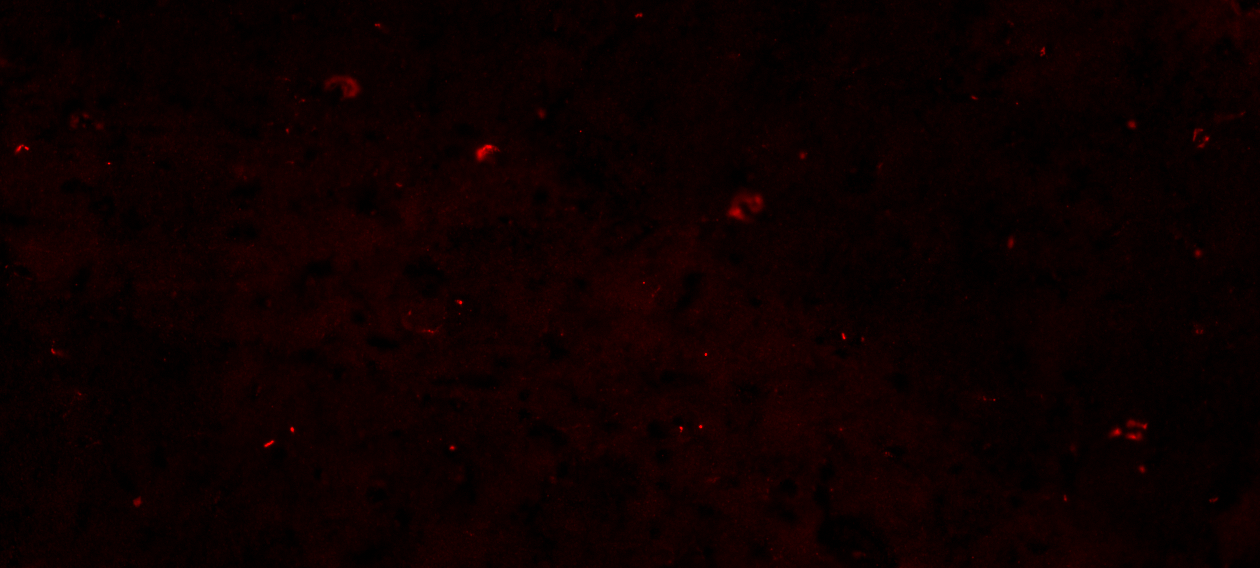

Supplement: S8 Data — (ZIP) [file pone.0200809.s008.zip › S8 Data/WT NPYGFP CA1/RED ROI OF NPY IN CA1/MOUSE 2/REDROI wt29 NPYGFP CA1013.nd2 - C=2.png]

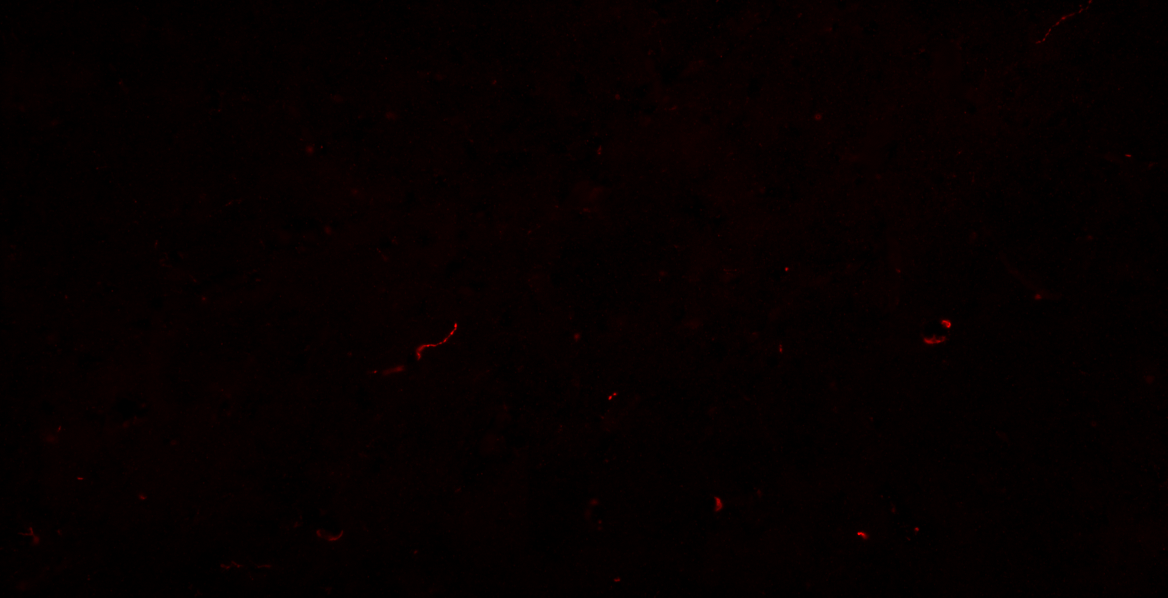

Supplement: S8 Data — (ZIP) [file pone.0200809.s008.zip › S8 Data/WT NPYGFP CA1/RED ROI OF NPY IN CA1/MOUSE 3/red roi wt39 NPYGFP CA1015.nd2 - C=2-1.png]

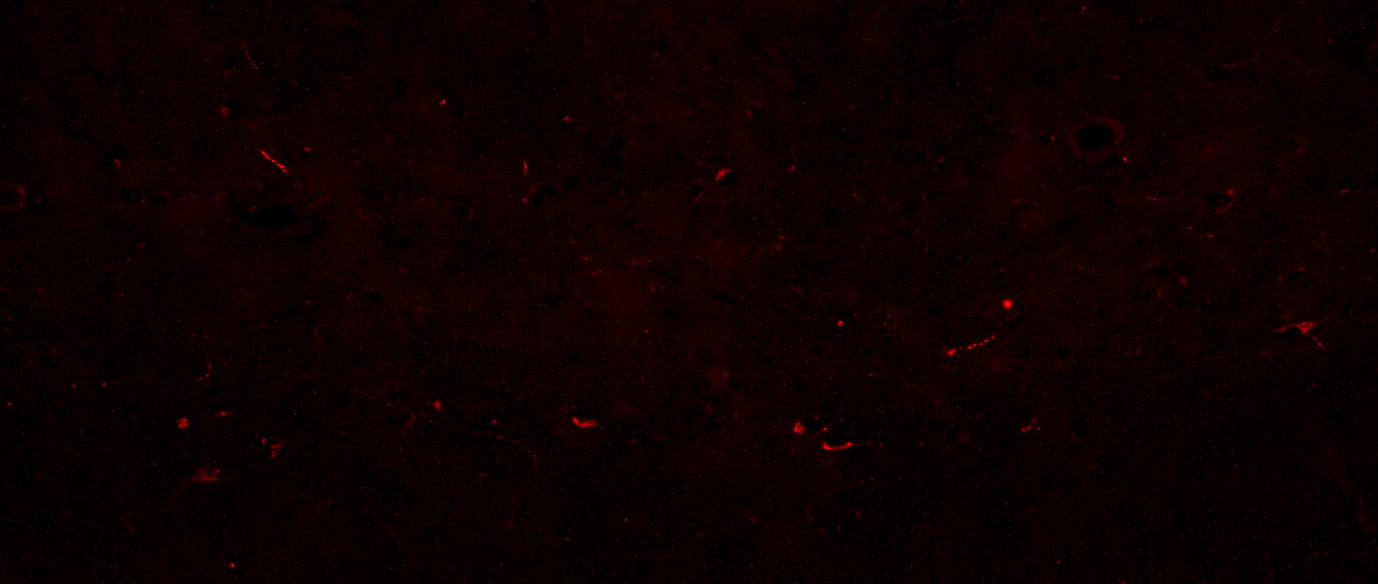

Supplement: S8 Data — (ZIP) [file pone.0200809.s008.zip › S8 Data/WT NPYGFP CA1/RED ROI OF NPY IN CA1/MOUSE 3/redroi wt38 NPYGFP CA1014.nd2 - C=2.png]

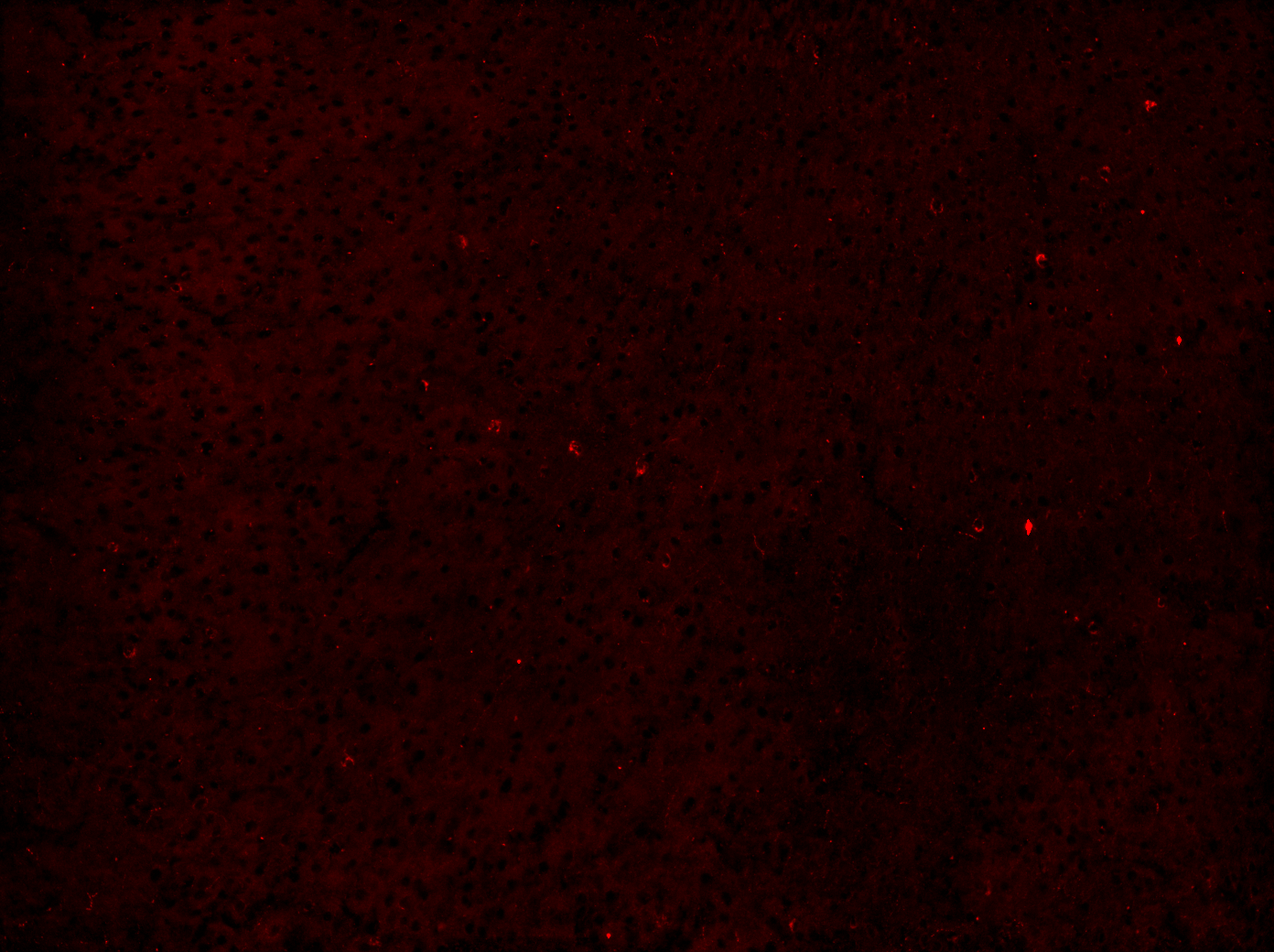

Supplement: S8 Data — (ZIP) [file pone.0200809.s008.zip › S8 Data/WT NPYGFP PFC/RED ROI NPY IMAGES/MOUSE1/REDROI WT12 NPYGFP PFC004.nd2 - C=2.png]

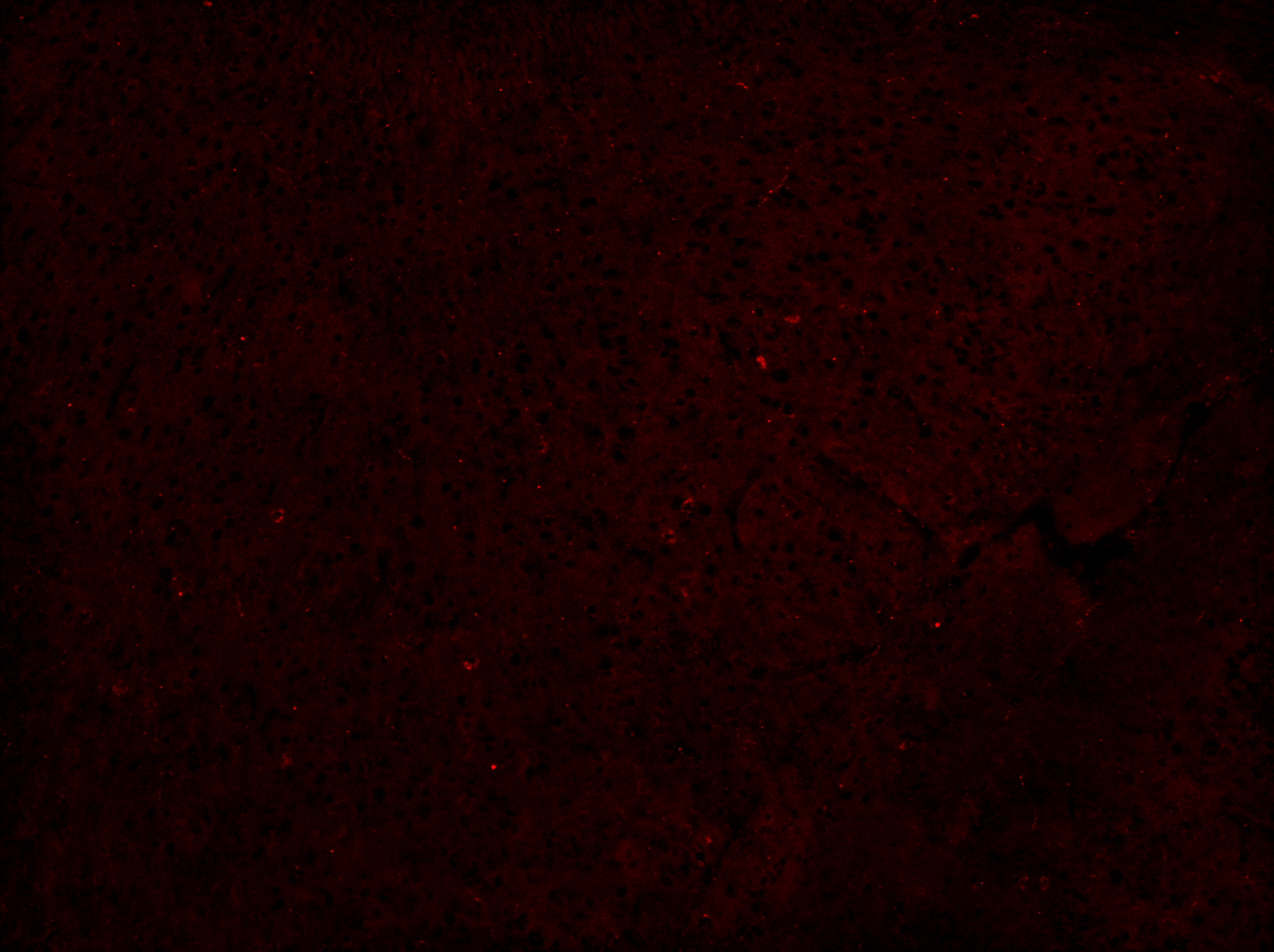

Supplement: S8 Data — (ZIP) [file pone.0200809.s008.zip › S8 Data/WT NPYGFP PFC/RED ROI NPY IMAGES/MOUSE2/RED ROIWT22 NPYGFP PFC005.nd2 - C=2.png]

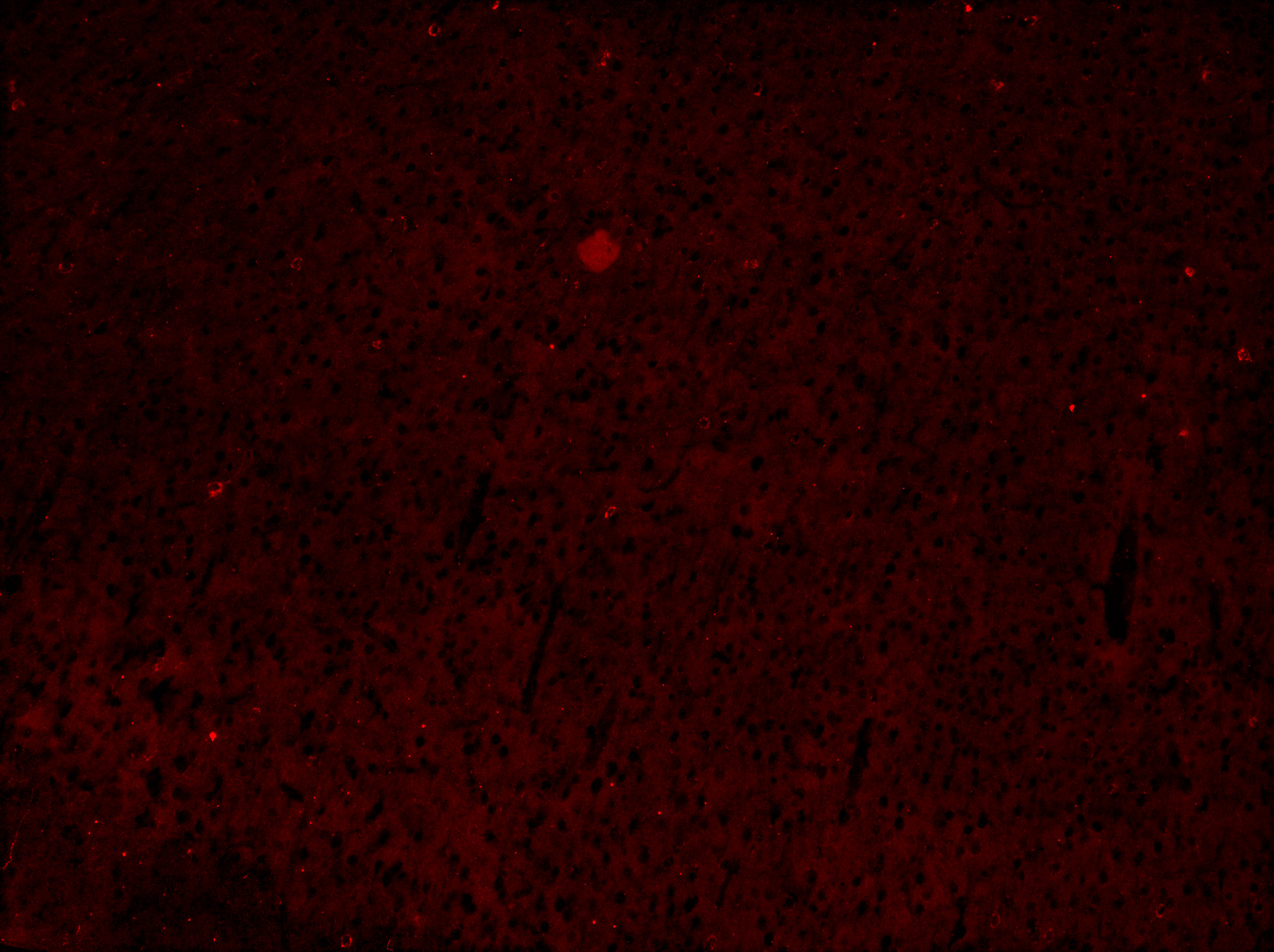

Supplement: S8 Data — (ZIP) [file pone.0200809.s008.zip › S8 Data/WT NPYGFP PFC/RED ROI NPY IMAGES/MOUSE2/REDROI WT23 NPYGFP PFC006.nd2 - C=2.png]

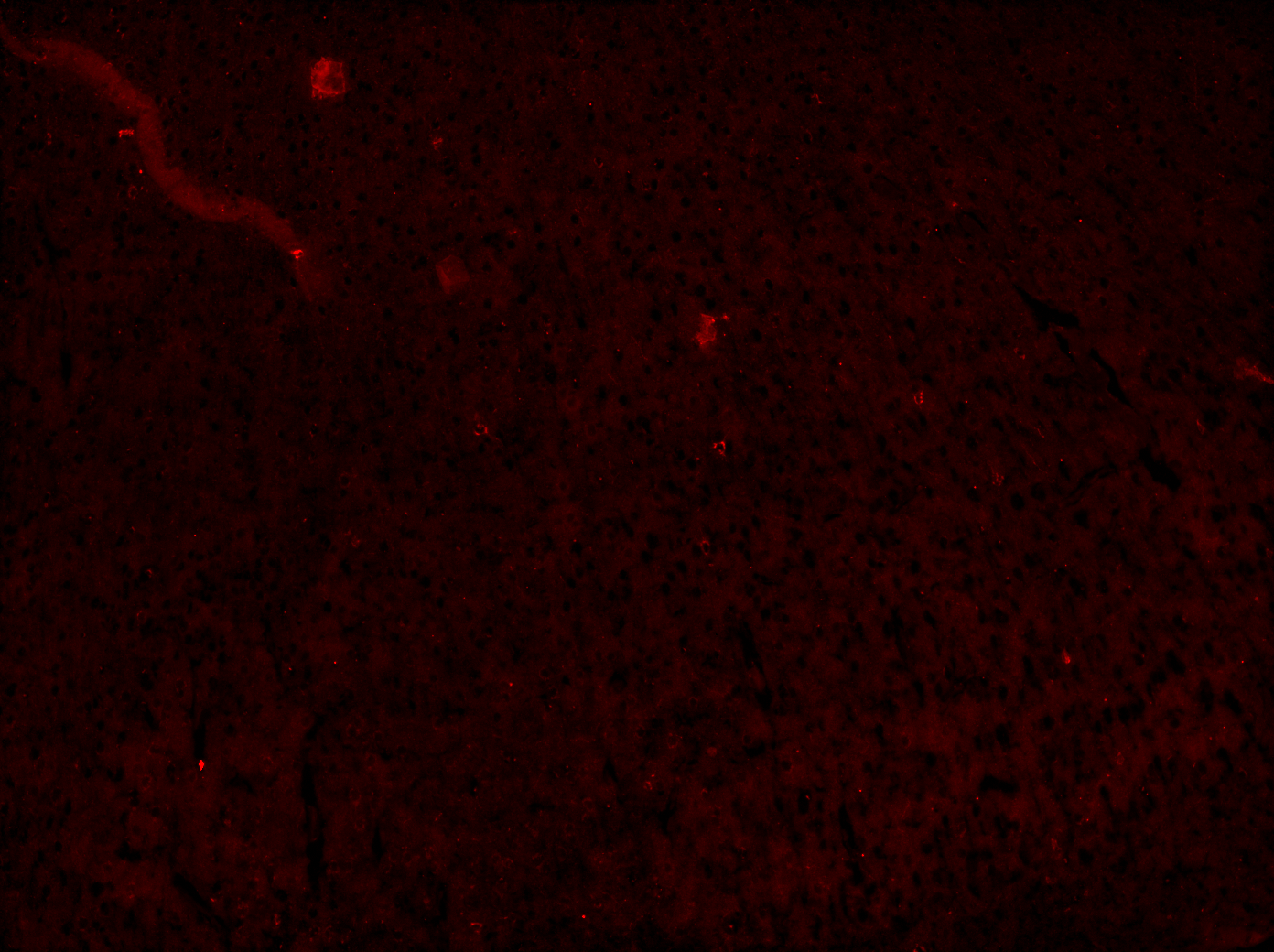

Supplement: S8 Data — (ZIP) [file pone.0200809.s008.zip › S8 Data/WT NPYGFP PFC/RED ROI NPY IMAGES/MOUSE3/REDROI WT31 NPYGFP PFC008.nd2 - C=2.png]

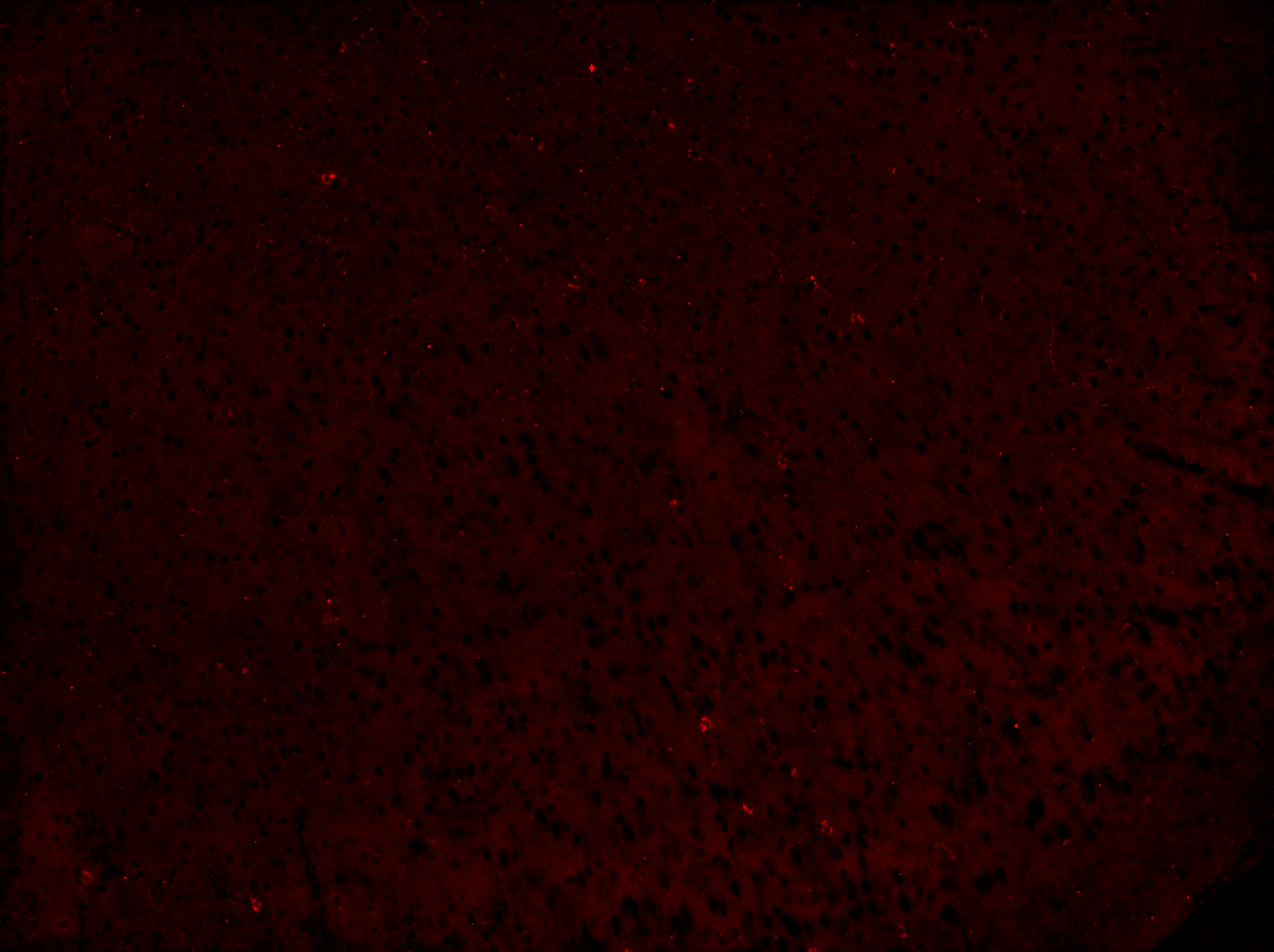

Supplement: S8 Data — (ZIP) [file pone.0200809.s008.zip › S8 Data/WT NPYGFP PFC/RED ROI NPY IMAGES/MOUSE3/REDROI WT32 NPYGFP PFC009.nd2 - C=2.png]

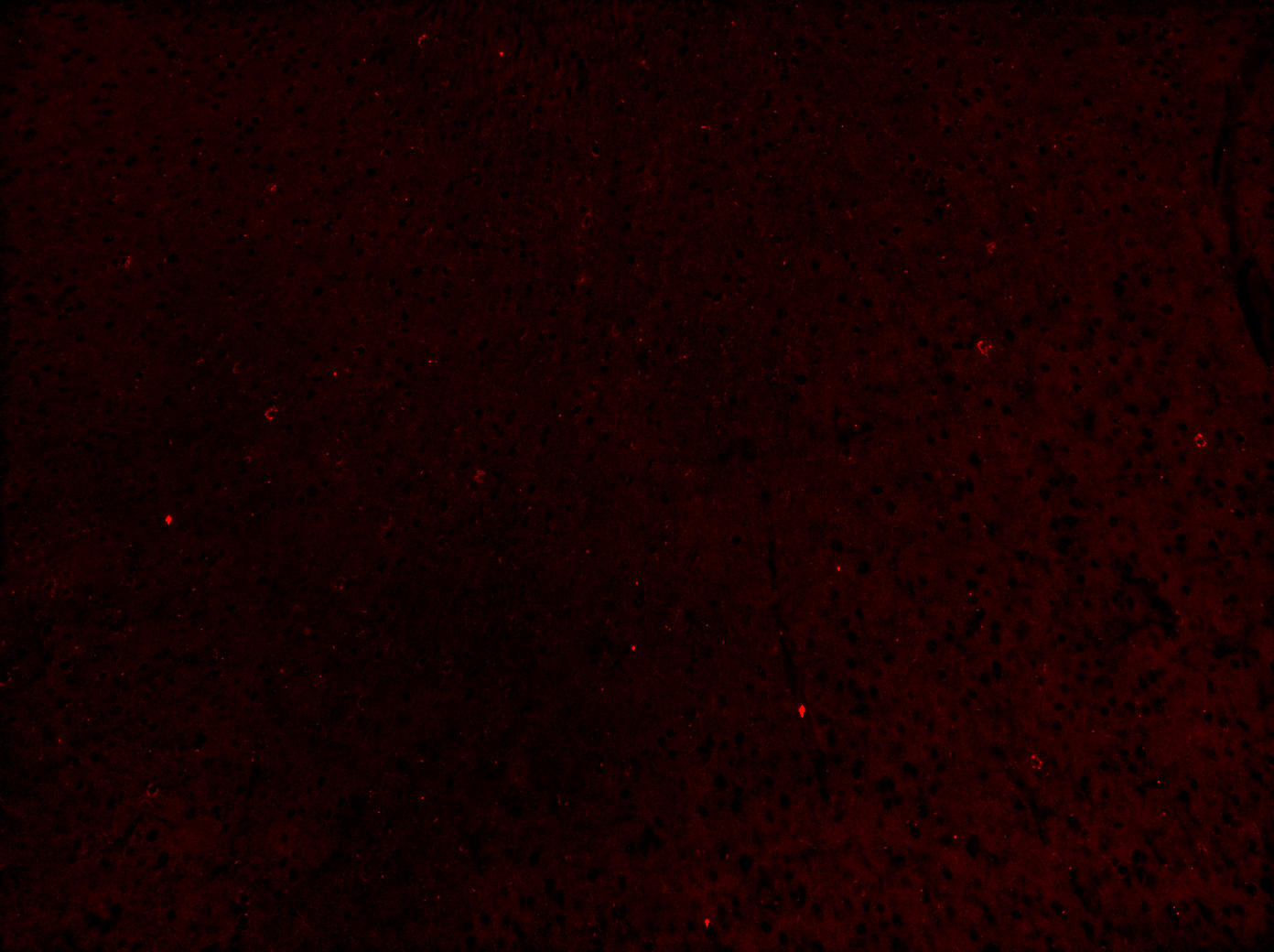

Supplement: S8 Data — (ZIP) [file pone.0200809.s008.zip › S8 Data/WT NPYGFP PFC/RED ROI NPY IMAGES/MOUSE3/REDROI WT33 NPYGFP PFC010.nd2 - C=2.png]
